# D2HGDH regulates alpha-ketoglutarate levels and dioxygenase function by modulating IDH2

**DOI:** 10.1038/ncomms8768

**Published:** 2015-07-16

**Authors:** An-Ping Lin, Saman Abbas, Sang-Woo Kim, Manoela Ortega, Hakim Bouamar, Yissela Escobedo, Prakash Varadarajan, Yuejuan Qin, Jessica Sudderth, Eduard Schulz, Alexander Deutsch, Sumitra Mohan, Peter Ulz, Peter Neumeister, Dinesh Rakheja, Xiaoli Gao, Andrew Hinck, Susan T. Weintraub, Ralph J. DeBerardinis, Heinz Sill, Patricia L. M. Dahia, Ricardo C. T. Aguiar

**Affiliations:** 1 Division of Hematology and Medical Oncology, Department of Medicine, University of Texas Health Science Center at San Antonio, San Antonio, Texas 78229, USA; 2 Department of Pediatrics, Children’s Medical Center Research Institute, University of Texas Southwestern, Dallas, Texas 75390, USA; 3 Division of Hematology, Medical University of Graz, A-8036 Graz, Austria; 4 Institute of Human Genetics, Medical University of Graz, A-8036 Graz, Austria; 5 Department of Pathology and Pediatrics, University of Texas Southwestern Medical Center, Dallas, Texas 75390, USA; 6 Department of Biochemistry, University of Texas Health Science Center at San Antonio, San Antonio, Texas 78229, USA; 7 Greehey Children’s Cancer Research Institute, University of Texas Health Sciences Center at San Antonio, San Antonio, Texas 78229, USA; 8 Cancer Therapy and Research Center, University of Texas Health Science Center at San Antonio, San Antonio, Texas 78229, USA; 9 South Texas Veterans Health Care System, Audie Murphy VA Hospital, San Antonio, Texas 78229, USA

## Abstract

Isocitrate dehydrogenases (IDH) convert isocitrate to alpha-ketoglutarate (α-KG). In cancer, mutant IDH1/2 reduces α-KG to D2-hydroxyglutarate (D2-HG) disrupting α-KG-dependent dioxygenases. However, the physiological relevance of controlling the interconversion of D2‐HG into α‐KG, mediated by D2‐hydroxyglutarate dehydrogenase (D2HGDH), remains obscure. Here we show that wild-type D2HGDH elevates α-KG levels, influencing histone and DNA methylation, and HIF1α hydroxylation. Conversely, the D2HGDH mutants that we find in diffuse large B-cell lymphoma are enzymatically inert. D2-HG is a low-abundance metabolite, but we show that it can meaningfully elevate α-KG levels by positively modulating mitochondrial IDH activity and inducing IDH2 expression. Accordingly, genetic depletion of IDH2 abrogates D2HGDH effects, whereas ectopic IDH2 rescues D2HGDH-deficient cells. Our data link D2HGDH to cancer and describe an additional role for the enzyme: the regulation of IDH2 activity and α-KG-mediated epigenetic remodelling. These data further expose the intricacies of mitochondrial metabolism and inform on the pathogenesis of D2HGDH-deficient diseases.

## Abstract (web summary)

IDH1- and IDH2-mutant cancer cells aberrantly accumulate D2-hydroxyglutarate (D2-HG). Here, Lin *et al*. find loss-of-function mutations in D2-hydroxyglutarate dehydrogenase (D2HGDH), which converts D2-HG to alpha-ketoglutarate (α-KG), in diffuse large B-cell lymphomas and show that D2HGDH via α-KG regulates the expression and activity of IDH2.

## Graphical abstract

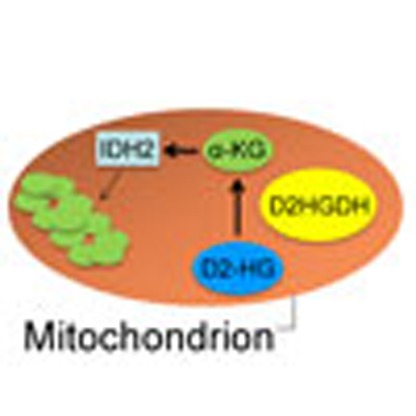

Isocitrate dehydrogenases (IDH) catalyse the reversible conversion of isocitrate to alpha-ketoglutarate (α-KG). Mutant IDH1/2 creates a neomorphic enzyme that reduces α-KG to the structurally related D2-hydroxyglutarate (D2-HG)^1^,^2^,^3^. This metabolic deregulation impinges on the activity of multiple α-KG-dependent dioxigenases^4^, and induces epigenetic changes that are proposed to play a role in the pathogenesis of IDH1/2 mutant cancers^5^,^6^,^7^. These observations also suggest the need for a tight physiological control of the cellular levels of α-KG and D2-HG. The interconversion of D2‐HG into α‐KG is mediated by D2‐hydroxyglutarate dehydrogenase (D2HGDH)^8^. Under normal conditions, D2-HG is considered an unwanted byproduct of cellular metabolism with no known physiologic role. Thus, the current thought is that D2HGDH primarily functions to prevent the potentially deleterious cellular accumulation of D2-HG^9^. In agreement with this concept, loss-of-function mutation and deletion of *D2HGDH* causes a severe autosomal recessive neurometabolic disorder, type I D-2-hydroxyglutaric aciduria (D-2-HGA)^10^. However, the relevance of D2HGDH’s activity to mitochondrial metabolism and its putative ability to promote an oncogenic metabolic deregulation remains underexplored.

There are two possible enantiomers of 2-HG. In addition to D2-HG, which is converted to α-KG by D2HGDH, there is L2-HG, which has its cellular accumulation prevented by the activity of the dehydrogenase L2HGDH^10^. Similar to D2HGDH, hereditary loss of L2HGDH also causes a neurometabolic disorder with an autosomal recessive mode of inheritance, L-2-hydroxyglutaric aciduria (L-2-HGA)^11^,^12^. Interestingly, patients diagnosed with L-2-HGA appear to be at a higher risk of developing brain tumours than matching control populations^13^. Further, recent evidence has implicated reduced expression of L2HGDH as a somatic event in renal cell carcinoma, which resulted in dioxygenase-related epigenetic deregulation^14^. Thus, in addition to the accumulation of D2-HG in IDH1/2 mutant tumours, the related metabolite, L2-HG, has also been linked to cancer. In both instances, the resulting metabolic imbalance yielded an epigenetic phenotype that was related to the deregulation of α-KG-dependent dioxygenase. These observations suggest that an in-depth examination of this metabolic axis is warranted in diseases associated with significant epigenetic remodelling.

The elucidation of the genetic landscape of diffuse large B-cell lymphoma (DLBCL) highlighted a previously unappreciated role of epigenetic modifiers in the pathogenesis of this disease. Regulators of histone methylation and acetylation, as well as DNA methylation, including MLL2, EZH2, CREBBP, EP300, TET2 and TET1, have been found to be somatically mutated, deleted or epigenetically silenced in these tumours^15^,^16^,^17^,^18^,^19^,^20^. Concrete evidence for the importance of these epigenetic modifiers to B-cell lymphoma biology has also been mounting. Aberrant lymphoid differentiation has been reported in Tet2 null mice^19^, and frank B-cell lymphoma has been recently described upon deletion of Tet1 *in vivo*^20^. These observations are relevant in the context of the present work because a significant fraction of the enzymes that regulate histone and DNA methylation (for example, Jumonji histone demethylases (HDMs) and TET DNA hydroxylases) belong to the family of α-KG-dependent dioxygenases, which can be functionally inactivated by α-KG-related metabolic imbalances. Thus, although mature B-cell malignancies do not harbour D2-HG producing *IDH1/IDH2* mutations^21^, the presence of other genetic defects, such as loss of D2HGDH, that may cause actual or relative (competitive) α-KG deficiency and aberrant epigenetic remodelling has not been examined in depth.

Here, we show that somatic, truncating and missense, heterozygous *D2HGDH* mutations are present in a small subset of DLBCL. The DLBCL-associated *D2HGDH* mutations target the same protein domains disrupted in the autosomal recessive type I D-2-HGA. Detailed enzymatic and cellular examination defined these variants as loss-of-function. The principal consequence of the partial loss of D2HGDH in DLBCL is a significant decrease in the cellular levels of α-KG, not massive accumulation of D2-HG. Using genetic models of physiologic increment or downregulation of D2HGDH, we show that subtle modulation of D2HGDH significantly influences histone and DNA methylation, and HIF1α hydroxylation. These effects are dependent on α-KG, and can be mimicked with a synthetic cell-permeable α-KG or abrogated with its competitive inhibitor dimethyloxalylglycine (DMOG). Importantly, we show that D2HGDH meaningfully contribute to the cellular pool of α-KG by regulating IDH activity in the mitochondria, but not in the cytosol, in association with transcriptional induction of IDH2. Accordingly, genetic modulation of mitochondrial IDH2 rescues the effects of D2HGDH on histone and DNA methylation, and HIF1α hydroxylation. Together, these findings link D2HGDH to epigenetic remodelling in DLBCL and indicate that this enzyme is not simply a guardian against the accumulation of toxic D2-HG. Instead, our data suggest that by regulating IDH2, D2HGDH is an important player in the generation of α-KG, a metabolite that coordinates epigenetic plasticity in various model systems and influences malignant behaviour, longevity and stem cell maintenance^22^,^23^.

## Results

### DLBCL-associated D2HGDH mutations

**Figure 1:**
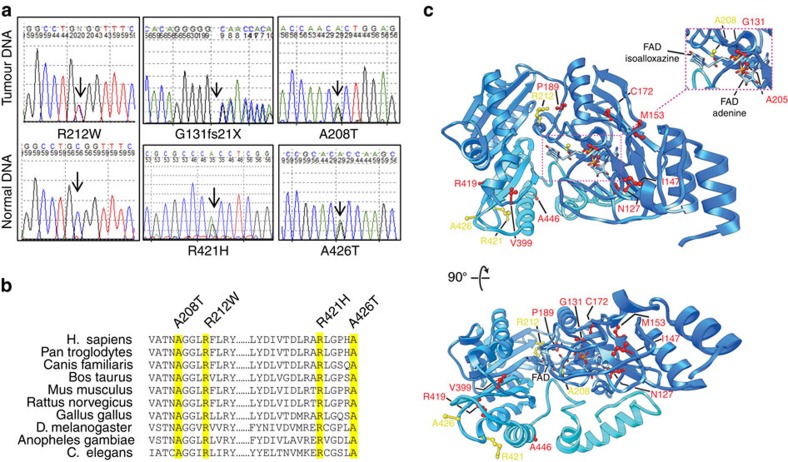
D2HGDH mutations in DLBCL. (**a**) Sequencing traces representative of each of the five unique mutations found in DLBCL; arrows indicate the nucleotide change, and amino acid substitution is listed at the bottom. Constitutive DNA was available from two patients with D2HGDH mutant tumours (R212W variant), and one case is shown here. The G131fs21X, A208T and R421H mutations were found in cell lines, whereas A426T was found both in primary tumours and cell lines (Supplementary Table 1). (**b**) All four missense variants identified in DLBCL map to fully conserved residues. (**c**) Display of the closest structural homologue of human D2HGDH (dehydrogenase from Rhodopseudomonas palustris, PDB 3PM9) identified in the RCSB protein data bank. Orthogonal views of the structure are shown, with the non-covalently bound FAD displayed, as well as sidechains of the residues targeted by missense mutations in DLBCL (shaded yellow) or in D-2-hydroxyglutaric aciduria (shaded red). In both diseases, the mutations clustered to two structural areas—a region of possible contact with the FAD and/or the enzyme’s substrate (see expansion subpanel), or an uncharacterized outside loop containing the residues V399, R419, R421, A426 and A446. The structures shown were generated using the programme UCSF Chimera32.

Considering the important role of α-KG in regulating the activity of dioxygenases, we sought to identify α-KG-dependent models for deregulation of epigenetic modifiers that are relevant to DLBCL biology. We centred our investigation on the genes encoding the enzymes that convert 2-HG into α-KG^9^, *D2HGDH* and *L2HGDH*. We sequenced the entire coding region and intron/exon boundaries of *D2HGDH* and *L2HGDH* in an initial cohort of 69 DLBCLs and found six samples (8.7%) with four unique heterozygous mutations in *D2HGDH* (three missense, one truncating), but none in *L2HGDH*. To expand on this observation, we sequenced *D2HGDH* in an additional 80 DLBCL samples, and found another missense mutation in two independent tumours. In all, five unique variants (four missense and one truncating deletion) were found in 8 of 149 samples (5.3%), including 4/120 primary tumours and 4/29 DLBCL cell lines (Fig. 1a and Supplementary Table 1). In all instances, the mutations were heterozygous, and the mutant and wild-type alleles were equally expressed (Supplementary Fig. 1). The *IDH1/2* exons that encode the 2HG-producing mutations (G97, Y100 and R132, IDH1; R140 and R172, IDH2)^24^ were also sequenced in all *D2HGDH*-mutant tumours, alongside 74 other DLBCL cases, and were found to be in the wild-type (WT) configuration. We confirmed the somatic nature of the *D2HGDH* mutations in two primary tumours, but did not have constitutive DNA to examine the other cases. All *D2HGDH* mutations found in DLBCL target conserved residues and cluster either at the point of D2HGDH contact with its co-factor, FAD, or in the less well-characterized carboxy (C)-terminal region, a distribution similar to that found in patients with D-2-HGA (Fig. 1b,c)^25^. The molecular basis of this hereditary syndrome also includes small deletions at the *D2HGDH* locus. Therefore, we used multiplex ligation‐dependent probe amplification (MLPA) and real‐time quantitative PCR (qPCR) to examine the copy-number integrity of this gene in our cohort, but found no evidence for small intragenic deletion of *D2HGDH* in DLBCL (Supplementary Table 2). None of the DLBCL-associated *D2HGDH* variants that we found were present in ∼300 normal alleles sequenced in our laboratory. However, the Exome Aggregation Consortium—EXAC database, which includes both normal and disease alleles from over 61,000 individuals, lists two of the variants that we identified (A426T and R421H) as rare SNPs (allele frequencies of 0.01 and 0.00022%, respectively). These variants were found at a much higher frequency (2.8 and 0.67%, respectively) in our cohort (Supplementary Table 1) and, as we show below, they are functionally impaired. In addition, one of these two variants, A426T, has been previously reported in a family of D-2-HGA^25^.

D2HGDH is a FAD-dependent mitochondrial enzyme^8^, but little else is known about its functional requirements. Thus, to explore the possibility that D2HGDH forms homo-complexes, a feature common to metabolic mitochondrial enzymes, and to explore the possibility that the DLBCL-associated mutations could alter this functional requirement, we generated D2HGDH WT and mutant constructs tagged with either FLAG or HA. Co-transfection of differently tagged D2HGDH WT–WT pairs, followed by bidirectional immunoprecipitation (IP) unveiled a hitherto unreported feature of this protein, its ability to form homodimers and/or multimers (Supplementary Fig. 2). However, co-transfection and IP examinations of mutant D2HGDH proteins demonstrated that these variants did not change the ability of D2HGDH to self-complex. We also examined whether the DLBCL-associated mutations changed the subcellular localization of D2HGDH. Using immunofluorescence, we confirmed that D2HGDH localizes to the mitochondria and demonstrated that the mutant enzymes do not display a distinct subcellular localization (Supplementary Fig. 3).

### D2HGDH mutations are loss-of-function

**Figure 2:**
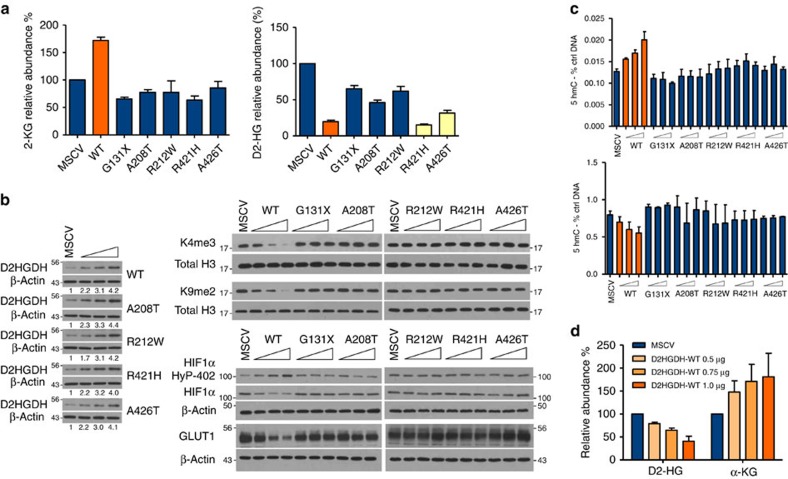
D2HGDH modulates D2-HG and α-KG levels and the readout of dioxygenases function. (**a**) Left: LC/MS show that WT-D2HGDH cells have significantly higher α-KG levels than empty-MSCV or mutant D2HGDH expressing isogenic cells (*P*<0.0001, ANOVA; *P*<0.05 Bonferroni’s multiple comparison post test). Right: expression of WT D2HGDH significantly lowered D2-HG levels in comparison with cells expressing and empty-MSCV or the G131X, A208T and R212W mutants, but not R421H or A426T (yellow bars) (*P*<0.0001, ANOVA). (**b**) Left: western blots of D2HGDH in cells transiently transfected with an empty vector (MSCV—1 μg) or increasing amounts (0.5 μg, 0.75 μg and 1 μg) of the WT or mutant enzymes—densitometric quantification confirms the progressive elevation of D2HGDH levels. Right top: western blots of H3K4me3 and H3K9me2 show a progressive decrease in methylation in cells expressing the WT D2HGDH but not the mutant enzymes; right bottom: in hypoxia, expression of WT D2HGDH increases HIF1α hydroxylation (Pro-402), with decrease in its stability, and expression of the transcriptional target GLUT1; expression of mutant enzymes has no effects on HIF1α hydroxylation/expression. (**c**) WT-D2HGDH cells display a significantly higher abundance of 5hmC marks (top panel, *P*<0.0001, ANOVA) and concomitant decrease in global DNA methylation (bottom panel, *P*=0.035, ANOVA). Expression of mutant D2HGDH did not significantly influence 5hmC or 5mC levels. (**d**) Transient expression of WT D2HGDH significantly decreased D2-HG and increased α-KG levels in a dose-dependent manner (*P*=0.006 and *P*=0.0002, respectively, ANOVA). The data shown in **a** represent the mean and s.d. of assays performed with four or five replicates per sample type, and displayed as relative levels to control cells (MSCV). The transient transfection assays (**b**–**d**) were performed three to four times. The data shown in **c** represent mean and s.d. of five data points derived from two biological replicates. The data shown in **d** represent the mean and s.d. of an assay performed in triplicate, displayed as relative levels to control cells (MSCV); the result from an independent biological replicate is shown in Supplementary Fig. 6.

To determine whether the five DLBCL-associated D2HGDH variants that we discovered affected the conversion of D2-HG into α-KG, we generated HEK-293 cells stably expressing the WT and mutant enzymes and used liquid chromatography-mass spectrometry (LC-MS) to quantify these metabolites. Cells expressing WT D2HGDH displayed significantly elevated α-KG levels, whereas cells expressing the G131X, A208T, R212W, R421H or A426T mutant constructs were indistinguishable from the isogenic empty vector control (*P*<0.0001, analysis of variance (ANOVA)) (Fig. 2a and Supplementary Figure 4). In agreement with these data, the levels of D2-HG were significantly lower in cells expressing the WT enzyme than those with empty MSCV or the D2HGDH mutants G131X, A208T and R212W (*P*<0.0001, ANOVA). Surprisingly, despite the inability to effectively generate α-KG, the levels of D2-HG in cells expressing the R421H or A426T mutants were similar to those of the D2HGDH-WT counterparts (Fig. 2a, Supplementary Fig. 4); the reason for this discrepancy remains unclear. Importantly, as expected, ectopic expression of WT or mutant D2HGDH did not alter L2-HG levels (Supplementary Fig. 4), further suggesting that metabolism of the 2-HG enantiomers is not tightly linked.

We next explored the consequences of this metabolic disruption towards α-KG-dependent dioxygenases. To that end, and to avoid the potentially confounding effect of stable overexpression models, we transiently expressed increasing (physiologic) amounts of WT and mutant D2HGDH (Fig. 2b and Supplementary Fig. 5). Examining these models, we found that WT D2HGDH, but none of the mutants, induced a ‘dose-dependent’ decrease in histone H3 lysine (H3K) methylation, an increase in HIF-1α hydroxylation at proline 402 (with corresponding decrease in total HIF1α levels), and a significant increase in the abundance of 5hmC (5-hydroxy-methyl-cytosine) marks, with corresponding decrease in global 5-methyl-cytosine (5mC) levels (Fig. 2b,c).These data confirm that the DLBCL-associated mutants encode an inert enzyme We attributed the cellular effects of WT D2HGDH to a heightened activity of α-KG-dependent histone demethylases (HDMs), TET enzymes and HIF1α prolyl-hydroxylases, a consequence of the higher levels of α-KG in these cells. Indeed, we were able to confirm that the subtle increase in WT D2HGDH expression noted in this model resulted in a progressive increase and decrease of α-KG and D2-HG levels, respectively (Fig. 2d and Supplementary Fig. 6). Finally, as expected, the distinction between WT and mutant D2HGDH towards histone/DNA methylation and HIF1 hydroxylation was also observed in the models of stable expression (Supplementary Fig. 7). Of note, recent data generated in the context of IDH1/2 mutant cell lines confirmed the ability of D2HGDH to increases α-KG and to modulate the activity of a subset of α-KG-dependent dioxygenases^26^.

**Figure 3:**
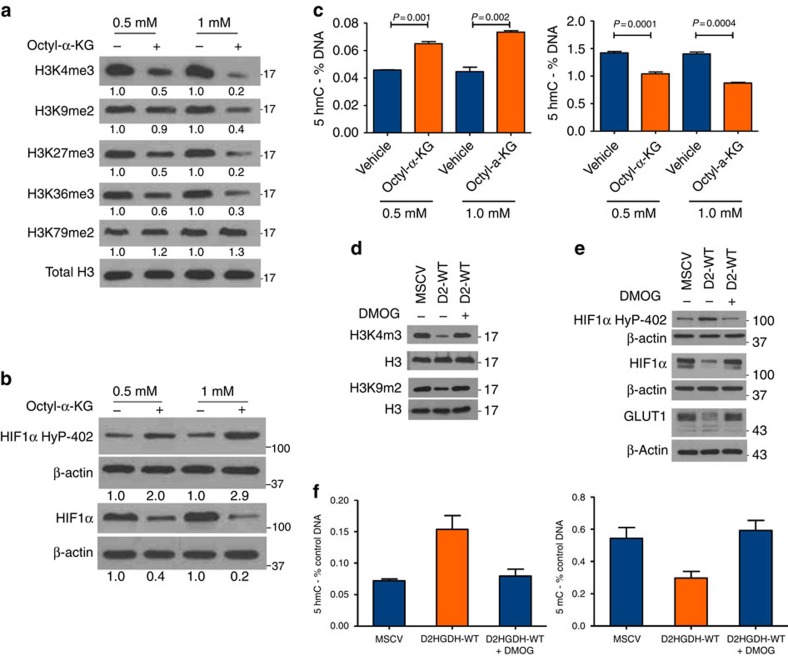
α-KG mediates the cellular effects of wild-type D2HGDH expression. (**a**) Methylation of H3 lysine residues was determined by western blot in HEK‐293 exposed to 0.5 mM or 1 mM of octyl-α-KG (or vehicle control) for 6 h. Octyl-α-KG suppressed K4, K9, K27, K36 methylation in a dose-dependent manner. No changes were found in H3K79me2 levels confirming that this residue is not regulated by an α-KG-dependent HDM. (**b**) Exposure to octyl-α-KG (or vehicle control) in cells grown under hypoxia (1% O_2_) led to an increase in HIF1α hydroxylation (Pro-402) and consequent decrease in total HIF1α levels. Densitometric quantification is shown at the bottom of the western blots. (**c**) Octyl-α-KG significantly increased the abundance of 5hmC marks in the DNA (*P*=0.0001, two-tailed Student’s *t*-test, left panel) and decreased that of 5mC marks (global DNA methylation) (*P*=0.0001, two-sided Student’s *t*-test, right panel), in a dose-dependent fashion. The data shown in **c** represent the mean and s.d. of an assay performed in triplicate. Data shown in **a**–**c** were confirmed with at least one independent biological replicate. (**d**) HEK-293 cells stably expressing WT D2HGDH were exposed to 1 mM of dimethyloxalylglycine (DMOG) for 6 h, and the methylation levels of H3 lysines verified by western blot. Exposure to DMOG restored K4me3 and K9me2 in D2HGDH-WT cells to the levels found in MSCV control. (**e**) Hypoxia (1% O_2_ for 18 h) increases HIF1α hydroxylation (Pro-402) and decreases its stability and activity, defined by GLUT1 expression, in cells expressing WT D2HGDH when compared with isogenic control cells (MSCV); exposure to DMOG fully countered the effects of WT D2HGDH on HIF1α and GLUT1. (**f**) D2HGDH-WT expressing cells display significantly higher and low abundance of 5hmC (left panel) and 5mC (right panel) marks, respectively, than its isogenic controls expressing an empty MSCV vector (*P*=0.0008 and *P*=0.0016 ANOVA). Exposure to DMOG (1 mM for 6 h) reversed the effects of WT D2HGDH back to the MSCV baseline. Experiments shown in **d** and **e** were repeated twice, the data in **f** represent the mean and s.d. of a representative experiment (from two biological replicates) performed in triplicate.

To confirm the role of α-KG in our system, we used the cell-permeable octyl-α-KG and fully recapitulated the effects of D2HGDH-WT expression towards histone/DNA methylation and HIF1α hydroxylation (Fig. 3a–c). Consistent with these data, exposure to DMOG abrogated the cellular effects of WT D2HGDH (Fig. 3d–f), probably via competitive inhibiting of α-KG, as suggested earlier in IDH1/2 mutant models^27^. We concluded that the D2HGDH mutants encode an inactive protein, and that subtle variations on the expression the WT enzyme increases α-KG production, and thus influence histone and DNA methylation and HIF1α hydroxylation in a manner that is compatible with higher activity of α-KG-dependent dioxygenases.

### D2HGDH haploinsufficiency in lymphoma

**Figure 4:**
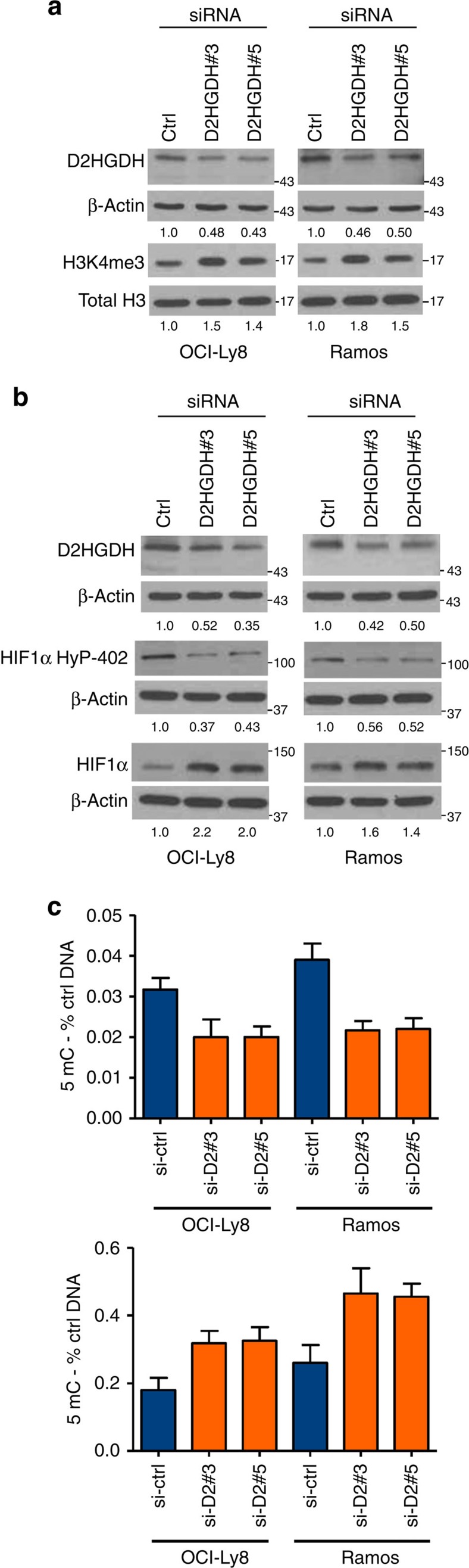
Partial knockdown of D2HGDH in B-cell lymphoma cell lines significantly modifies histone/DNA methylation and HIF1α hydroxylation. (**a**) SiRNA-mediated partial knockdown of D2HGDH with two targeting oligonucleotides increased the methylation levels of H3K4me3 in comparison with cells transfected with a control siRNA. (**b**) Under hypoxia (1% O_2_, 16 h), B lymphoma cells with partial suppression of D2HGDH expression displayed lower HIF1α hydroxylation and consequent stabilization of total HIF1α. In **a** and **b**, the extent of D2HGDH suppression is shown by western blotting and densitometry quantifies all relevant changes. (**c**) The levels of 5hmC and 5mC (top and bottom panels) were significantly lower and higher, respectively, in cells with a D2HGDH KD when compared with their isogenic controls (*P*<0.0001, ANOVA, *P*<0.05 Bonferroni’s multiple comparison test for si-ctrl versus si-D2#3 or si-D2#5). The 5hmC and 5mC measurements shown are the mean and s.d. of three data points derived from three biological replicates. The transient knockdown of D2HGDH in these cell lines was repeated three times.

As the D2HGDH variants identified are heterozygous, and a dominant negative effect is not obvious (compare the nearly indistinguishable measurements of empty vector versus mutant-expressing isogenic cells in multiple assays, (Fig. 2 and Supplementary Figs 4 and 7)), we used an siRNA strategy to partially knock down WT D2HGDH in two B-cell lymphoma cell lines and hence determine whether a hemizygous loss of D2HGDH would be of consequence. In these assays, reducing D2HGDH protein expression by approximately half promptly increased histone methylation, decreased HIF1α hydroxylation and significantly elevated or reduced the abundance of 5hmC and 5mC DNA marks, respectively (Fig. 4a–c). We expanded these observations to an HEK-293 model, and showed that transient or stable KD of D2HGDH modified the readout of function of multiple α-KG-dependent dioxygenases, in association with a decrease and increase in α-KG and D2-HG levels, respectively, while again no changes were found in L2-HG levels (Supplementary Fig. 8). We concluded that partial loss of D2HGDH has metabolic and cellular consequences, supporting a haploinsufficiency model for tumours with heterozygous inactivating mutation of this gene.

### D2HGDH induces mitochondrial IDH activity

D2-HG is a low-abundance metabolite. Therefore, it was necessary to determine whether D2HGDH’s contribution to the cellular pool of α-KG derived exclusively from the oxidation of D2-HG, or if a larger metabolic adaptation was present to explain its ability to meaningfully influence the cellular concentrations of α-KG.

First, we traced the intermediary metabolism with [U-^13C^]glutamine in models of stable D2HGDH-WT and mutant expression, as well as of transient siRNA-mediated KD. The resulting labelling patterns did not reveal any major changes in the way that glutamine carbon is distributed into pools of metabolites along the glutamine degradation pathway, including glutamate, αKG and other TCA cycle metabolites. Specifically, carbon from glutamine was transferred through the glutamate and α-KG pools into the TCA cycle such that there was no difference in the fractional contribution of glutamine to these metabolites in cells expressing WT or mutant D2HGDH. Nonetheless, in these same samples, we confirmed the higher abundance of α-KG in cells expressing D2HGDH WT when compared with the empty vector or mutant enzyme-expressing cells (Supplementary Table 3).

**Figure 5:**
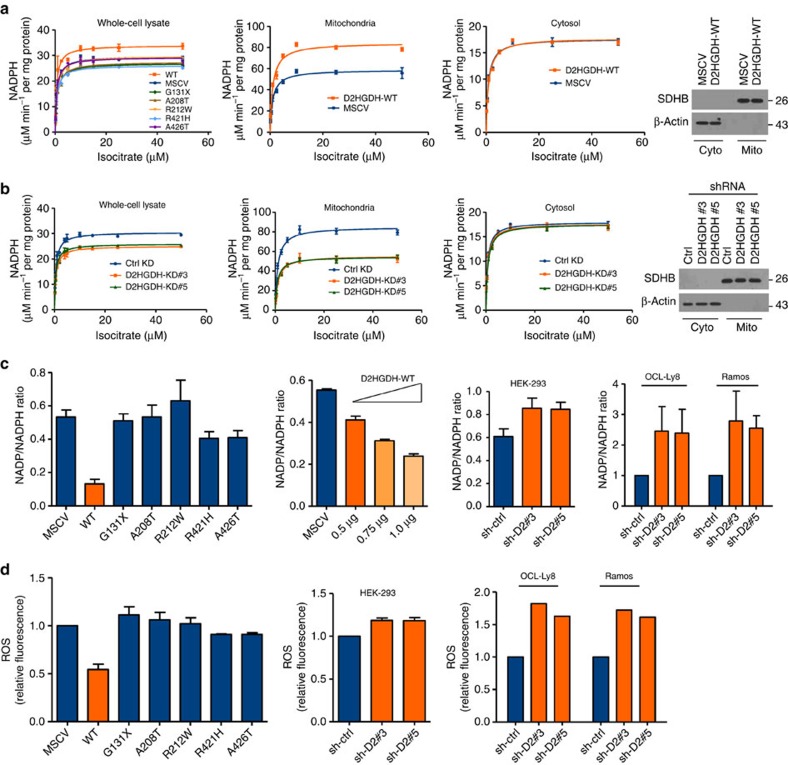
D2HGDH expression influences mitochondrial IDH activity and the cellular redox state. (**a**) Left panel: HEK-293 cells stably expressing WT D2HGDH displayed a significantly higher *V*_max_ for IDH than those expressing mutant D2HGDH or an empty vector (*P*<0.0001, ANOVA). Middle panels: subcellular fractionation demonstrates that D2HGDH increases the *V*_max_ for IDH in the mitochondria, but not in the cytosol; western blots at the right confirm the efficacy of the subcellular separation. (**b**) Left panel: knockdown of D2HGDH significantly decreased the *V*_max_ for IDH (*P*<0.0001, ANOVA). Middle panels: subcellular fractionation demonstrates that D2HGDH levels influences exclusively the *V*_max_ for mitochondrial IDH. Western blots at the right confirm the purity of the subcellular fractions. The data are shown as a nonlinear regression (curve fit) and each panel represents mean±s.d. of an assay performed in triplicate. Together with the data shown in Supplementary Fig. 9, the effects of D2HGDH on IDH activity were confirmed in three independent models of D2HGDH KD and two models of D2HGDH ectopic expression. The enzyme kinetics was calculated with the Michaelis–Menten equation. (**c**) Left: expression of WT D2HGDH significantly lowered NADP/NADPH ratio in comparison with isogenic cells expressing mutant D2HGDH or an empty vector (*P*=0.017, ANOVA), data shown are mean±s.d. from two biological replicates. Middle: transient expression of WT D2HGDH significantly lowered the NADP/NADPH ratio (*P*<0.0001, ANOVA); data shown are mean±s.d. of an assay performed in triplicate. Right panels: Stable (HEK-293) or transient (OCI-Ly8 and Ramos) knockdown of D2HGDH significantly increased NADP/NADPH ratio (*P*=0.01 and *P*=0.003, ANOVA, for HEK-293 or OCI-Ly8 and Ramos, respectively). Data shown are mean±s.d. of three biological replicates. (**d**) Left: cells expressing WT D2HGDH displayed significantly lower ROS levels than the isogenic models of mutant D2HGDH or empty-MSCV (*P*=0.0002, ANOVA); data shown are mean±s.d. from two biological replicates. Stable (middle) or transient (right) KD of D2HGDH in three distinct cell models significantly increased ROS levels (*P*=0.007, ANOVA, for all comparisons). Data shown at the middle are from two biological replicates; data on the right are from a single assay. In all instances, the Bonferroni’s multiple comparison *post hoc* test yielded a *P*<0.05).

As IDH is a major contributor to the cellular pools of α-KG, we next considered the possibility that D2HGDH may influence IDH activity. To address this possibility, IDH activity was determined by measuring the increase of absorbance at 340 nm generated by the conversion of NADP to NADPH, in the presence of increasing concentrations of isocitrate; in these experiments, controls included assay solution lacking isocitrate or reactions performed in the absence of protein lysate. These assays were initially performed in whole-cell lysates, thus reflecting the combined IDH1 and IDH2 activities. These analyses revealed a consistent effect of D2HGDH on IDH function—cells expressing WT D2HGDH displayed higher IDH activity than those expressing an empty vector or any of the mutant enzymes, while KD of D2HGDH markedly suppressed IDH activity (Fig. 5a,b and Supplementary Tables 4 and 5). To better characterize this observation, we isolated cytosolic and mitochondrial fractions and repeated the IDH activity assays. D2HGDH expression influenced IDH activity exclusively in the mitochondria, presumably reflecting IDH2 function (Fig. 5a,b and Supplementary Tables 4 and 6). We also showed that the influence of WT D2HGDH on IDH activity was ‘dose-dependent’ and readily detected in our model of transient physiologic increase in D2HGDH expression, or partial KD of D2HGDH in B-lymphoma cell lines (Supplementary Fig. 9, Supplementary Tables 5 and 6).

We then considered that if these enzymatic assays were capturing a relevant cellular event, then the NADP/NAPDH ratio and consequently reactive oxygen species (ROS) abundance may also be modified by D2HGDH expression and mutational status. In agreement with this concept, we found a significantly lower NADP/NADPH ratio in cells expressing D2HGDH WT compared with their isogenic counterparts expressing the mutant enzyme (Fig. 5c); no significant changes in NAD/NADH ratio was noted (not shown), thus possibly excluding an effect of D2HGDH towards mitochondrial IDH3. These findings were confirmed upon transient expression of physiologic levels of WT D2HGDH, whereas KD of D2HGDH in multiple cell models resulted in a higher NADP/NAPDH ratio (Fig. 5c). Accordingly, cells expressing mutant D2HGDH or with a specific KD of this gene, showed significantly higher ROS levels than their isogenic counterparts expressing the WT enzyme or a siRNA control (Fig. 5d). We concluded that D2HGDH modulates the cellular pool of α-KG at least in part by influencing IDH activity in the mitochondria, and we suggest that the accumulation of ROS may be part of the pathogenesis of disorders associated with D2HGDH loss.

### IDH2 rescues the cellular effects of D2HGDH

**Figure 6:**
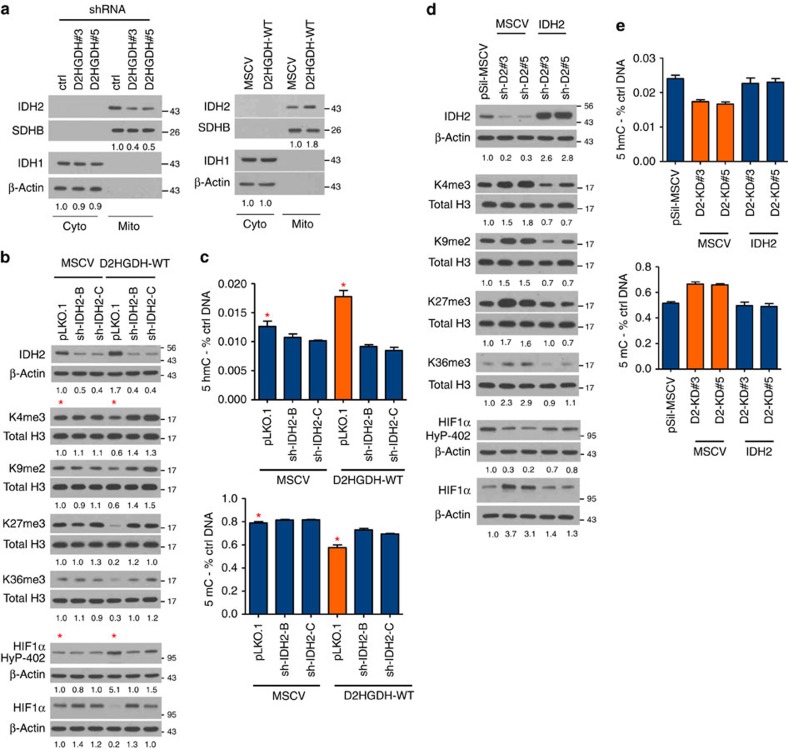
IDH2 mediates D2HGDH effects on histone and DNA methylation and HIF1α hydroxylation. (**a**) Western blot analysis of IDH1 and IDH2 in subcellular fractions of D2HGDH models (knockdown, left panel; ectopic expression, right panel) shows modulation of IDH2 levels. Densitometric quantification is shown at the bottom. (**b**) Top—western blot analysis of IDH2 KD cells expressing an empty-MSCV vector or WT D2HGDH. Middle—Expression of WT D2HGDH decreased H3K methylation (compare lanes marked with a red star); IDH2 KD restored the H3K methylation levels in these cells. Bottom—expression of WT D2HGDH increased HIF1α hydroxylation and decreased total HIF1α levels (compare lanes marked with a red star); IDH2 KD reversed the increase in HIF1α hydroxylation (and decrease in total HIF1α) associated with expression of D2HGDH WT. (**c**) Cells expressing D2HGDH-WT (and an empty pLKO.1) displayed a significantly higher abundance of 5hmC marks (top) and a concomitant decrease in global DNA methylation (botton) than MSCV-pLKO.1 controls (marked by red star; *P*<0.001 two-tailed, Student’s *t*-test). IDH2 KD significantly lowered or increased the levels of 5hmC and 5mC marks, respectively, in D2HGDH-WT cells (*P*<0.0001, ANOVA, *P*<0.001 Bonferroni’s multiple comparison test), with a more modest change in MSCV-expressing controls. These data are mean and s.d. of an assay performed in triplicate, which was confirmed in an independent biological replicate. The data from the WBs were confirmed in two to three biological replicates. (**d**) Top—western blot analysis of IDH2 ectopic expression in D2HGDH KD cells. Middle—D2HGDH KD elevated H3K methylation (compare the three first lanes) and this change was abrogated by expression of IDH2. Bottom—D2HGDH KD decreased HIF1α hydroxylation and increased its total levels (compare the three first lanes); these changes were absent in D2HGDH KD cells ectopically expressing IDH2. (**e**) D2HGDH KD decreased the abundance of 5hmC marks (top) and increased global DNA methylation 5mC (bottom; <0.0001, ANOVA); expression of IDH2 restored these values to that of control (pSIL-MSCV) isogenic cells. These data are mean and s.d. of an assay performed in triplicate, confirmed in a biological replicate. The western blot data shown in **d** were confirmed in biological replicates.

We next explored how D2HGDH may influence IDH activity. To be certain that the cytosolic and mitochondrial fractions shown above were reflecting IDH1 and IDH2 function, respectively, we used western blot to detect these proteins in their subcellular compartments. Indeed, IDH1 was found exclusively in the cytosol and IDH2 in the mitochondria (Fig. 6a). Remarkably, these assays also revealed that D2HGDH KD cells expressed lower IDH2 levels, while cells ectopically expressing D2HGDH had increased IDH2 abundance; D2HGDH did not influence IDH1 expression (Fig. 6a). We confirmed these observations in multiple cell models, including the detection of a progressive upregulation of IDH2 upon transient expression of increasing amounts of D2HGDH (Supplementary Fig. 10). Further, we found that the D2HGDH-dependent IDH2 modulation occurs at transcriptional level (Supplementary Fig. 10). Next, we attempted to determine whether the α-KG generated by the D2HGDH-mediated oxidation of D2-HG in the mitochondria could be involved in the regulation of IDH2 expression. To that end, we used the cell-permeable octyl-α-KG and after brief exposure (6 h) we measured its effects on IDH expression. Remarkably, this synthetic α-KG readily induced IDH2, but not IDH1, at both messenger RNA and protein levels (Supplementary Fig. 10), thus preliminarily suggesting a mechanism by which D2HGDH regulates IDH2 expression.

We postulated that if the disruption of IDH2 expression/activity accounted for the D2HGDH-associated effects on α-KG-dependent dioxygenases, then we should be able to ‘rescue’ these cells with genetic modulation of IDH2 expression. To address this possibility, we created a series of dual genetic models: we stably knocked down IDH2 in cells expressing WT D2HGDH, and we ectopically expressed IDH2 in cells with D2HGDH KD. In all the models, we then quantified histone methylation, 5hmC and 5mC abundance and HIF1α hydroxylation. IDH2 knockdown fully countered the effects of WT D2HGDH, with minimal influence on MSCV control cells, whereas ectopic expression of IDH2 abrogated the effects associated with D2HGDH loss (Fig. 6b–e). Importantly, modulation of IDH2 levels did not change D2HGDH expression (Supplementary Fig. 11). We concluded that in our models deregulated IDH2 expression mediates at least part of the D2HGDH effects towards α-KG-dependent dioxygenases.

### D2HGDH status influences the epigenetic remodelling of DLBCL

**Figure 7:**
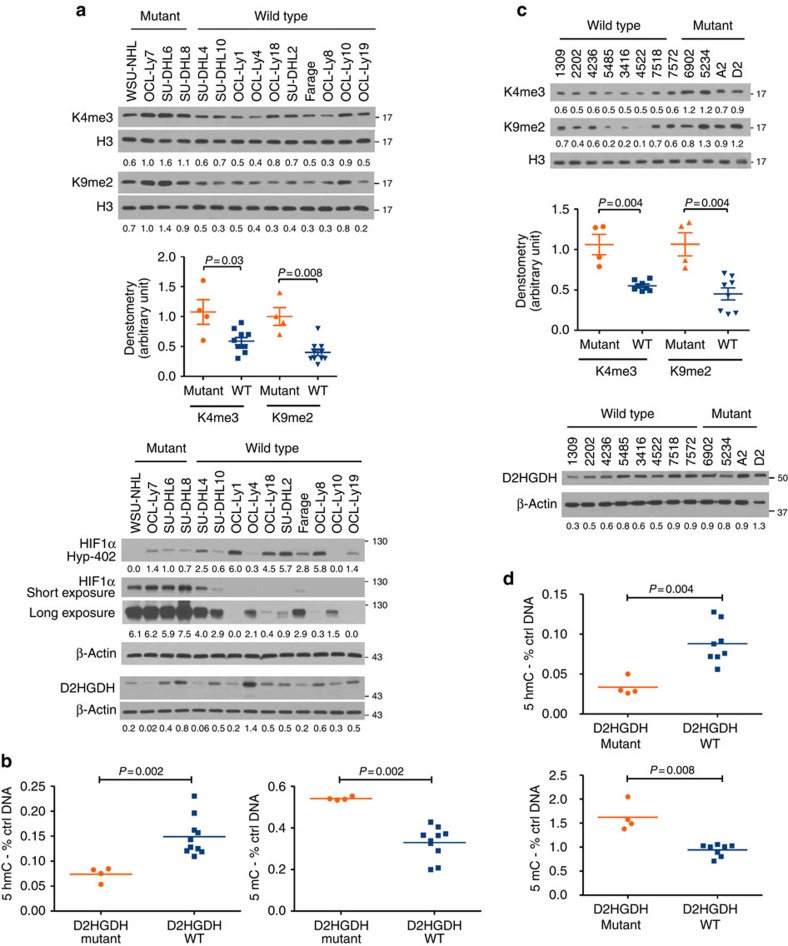
Cellular effects of *D2HGDH* mutations in DLBCL. (**a**) Methylation of H3 lysine residues and HIF1α hydroxylation/total levels (under hypoxic conditions) were determined by western blot in 14 DLBCL cell lines. H3K4 and K9 methylation were higher, while HIF1α hydroxylation (Hy-HIF1α) was lower (and its total levels consequently higher) in D2HGDH-mutant cell lines when compared with those expressing the WT enzyme. Densitometric quantifications are shown at the bottom of the western blots, and for H3K4me3 and H3K9me2 also in graphic display (mean and s.e.m., Mann–Whitney test). The WB at the bottom displays the expression of D2HGDH across these cell lines. (**b**) The levels of 5hmC (left) and the 5mC (right) were significantly lower and higher, respectively, in DLBCL cell lines expressing a mutant D2HGDH gene than in the WT cells (*P*=0.002, two-tailed Mann–Whitney test). The data shown are mean of 3 data points for each cell line (four mutant and ten WT) derived from three independent biological replicates. (**c**) Western blot analysis of 12 primary DLBCLs (four mutant and eight D2HGDH WT) shows higher H3K4 and H3K9 methylation in mutant lymphomas. Densitometric quantification of H3K4me3 and H3K9me2 (normalized by total H3) is shown below the blots and in graphic display (mean and s.e.m., Mann–Whitney test). The WB at the bottom shows the expression of D2HGDH across these biopsies. (**d**) The levels of 5hmC (top) and 5Mc marks (bottom) were significantly lower and higher, respectively, in DLBCLs expressing a mutant D2HGDH gene than in the WT tumours (*P*=0.004 or 0.008, two-tailed Mann–Whitney test). The data shown are mean of three independent measurements for each tumour.

Mutations in chromatin-modifying enzymes are frequent in DLBCL^21^, suggesting that aberrant epigenetic remodelling is an integral component of lymphoma biology. Our data indicate that loss of D2HGDH, by disrupting the activity of α-KG-dependent dioxygenases, may also contribute to these pathogenic epigenetic changes. To address this possibility, we first investigated a panel of parental DLBCL cell lines and primary tumours expressing either mutant or WT D2HGDH. We consistently found that the D2HGDH-mutant DLBCL cell lines (*n*=4) displayed higher H3K methylation, lower HIF1α hydroxylation, lower levels of 5hmC and more prominent DNA methylation (Fig. 7a,b) than their WT counterparts (*n*=10). D2HGDH-mutant primary DLBCLs also displayed higher H3K methylation, fewer 5hmC marks and higher global DNA methylation than the D2HGDH-WT biopsies (Fig. 7c,d). Importantly, although some of the DLBCL cell lines and primary tumours examined harbour mutations in histone methylation regulators (Supplementary Tables 7 and 8), the presence of these abnormalities, appears to not influence the D2HGDH-driven separation in groups of samples with high and low H3K4/K9 methylation levels. To better understand how the D2HGDH status may be driving this dichotomy, we performed three independent LC-MS-based measurements of α-KG and D2-HG levels in DLBCL cell lines mutant or WT for D2HGDH. Although the abundance of these metabolites varied greatly across these samples, when taken as a group we found lower levels of α-KG (but not higher levels of D2-HG) in D2HGDH-mutant compared with WT samples; this observation correlated well with a trend for lower expression of IDH2 in D2HGDH mutant DLBCL cell lines (Supplementary Fig. 12).

To further isolate the role of D2HGDH in these processes, we generated another genetic model by ectopically expressing WT-D2HGDH in each of the four D2HGDH mutant cell lines, and measured its consequences on DNA and histone methylation as well as HIF1α-hyroxylation. Expression of the WT enzyme in these mutant cells readily modified H3K methylation, HIF1α hydroxylation and the abundance of 5hmC marks. In agreement with a role for α-KG in this process, these D2HGDH-induced cellular effects were fully reversed by DMOG (Supplementary Fig. 13). Notably, ectopic expression of WT D2HGDH in the mutant DLBCL cell lines also induced IDH2 expression, further linking the activity of these two mitochondrial proteins (Supplementary Fig. 13). We concluded that as a group, DLBCL-harbouring mutant D2HGDH display lower α-KG levels, a trend for lower IDH2 expression, which we suggest accounts for the broad deregulation of dioxygenases.

## Discussion

In this work, we report discoveries with implications to the fields of cancer, metabolism and epigenetic remodelling. We identified and functionally characterized D2HGDH mutations in DLBCL and elucidated a previously unknown mitochondrial cross-talk between D2HGDH and IDH2. These findings point to α-KG as an important metabolite in regulating the plasticity of the epigenome.

The discovery of *D2HGDH* mutations in DLBCL is of relevance because mutation in epigenetic modifiers has recently been shown to be one of the hallmarks of this malignancy^21^. Certainly, the frequency of D2HGDH mutations in DLBCL that we are reporting is low. However, recent evidence suggest that the discovery of cancer genes has not yet reached saturation, and that larger sample sizes will be needed to identify the true mutation frequencies of less frequently targeted sequences^28^. In agreement with this argument, D2HGDH variants were not reported following systematic screens of the DLBCL exome^15^,^16^,^17^,^18^, possibly reflecting the sample size and frequency threshold for mutation calls used in those studies (Supplementary Note). Our data indicate that *D2HGDH* is one of genes that when mutant may promote epigenetic reprograming in DLBCL. Demonstration that D2HGDH-mutant DLBCL cell lines display lower α-KG levels, and that this metabolite markedly influence histone and DNA methylation is consistent with this concept. However, we acknowledge that given the heterogeneity of DLBCL, one cannot exclude the possibility that additional genetic events may contribute to part of the epigenetic differences that we found in the DLBCL cell lines and primary tumours dichotomized by the D2HGDH status. Nonetheless, the role of D2HGDH in driving at least part of this remodelling is supported by multiple isogenic B-cell lymphoma models of loss and gain of D2HGDH function.

Although loss-of-function mutations in *D2HGDH* have long been shown to cause type I D-2-hydroxyglutaric aciduria^25^, ours is the first detailed report of mutation of this mitochondrial enzyme in cancer. Nonetheless, upon reviewing recent systematic genetic screens of various solid tumours and of the related Burkitt lymphoma^29^ we noted the presence of rare somatic D2HGDH mutations with similar features of those that we found in DLBCL, including: targeting of highly conserved residues, primarily missense changes, heterozygous status, clustering in two protein regions and, on occasion, previous description in families with type I D-2-hydroxyglutaric aciduria (Supplementary Table 9). We conclude that rare somatic D2HGDH mutation is a pervasive event in cancer; as mutation frequency is not an absolute determining factor for their relevance^28^, we propose that the final determination as to whether D2HGDH variants contribute to cancer initiation and/or progression awaits the generation of relevant mouse models. Finally, it is important to make a distinction between the reported higher risk of cancer in patients with deficiency of L2HGDH^13^, and the apparent lack of such effects in infants diagnosed with D-2-HGA. Here, it should be noted that D-2-HGA may have a more aggressive clinical course than L-2-HGA^11^,^25^, and as the patients succumb to their disease more rapidly, it may not be possible to determine whether deficiency of D2HGDH can also influence the long-term susceptibility to cancer.

The D2HGDH mutations described in type I D-2-hydroxyglutaric aciduria and in DLBCL are highly clustered. Using structural modelling, we showed that one group of mutants locates close to the point of contact with the enzyme’s co-factor FAD. Disruption of these interactions is likely to affect the enzyme’s activity and can be easily reconciled with a loss-of-function phenotype. The second cluster of mutations mapping the C terminus of the protein is more intriguing. This region does not possess any recognizable structural or functional domain, and yet its consistent disruption in D2HGDH-related conditions suggest that it is important for the enzyme’s activity. We explored the possibility that this region could be a putative dimerization or subcellular localization domain. Although we discovered that D2HGDH can homodimerize, none of the mutations affected this property nor did they change the enzymes’ mitochondrial localization. Complete characterization of the functional domains of D2HGDH is an important future objective.

D2-HG is considered an unwanted byproduct of cellular metabolism with no known physiologic role^9^. It is certain, nonetheless, that cellular accumulation of D2-HG is toxic, as evidenced by the extensive neurologic disease and rapid clinical deterioration of infants with bi-allelic loss of D2HGDH. The enzymatic activity of D2HGDH helps keep the intracellular levels of D2-HG below <0.1 mM and to date this has been considered the primary, if not only, function of this dehydrogenase^9^. However, our data suggest otherwise. We have shown that even modest increases in D2HGDH expression markedly elevated the levels of intracellular α-KG. This observation was at first puzzling for it was unclear how the D2HGDH-dependent interconversion of the nearly undetectable D2-HG into α-KG, could contribute significantly to the mM cellular concentrations of α-KG. We solved this conundrum with an enzymatic examination of IDH activity in subcellular fractions and the demonstration that D2HGDH positively influences the expression and activity of the mitochrondrial IDH2, but not the cytosolic IDH1. Together, these observations challenge the notion that D2HGDH is simply a cellular guardian against the toxic accumulation of D2-HG. Instead, our data suggest that D2HGDH may function a rheostat dialling up and down IDH2 function and, therefore, the intracellular levels of α-KG. We also made inroads in the initial characterization of the mechanism by which D2HGDH positively influences IDH2 expression. We showed that a synthetic cell-permeable α-KG recapitulates the effects of D2HGDH and also induce IDH2 transcription. It still remains to be determined whether α-KG acts directly on the IDH2 promoter or whether it uses intermediates—the rapid (6 h) effects of synthetic α-KG suggests that in this context IDH2 behaves as an immediate early gene and it is transcriptionally induced before any new proteins are synthesized. These data also demonstrate an important difference in the regulation of the related IDH1 and IDH2 proteins. Together, these observations led us to speculate that physiologically, the local (mitochondrial) increment in α-KG levels resulting from the D2HGDH-driven conversion of D2-HG into α-KG induces IDH2 transcription. Important tasks for future studies include characterization of the elements in the IDH2 promoter, and associated transcription factors that may mediate this response. In addition, the demonstration that discrete increments in D2HGDH levels influence α-KG abundance and the activity of multiple epigenetic modifiers make the identification of the signals and regulatory elements that drive D2HGDH expression a high priority for additional investigations.

One notable distinction between our data and those derived from somatic gain-of-function IDH1/2 mutation or bi-allelic loss of D2HGDH in the germline, is that the principal metabolic consequence of the heterozygous D2HGDH mutations found in DLBCL is deficiency of α-KG, not massive accumulation of D2-HG. It can be speculated that considering the very low intracellular levels D2-HG even when D2HGDH is functioning at half capacity (that is, heterozygous loss), it still can prevent D2-HG accumulation. However, because D2HGDH uses IDH2 to modify the total pool of intracellular α-KG, we suggest that the modest mitochondrial change in α-KG abundance derived from a haploinsufficient D2HGDH can have large repercussions. Irrespective of the precise underpinnings, our data suggest that subtle D2HGDH-mediated changes in α-KG levels may regulate the activity of dioxygenases and rewrite several epigenetic marks. Indeed, we show that discrete changes in D2HGDH levels or a brief exposure to synthetic α-KG or its competitive inhibitor DMOG influence histone and DNA methylation, as well as HIF1α hydroxylation. These data bring to the fore the relevance of α-KG in multiple cellular functions, as recently highlighted in a nematode model and in embryonic stem cells^22^,^23^. We propose that once better understood at molecular and cellular levels, modulation of α-KG with therapeutic intent may have a role in cancer therapeutics.

In conclusion, by characterizing rare D2HGDH mutations found in DLBCL, we unveiled an unsuspected role for this enzyme in modulating IDH2 expression and activity, and consequently the cellular α-KG pool. In our models, subtle titration of D2HGDH levels had a marked repercussion towards histone and DNA methylation and HIF1α hydroxylation, consistent with a change in the activity of α-KG-dependent dioxygenases. Our findings also indicate that the relevance of D2HGDH to cellular physiology may extend beyond preventing the toxic accumulation of D2-HG, and underscore the need for a tight control of D2HGDH expression as modest variations of its levels can engage an IDH2-α-KG-dioxygenases axis that has the potential for profound effects on cellular regulation by epigenetic remodelling. Strikingly, the finding of IDH2 (but not IDH1) gain-of-function mutations in type II D-2-HGA patients^30^ provides an independent validation of the hitherto unsuspected biological connectedness between D2HGDH and IDH2. Finally, our data may advance the understanding of hereditary conditions that associate with D2HGDH dysfunction.

## Methods

### Primary DLBCL samples and cell lines

Frozen biopsies from 72 untreated DLBCL patients were obtained from our local tumour bank, Department of Pathology, University of Texas Health Science Center at San Antonio. The clinical, pathological and molecular features of this tumour collection were described previously^31^. Tissue was available from an additional 48 DLBCL cases diagnosed at the Division of Hematology, Medical University of Graz, Austria. Protein was also isolated from 12 primary DLBCLs (four D2HGDH-mutant and eight D2HGDH-WT); the selection of the D2HGDH-WT or mutant for further analyses was based on the availability of frozen material and their clinical/ phenotypical similarities. The use of these samples was approved by the Review Boards of each Institution. The DLBCL cell lines SU-DHL2, SU-DHL4, SU-DHL6, SU-DHL8, SU-DHL10, OCI-Ly1, OCI-Ly4, OCI-Ly7, OCI-Ly8, OCI-Ly10, OCI-Ly18, OCI-Ly19, Farage and WSU-NHL were cultured at 37 °C in 5% CO_2_ in RPMI-1640 medium (Invitrogen) containing 10% (vol/vol) FBS (or 20% FBS for SU-DHL2 and OCI-Ly10 cultures), whereas HEK-293 cells were maintained in Dulbecco’s modified Eagle media (DMEM; Mediatech) with 10% FBS, as we described^32^. All D2HGDH-mutant DLBCL cell lines are of the GCB molecular subtype, thus we initially selected five D2HGDH-WT cell lines also of the GCB subtype (SU-DHL4, SU-DHL10, OCI-Ly1, OCI-Ly4, OCI-Ly18) for comparison^15^. This group was next extended to include five additional D2HGDH-WT DLBCL cell lines (SU-DHL2, Farage, OCI-Ly8, OCI-Ly10, OCI-Ly19) also characterized by WT configuration of multiple chromatin modifier genes (MLL2, MLL3, MLL4, MLL5 and EZH2; Supplementary Table 7) that are relevant in the present context. The identity of the mutant DLBCL cell lines was confirmed by VNTR analysis and verified online at the DSMZ cell bank (http://www.dsmz.de) and tested for Mycoplasma contamination before this project started. All the cell lines were pre-existent in our group and were earlier obtained from ATCC or DSMZ cell bank or from Margaret Shipp (OCI-Ly10) (Dana-Farber Cancer Institute), Laura Pasqualucci (SU-DHL2; Columbia University) or Sandeep Dave (HBL-1, U2932; Duke University).

### DNA isolation and sequencing

High-molecular weight DNA from the primary DLBCL biopsies (*n*=120) and DLBCL cell lines (*n*=29) was isolated using Gentra Puregene DNA purification kits (Qiagen). For all 149 samples, the nine coding exons (and exon/intron junctions) of D2HGDH were amplified; the 10 coding exons (and exon/intron junctions) of the L2HGDH gene were PCR amplified from 68 samples; the exon 4 of IDH1 and IDH2 were amplified in 80 samples (primers sequence in Supplementary Table 10). The resulting 2,172 amplicons were sequenced directly from both strands, and compared with the reference germline sequences, using the Mutation Surveyor Version 2.41 (Softgenetics), as we described^33^. All variants were verified at the human dbSNP Database (Build 132) and Ensembl Database. All mutations were confirmed on independent PCR products, and the somatic nature of the mutation was documented in two cases with matched normal DNA (bone marrow) available. The identity of the mutant DLBCL cell lines was confirmed by VNTR analysis and verified online at the DSMZ cell bank (http://www.dsmz.de). The exons 4, 5, 8 of D2HGDH were also PCR amplified and sequenced in 190 control alleles and exon 9 in 290. For the mutant samples with available RNA (cell lines WSU-NHL, SU-DHL6 and SU-DHL8, and primary biopsy #6902), PCR with reverse transcription (RT–PCR) was used to amplify a fragment spanning multiple exons and encompassing the variant nucleotide. Subsequent sequencing (or cloning followed by sequencing) of these fragments was implemented for quantification of the relative contribution of the mutant and WT alleles to D2HGDH expression.

### Copy-number analysis within the D2HGDH locus

To detect copy-number variations within the D2HGDH gene, we used MLPA and real-time semi-qPCR. Exons 2, 6, 7, 9 and 10 were analysed with the SALSA P107 MLPA kit (MRC-Holland), according to manufacturer’s instructions. The copy number of exons 3, 4, 5, 8 and of a region 4 Mbp upstream to the gene’s transcription start site was determined by relative quantification using qPCR. Tumour samples (*n*= 50, Supplementary Table 2) were compared with a commercially available control DNA (Promega, Madison) and normalized by a control PCR product on chromosome 22. Relative abundance (copy-number variation) was determined by the 2^−ΔΔCT^ method^31^.

### Structure prediction

Sequence alignment of D2HGDH amino acid sequence against the NCBI database identified D2HGDH as containing a conserved FAD binding domain. Subsequent searches of the structural database (RSCB Protein Data Bank, Release 10 January 2013) using the protein threading algorithm HHPRED^34^ identified a number of flavoproteins with E (expect) values of 10^−40^ or lower, and in turn near 100% probability that these are representative of the overall fold of human D2HGDH. The best scoring structure, a putative dehydrogenase from Rhodopseudomonas palustris (PDB code 3pm9, Joint Center for Structural Genomics, unpublished), had an E-value of 3.2 × 10^−80^. To provide a structure-based explanation for the DLBCL-associated mutations, as well as the mutations previously reported in patients with D-2-hydroxyglutaric aciduria^25^, a homology model for D2HGDH was generated using the programme SwissModel^35^ with chain A from 3pm9 as the template structure and the amino-acid alignment derived from the HHPRED analysis above. The structures shown in Fig. 1c were generated using the programme UCSF Chimera^36^.

### Generation of genetic D2HGDH models

A sequence-verified WT D2HGDH cDNA encompassing its complete coding region was cloned into the MSCV-eGFP vector. Site-direct mutagenesis was introduced by PCR, and all mutants sequence verified. Retrovirus production and transduction in HEK-293, SU-DHL6, SU-DHL8, WSU-NHL and OCI-Ly7 cells was performed as we described^37^ and enriched GFP-positive populations (>90% purity) obtained by fluorescence activated cell sorting (FACS). Stable ectopic expression of D2HGDH was confirmed by western blot and real-time RT–PCR. Electroporation or lipid-based transfections of the same constructs were used to generate models of transient expression of increasing (physiologic) amounts of D2HGDH (WT or mutant) in HEK-293 cells.

### RNAi-based D2HGDH suppression

Transient KD of *D2HGDH* (siRNA) in HEK-293, Ly8 and RAMOS cell lines (WT for the *D2HGDH* gene) was achieved by lipid-based transfection of two independent oligonucleotides specific to this gene; these same sequences were also cloned into the pSilencer vector, as we described^38^, and stable D2HGDH shRNA cells established by puromycin selection. Efficacy of the knockdown was determined by western blot.

### Molecular biology and homodimerization assays

A WT D2HGDH cDNA was PCR-amplified and subcloned in-frame with HA (amino (N) terminus, pHM6 plasmid) or FLAG (C terminus, p3xFLAG-CMV plasmid) tags. The missense D2HGDH mutants (A208T, R212W, R421H and A426T) were PCR-amplified and cloned in-frame with FLAG at the C terminus, whereas the truncating G131X mutant was fused to an N terminus HA tag. All the constructs were sequence verified. Subsequently, pairs of HA and FLAG-tagged D2HGDH constructs (HA-WT+WT-FLAG; HA-G131X+WT-FLAG; HA-WT+A208T-FLAG; HA-WT+R212W-FLAG; HA-WT+R421H-FLAG; HA-WT+A426T-FLAG) were co-transfected in HEK-293 cells, harvested at 48 h post transfection, immunoprecipitated (IP) overnight at 4 ^o^C with anti-FLAG antibody (mouse monoclonal M2, Sigma) or anti-HA antibody (goat polyclonal, Bethyl Laboratories), and analysed with immunoblots for the pull-down HA-D2HGDH and D2HGDH-FLAG, respectively. Controls included the co-transfections with empty HA or FLAG vectors, and IPs with mouse Ig and goat serum. Equal expression of HA and FLAG fusions in the input protein was determined by western blotting. The expression of the D2HGDH fusion proteins was also determined in non-denaturing conditions. In those assays, the protein lysates of the multiple co-transfection indicated above were subjected to electrophoresis with classical denaturing protocol side-by-side with a non-denaturing setting (sample buffer lacking SDS and 2-mercaptoethanol) and immunoblotted with anti-FLAG (1:2,000), anti-HA (1:1,000)and anti-D2HGDH (1:1,000) antibodies.

### IDH2 ectopic expression and knockdown

To generate the IDH2 construct, the gene’s complete coding sequence was PCR amplified from a pOTB7-IDH2 clone (IMAGE:2959540, Invitrogen), sequence verified and subcloned into MSCV. Next, HEK-293 cells stably expressing two shRNA constructs directed at D2HGDH (sh#3 and sh#5) or a sh-ctrl vector, were transduced with empty MSCV or MSCV-IDH2 viruses, and highly purified cell populations obtained by cell sorting. Two previously described shRNA pLKO.1-puromycin constructs directed at IDH2 ref. 39 or shRNA-pLKO.1 control were transduced into HEK-293 cells expressing and empty MSCV-vector or MSCV-D2HGDH, and stable IDH2 KD populations were obtained with puromycin selection. In various models, IDH2 expression was also determined at messenger RNA level using real-time RT–PCR.

### Protein isolation and western blots

For all the experiments, cell lines were grown overnight in 5% FBS media before protein isolation, carried out in 2% SDS, 4% glycerol, 0.04 M Tris-HCL pH=6.8 and 2 mM 2-mercaptoethanol. For HIF1α (hydroxylation and total levels) analysis, cells were grown for 16 h in 1% O_2_ in a hypoxia chamber (Invivo2 200, Ruskinn) and protein harvested inside the chamber. In other assays, a 6-h exposure to 1 mM dimethyloxalylglycine (DMOG; Frontier Scientific) or to 0.5 mM and 1 mM of octyl-α-KG (Cayman Chemical) was carried out before protein isolation. Proteins were detected with specific antibodies directed at: D2HGDH (Proteintech, #13895-1-AP), H3K4me3, H3K9me2, H3K27me3, H3K36me3, H3K79me2 and histone H3 (all from Cell Signaling Technology), HIF1α (BD Biosciences, #61095), HIF1α-hydroxyproline (Pro-402; Millipore, #07-1585), GLUT1 (Novus Biologicals, # NB300-666), IDH1 (Cell Signaling Technology, #8137), IDH2 (Abcam, #ab55271), β-actin (Sigma-Aldrich, #A2228). For examination of GLUT1, the samples were not heat-denatured before gel loading. All PVDF membranes were stripped with OneMinute Western Blot Stripping Buffer (GM Biosciences, #GM6001) and re-probed with histone H3 and β-actin antibodies for loading control. Supportive information on the antibodies utilized is detailed in Supplementary Table 11. All the antibodies were used at 1:1,000 dilution, except for and β-actin (1:20,000). See uncropped western blots in Supplementary Fig. 14.

### IDH activity assay

The activity of isocitrate dehydrogenase was determined by measuring the increase of absorbance at 340 nm generated by the conversion of NADP to NADPH at 25 ^o^C. These assays were performed in total cell lysate, as well as in cytosol and mitochondrial fractions. The standard assay solution (1 ml) contained 2 mM MnCl2, 0.25 mM NADP+ and DL-isocitrate concentrations ranging from 0.5 to 100 μM (DL-isocitric acid, trisodium sate hydrate, 95%, ACROS), as described^40^. Controls included assay solution lacking isocitrate or reactions performed in the absence of protein lysate (in both the cases yielding no measurable enzymatic activity). All the experiments were performed in triplicate. NADPH production was calculated using the NADPH extinction coefficient of 6.22 mM^−1^ cm^−1^. The kinetic parameters (Km and *V*_max_ values) were generated by a plot of enzymatic activities versus substrate concentrations and calculated by the Graph-Pad Prizm Software using the Michaelis–Menten equation.

### Mitochondria fractionation

To separate mitochondria from cytosol, HEK-293 cells were resuspended in lysis buffer containing 200 mM manitol, 68 mM sucrose, 10 mM HEPES-KOH pH 7.4, 1 mM EGTA and protease and phosphatase inhibitors. The cells were disrupted with multiple strokes of a glass douncer, followed by centrifugation and at 600*g* for 10 min. Subsequently, the supernatant was collected and centrifuged at 7,000*g* for 10 min, yielding a pellet representing the mitochondria-enriched fraction. The supernatant (representing the cytosolic fraction) was centrifuged one time (15,700*g* for 10 min) to remove light membranes from the cytosol. To confirm the purity of the fractions, western blotting for SDHB (Invitrogen, #459230; mitochondrial) and β-actin (cytosol) were performed.

### Flow cytometry-based 5hmC measurement

HEK-293 cells stably expressing D2HGDH WT or mutant were washed once with phosphate-buffered saline (PBS), fixed in 1.5% formaldehyde and permeabilized with 0.1% Triton X-100 in PBS for 15 min. Cells were then washed and resuspended in 1% BSA/PBS for 1 h. Anti-5hmC antibody (Active Motif, #39796), was added at 1:500 for 2 h at room temperature (RT), followed by three washes in PBS and incubation with a secondary antibody conjugated with PE (Invitrogen, #P2771MP) at 1:500 for 1 h in the dark. Subsequently, the cells were washed three times in PBS and analysed using a Becton Dickinson LSRII. FACS data were analysed using FACSDiva Software (Becton Dickinson).

### Quantitative 5hmC and 5mC assays

High-molecular weight DNA was obtained from relevant cell lines and primary tumours. For the quantification of 5hmC and 5mC marks, 200 and 100 ng of DNA, respectively, was added to each well (96-well plate format), followed by incubation with primary (anti-5hmC or anti-5mC) and secondary antibodies and developed by colorimetric methods (Epigentek #P-1036 and #P-1034). Abundance of 5hmC and 5mC marks was quantified by absorbance and reported as relative values to the positive controls, methylated polynucleotide containing 20% of 5hmC or 50% 5mC, respectively. In our study, the diametrically opposed levels of 5hmC and 5mC in the same cell model reinforce the high specificity of these measurements, and absence of cross-reactivity between these modifications when defined by these assays. The high concordance between the 5hmC quantifications performed by the FACS or absorbance (Supplementary Fig. 7), further confirm the reliability of this methodology. For the 5hmC and 5mC measurements, at least three independent replicates were performed, and when sufficient DNA was available (for the primary tumours), the assays were done in triplicate.

### Immunofluorescence

HEK-293 cells stably expressing WT or mutant D2HGDH were plated on poly-D-lysine-coated glass coverslips in 24-well tissue culture plates. After 24 h, full media containing 250 nM of the cell-permeable MitoTracker probe (Invitrogen, #M22426) was added and incubated for 20 min. Next, the cells were rinsed in PBS and fixed in 4% paraformaldehyde for 15 min, followed by permeabilization and blocking with 5% horse serum, 0.2% Triton X-100 in PBS. Upon removal of the blocking solution, the primary antibody (anti-D2HGDH, Proteintech, #13895-1-AP) was added at 1:100 in 0.3% Triton X-100/PBS solution and incubated for 1 h at RT. After three washes with PBS, the Cy3-labelled secondary antibody (1:200; Jackson ImmunoResearch,# 711-166-152) was added for 1 h at RT in the dark, followed by three washes in PBS. Coverslips were mounted on glass slides using Vectashield (Vector Laboratories) for imaging. Images were acquired by laser scanning confocal microscope (LSCM) with Olympus FV1000 imaging system^33^. The UPLANAPO × 60 oil objective (1.42NA) was used for all the analyses, and an additional electronic zoom of 3 was applied. Excitation and emission signals were respectively 488 and 500±35 nm for GFP (expressed from a bicistronic construct), 534 and 560–660 nm for Cy3 (D2HGDH), 644 and 665 nm for Cy5 (MitoTracker).

### Metabolite extraction and quantification by LC-MS

HEK-293 cells stably or transiently expressing WT or mutant D2HGDH, HEK-293 with transient knockdown of D2HGDH, as well as 14 DLBCL cell lines (four D2HGDH mutant and 10 WT) were grown overnight in 5% FBS media, gently washed in PBS, collected and flash-frozen in dry ice. For α-KG assays, the cells were extracted with cold 80% aqueous methanol (−80 °C) containing 0.6 nmol [1,2,3,4-^13^C_4_]α-ketoglutaric acid (KG) (Cambridge Isotope Laboratories) and maintained at −80 °C for 1 h, as described^41^. Subsequently, the extracts were centrifuged at 13,800*g* for 10 min and the supernatants were transferred to glass autosampler vials for HPLC-ESI-MS (high performance liquid chromatography-electrospray ionization-mass spectrometry) analysis. The processing of the samples for 2-HG analysis was as above except that 2 nmol [1,2,3,4-^13^C_4_]L-malic acid (Cambridge Isotope Laboratories ) was added as the internal standard and the dried extracts were reacted with diacetyl-L-tartaric anhydride (Sigma-Aldrich), as described^42^. HPLC-ESI-MS analyses were conducted on a Thermo Fisher Q Exactive mass spectrometer with online separation by a Thermo Fisher/Dionex Ultimate 3000 HPLC. HPLC conditions for KG analyses were: column, Luna NH_2_, 3 μm, 2 × 150 mm (Phenomenex); mobile phase A, 5% acetonitrile in water containing 20 mM ammonium acetate and 20 mM ammonium hydroxide, pH 9.45; mobile phase B, acetonitrile; flow rate, 300 μl min^−1^; gradient, 85% B to 1% B over 10 min and held at 1% B for 10 min. The conditions used to separate D-HG from L-HG analyses were: column, Kinetex C18, 2.6 μm, 2.1 × 100 mm (Phenomenex); mobile phase, 1% acetonitrile with 125 mg l^−1^ ammonium formate, pH 3.6; flow rate, 400 μl min^−1^. For both analyses, full scan mass spectra were acquired in the orbitrap using negative ion detection over a range of *m/z* 100–800 at 70,000 resolution (*m/z* 300). Metabolite identification was based on the metabolite accurate mass (±5 p.p.m.) and agreement with the HPLC retention time of authentic standards. Quantification was made by integration of extracted ion chromatograms of each metabolite followed by comparison with the corresponding standard curves. For these analyses, α-KG and D-hydroxyglutaric acid (D-HG) were obtained from Sigma-Aldrich and the HPLC-grade solvents from Fisher Scientific.

The measurements of the D2-HG, L2-HG and α-KG metabolites were also independently performed by liquid chromatography-tandem mass spectrometry^43^,^44^ In brief, 20 μl aliquot of the extracted samples were injected for chromatographic separation on an Agilent Hypersil ODS 4.0 × 250 mm, 5 μm column (Santa Clara, CA). The column oven was set at 30 °C. Solvent A was 125 mg l^−1^ ammonium formate, pH=3.6, solvent B was acetonitrile. The enantiomers were eluted using the programme: 0 to 0.5 min—100% A, 0.5 to 3.5 min—linear gradient to 96.5% A, 3.5 to 25 min—96.5% A and 3.5% B. The flow rate was set at 0.5 ml min^−1^, 50% of the post-column eluate was diverted to waste and the remainder injected into API 3000 triple–quadrupole mass spectrometer equipped with an electrospray ionization source (Applied Biosystems, Foster City, CA) and operating in negative ion mode. Turbo ionspray gas was set at 8 l min^−1^, the temperature at 500 °C and the ionspray voltage at −4,200 V for 2-HG and at −1,700 V for 2-KG. Product ion transitions were monitored at 363.2 to 147.2 for L-2-HG and D-2-HG, 367.1 to 151.1 for D,L-[3,3,4,4-D_4_]-2-hydroxyglutaric acid, 145.15 to 101.1 for α-KG, and 149.2 to 105.2 for 2-ketopentanedioic acid-[D_4_]. Protein concentration was measured using the RC DC Protein Assay (Bio-Rad, Irvine, CA) and on SmartSpec Plus (Bio-Rad). All the metabolite data are shown in Fig. 2, Supplementary Fig. 4, Supplementary Fig. 6, Supplementary Fig. 8, Supplementary Fig. 12 and Supplementary Table 12.

### Mass isotopomer distribution of α-KG

Cells were cultured in DMEM containing [U-^13^C]glutamine and extracts were collected in ice-cold 1:1 methanol:water, subjected to three freeze–thaw cycles and cell debris removed by high-speed centrifugation with cold 80% aqueous methanol^45^. Subsequently, the supernatant was split into two glass tubes and evaporated with blown air at 42 ^o^C. To one of the dried samples, 25 nmoles of unlabelled α-KG was added and the sample was evaporated to dryness again. To all the samples, 50 μl of methoxyamine-hydrochloride in pyridine (2%) was added and the samples were left at RT overnight (∼16 h). The samples were then evaporated again and derivatized in 100 μl Tri-Sil reagent (Thermo) at 42 ^o^C for 1.5 h. All the samples were then injected onto an Agilent 6890N gas chromatograph networked to an Agilent 5975 Mass Selective Detector. Mass isotopomer distribution was determined for α-KG using methods analogous to those used for other TCA cycle intermediates^45^. The abundance of α-KG was calculated on the basis of the fold dilution of enrichment caused by the addition of 25 nmoles unlabelled α-KG.

### Measurement of NADP/NADPH and ROS levels

NADP/NADPH levels were quantified using an assay kit from Abcam (ab65349) and performed according to its instructions. In brief, multiple cell models of D2HGDH ectopic expression or KD were extracted using the manufacturer provided buffer, mixed with NADP cycling solution, with the NADPH developer and absorbance measured at OD450 nm. Total NADP and NADPH concentrations were calculated by correlation to the curve generated with the NADPH standard (NADPH DS). NAD/NADH levels were quantified as above, but using the Abcam assay kit #ab65348. For ROS detection, we performed DCFDA assays (Life Technologies/Molecular Probes #C6827). In brief, the cells were incubated with 10 μM of CM-H_2_DCFDA (5-(and-6)-chloromethyl-2′,7′-dichlorodihydrofluorescein diacetate, acetyl ester) at 37 ^o^C for 10 min. Excess CM-H_2_DCFDA was removed by washing the cells twice in PBS, followed by lysis in 10% NP40 buffer. The homogenates were centrifuged to remove cellular debris. Oxidation of DFCDA to DFC (a measure of ROS generation) was determined using a spectrofluorometer with excitation at 495 nm and emission at 525 nm.

### Statistics

Analyses were performed using a one-way ANOVA, with Bonferroni’s multiple comparison *post hoc* test, and by two-tailed Student’s *t*-test or Mann–Whitney test. Equal variance was calculated with an F-test (*t*-test) or with a Bartlett’s statistics for equal variances (ANOVA, groups with five or more values). *P*<0.05 was considered significant. Data analyses were performed in the Prism software (version 5.02, GraphPad Software Inc) and Excel (Microsoft).

## Additional information

**How to cite this article:** Lin, A.-P. *et al*. D2HGDH regulates alpha-ketoglutarate levels and dioxygenase function by modulating IDH2. *Nat. Commun.* 6:7768 doi: 10.1038/ncomms8768 (2015).

## Supplementary Material

Supplementary Information Supplementary Figures 1-14, Supplementary Tables 1-12, Supplementary Note 1 and Supplementary References
