# Supplementary material for: D2HGDH regulates alpha-ketoglutarate levels and dioxygenase function by modulating IDH2
Source: Nat Commun. 2015 Jul 16;6:7768. doi: 10.1038/ncomms8768 (PMC4515030; doi:10.1038/ncomms8768)
Supplement: Supplementary Information — Supplementary Figures 1-14, Supplementary Tables 1-12, Supplementary Note 1 and Supplementary References [file ncomms8768-s1.pdf]

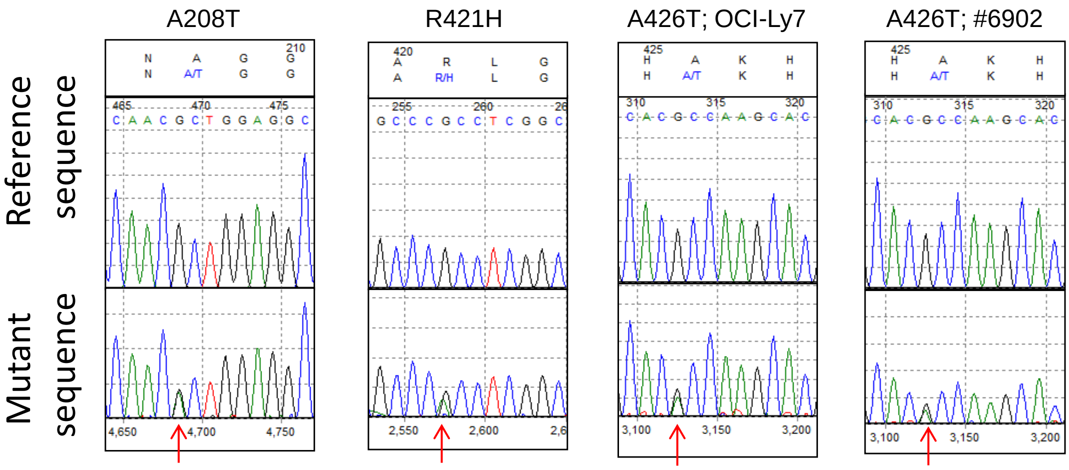


**Supplementary Figure 1. Mutant and WT D2HGDH are co-expressed in DLBCL**. Sequencing tracing of RT-PCR products representing the expression levels of three DLCBL-associated D2HGDH mutations indicate that the mutant and WT alleles are equally expressed. The frame-shift nature of the G131X prevented a clear display of the histogram for that variant. However, cloning and sequencing of those RT-PCR products showed a balanced representation of WT and mutant forms (4 mutant, 6 WT in 10 clones – data not shown). No mRNA was available from tumours harbouring the R212W mutation. The expression of A426T mutation is shown in both a cell line and in a primary DLBCL.

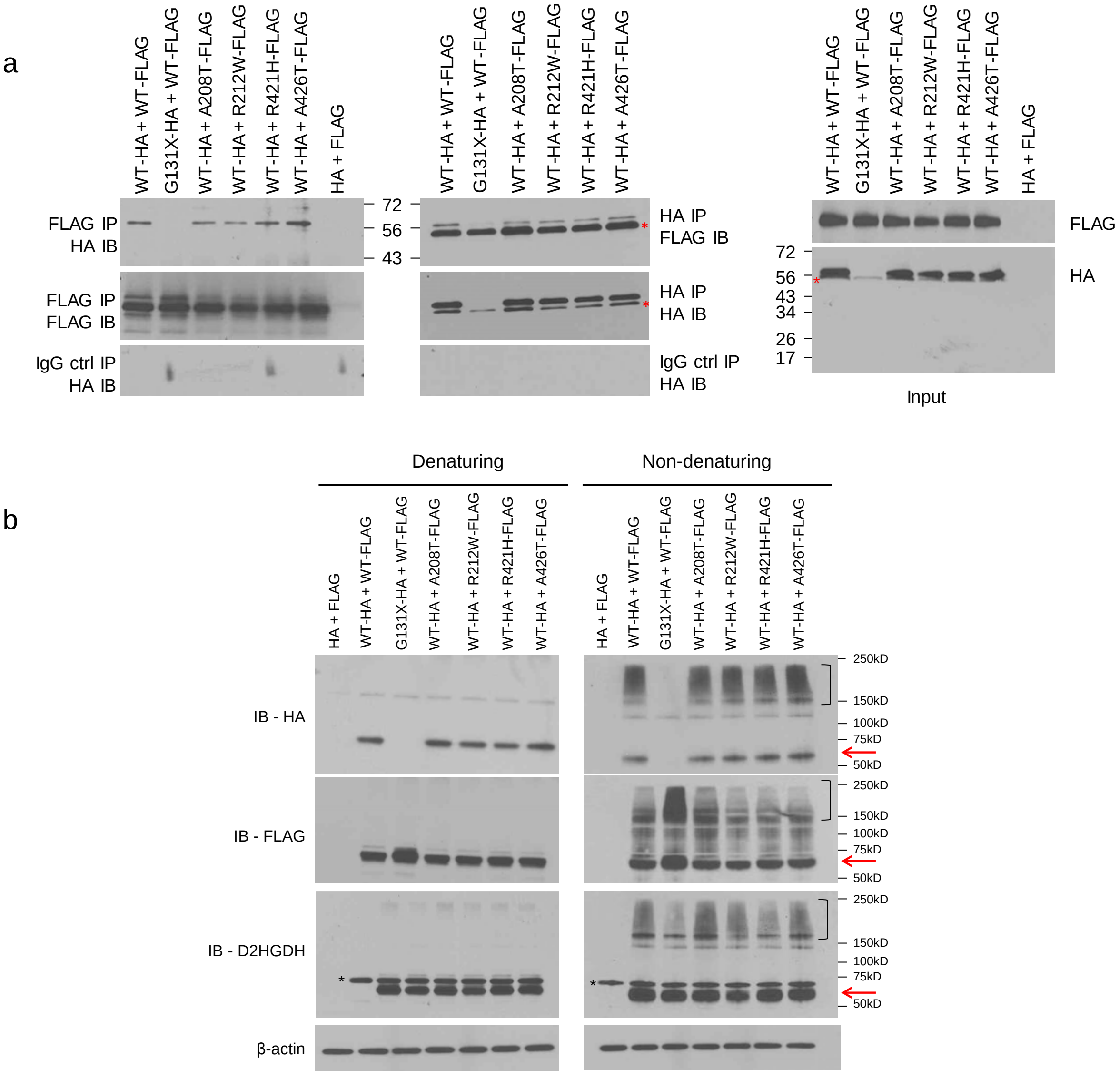


**Supplementary Figure 2. D2HGDH is a self-interacting protein**. **a)** FLAG- or HA-tagged D2HGDH WT and mutant constructs were co-transfected in HEK-293 cells (different combinations of WT and mutant proteins are listed on the top). Immunoprecipitation (IP) of D2HGDH-FLAG pulled-down HA-D2HGDH (left panel - top). Likewise, IP of HA-D2HGDH, brought-down D2HGDH-FLAG (middle panel - top). These data indicate that D2HGDH self-complexes and that the DLBCL-associated mutations do not disrupt this binding. Note that the G131X truncating mutation probably results in an unstable protein (predicted size 17KD), which could not be detected with N-terminus tagging (HA-D2HGDH-G131X – see input blot). The HA-IP/IB yields a non-specific band indicated by an *. Co-transfection of empty-vectors, re-probing with FLAG or HA antibodies, and IP with control IgG (middle and lower panels, respectively) confirmed the specificity of these assays. Input protein is shown on the far right panel. **b)**. The D2HGDH self-association was further confirmed by analysing the proteins in denaturing vs. non-denaturing conditions. Again, FLAG- or HA-tagged D2HGDH WT and mutant constructs were co-transfected in HEK-293 cells (the different combinations of WT and mutant proteins are listed on the top). On the left, using three different antibodies and denaturing conditions D2HGDH expression is readily identified at its predicted size. On the right panels, non-denaturing gels revealed a larger product (indicated by brackets) detected every time D2HGDH is ectopically expressed (note its absence in the HA-G131X expressing cells, when IB with HA, and in all instances of empty HA and FLAG vectors co-expression). The D2HGDH self-interacting complex was detected between two D2HGDH-WT molecules (2nd lane from the left), as well as between D2HGDH-WT and any of the four missense D2HGDH mutants (A208T, R212W, R421H, A426T). At the moment we can not exclude the possibility that the self-assembly of D2HGDH includes more than two molecules. (i.e., homotrimer, homotetramer, etc.) or that other proteins are part of this complex (heterotrimer, etc.). *,non-specific band. Red arrow – D2HGDH at its predicted size.

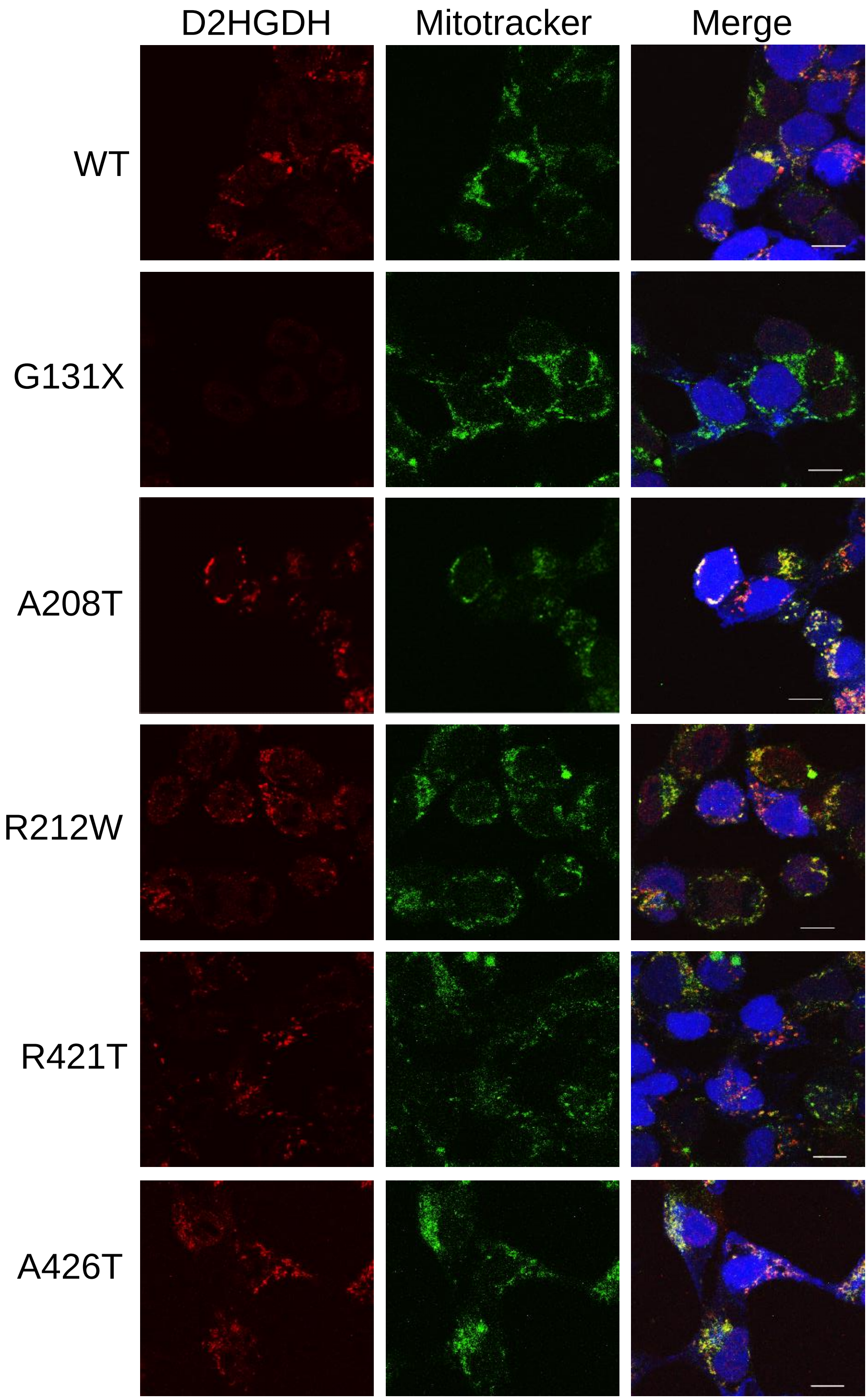


**Supplementary Figure 3. D2HGDH mitochondrial localization by immunofluorescence**. HEK-293 cells stably expressing WT or each D2HGDH mutant were co-stained with the mitochondrial probe MitoTracker (green), and with an anti-D2HGDH antibody (Cy3, red). D2HGDH-expressing cells are also GFP positive (pseudocolored in blue). Merged images indicate that, as expected, WT D2HGDH localizes to the mitochondria, and that the mutations analysed do not change this subcellular localization. As the D2HGDH antibody targets a C-terminus epitope, the truncated G131X mutant is not recognized and thus its subcellular distribution cannot be determined. Cells were imaged on an Olympus FV1000 confocal microscope, at 60x magnification (scale bar is 10μm).

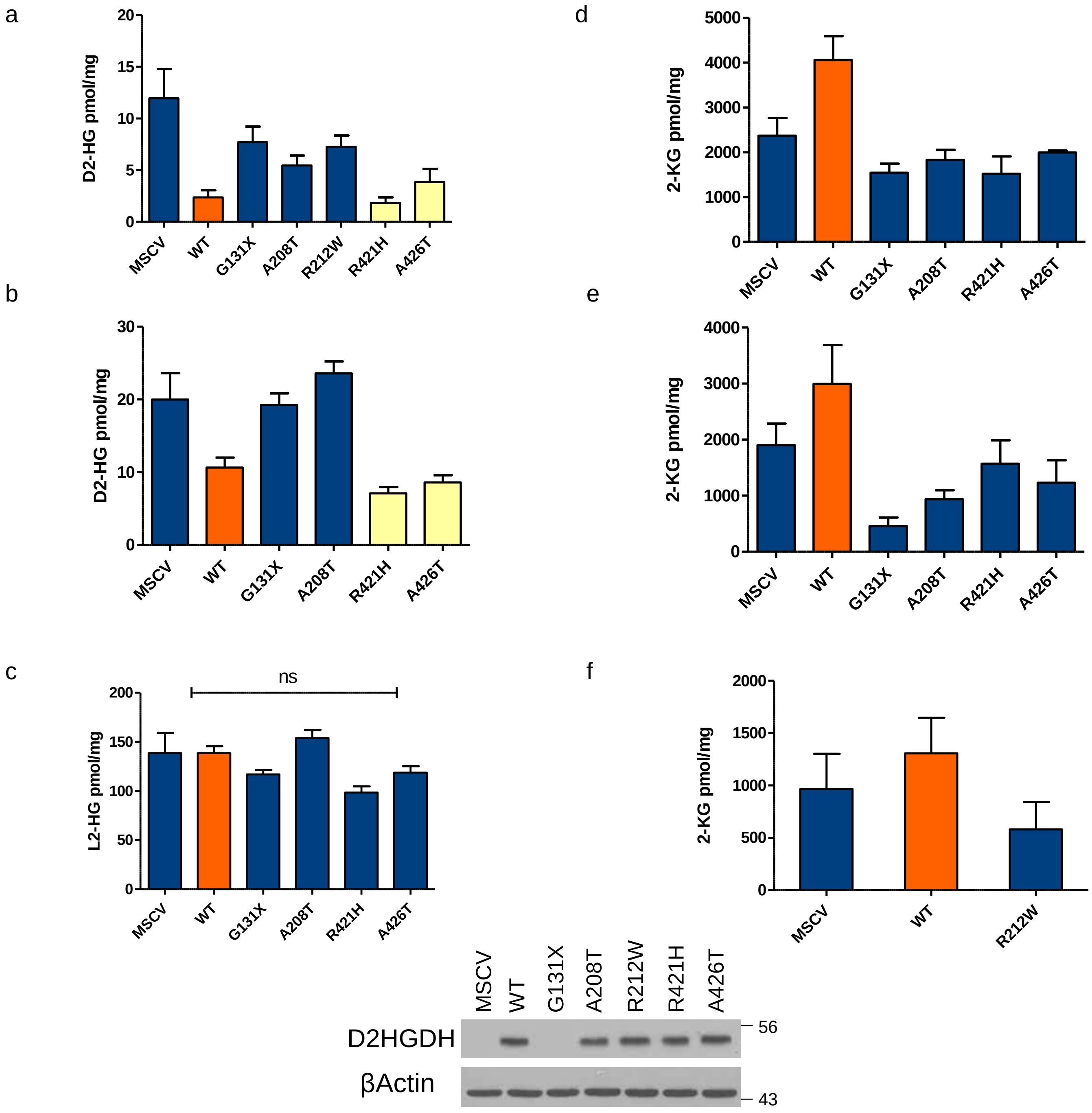


**Supplementary Figure 4. HPLC-ESI-MS-based quantification of D2-HG and α-KG.** Metabolites were extracted from HEK-293 cells stably expressing WT or mutant D2HGDH constructs. In **a)** and **b)** we show that expression of WT D2HGDH led to a decrease in D2-HG when compared to cells expressing the G131X, R208T, and R212W mutants (but not R421H or A426T) (in a, $p<0.0001$, in b, $p=0.0002$, ANOVA; in both $p<0.05$ Bonferroni's Multiple Comparison post-test). Data in a and b represent two biologically independent assays, performed with five and three replicates/sample type, respectively. In **c)**, we show that expression of D2HGDH (WT or mutant) does not alter the levels of L2-HG - assay performed with three replicates/sample type. In **d)**, **e)** and **f)**, we show that expression of D2HGDH-WT result in a significant accumulation of α-KG when compared to cells expressing an empty or any of the DLBCL-associated mutants (in c and d, $p< 0.0001$, in e $p=0.0112$, ANOVA; in all three assays $p<0.05$ Bonferroni's Multiple Comparison post-test). Data in d, e and f, represent three biologically independent assays, performed with four or five replicates/sample type. The data from panel a) and d) are shown in Figure 1a, depicting the relative abundance of D2-HG and α-KG in models of stable D2HGDH expression. Western blot (bottom) confirms the similar expression of WT and mutant D2HGDH – the antibody used is directed to the C-terminus region and would not detect the G131X truncated protein, which is likely to be unstable (see supplementary Figure 3). Metabolites quantifications were normalized by protein amount (mg).

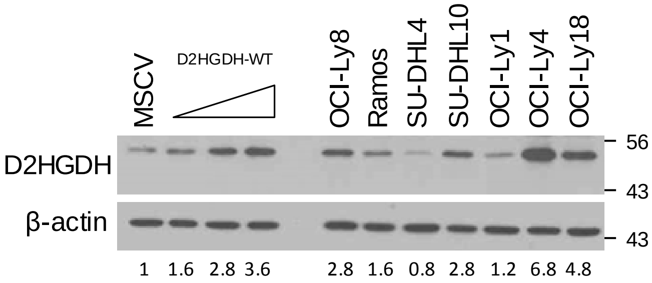


**Supplementary Figure 5. D2HGDH expression levels in the transient transfection model are within endogenous expression range**. Western blot analysis of D2HGDH in HEK-293 cells transiently transfected with an empty vector (MSCV, 1µg), or increasing amounts of a MSCV-D2HGDH construct (0.5µg, 0.75 µg and 1.0 µg) alongside a subset of the B cell lymphoma cell lines used in this study. This assay demonstrated that the expression level achieved in the transient transfection assay is within the range of endogenous D2HGDH expression found in B lymphoma cell lines. Densitometric quantification, normalized by β-actin, is show at the bottom.

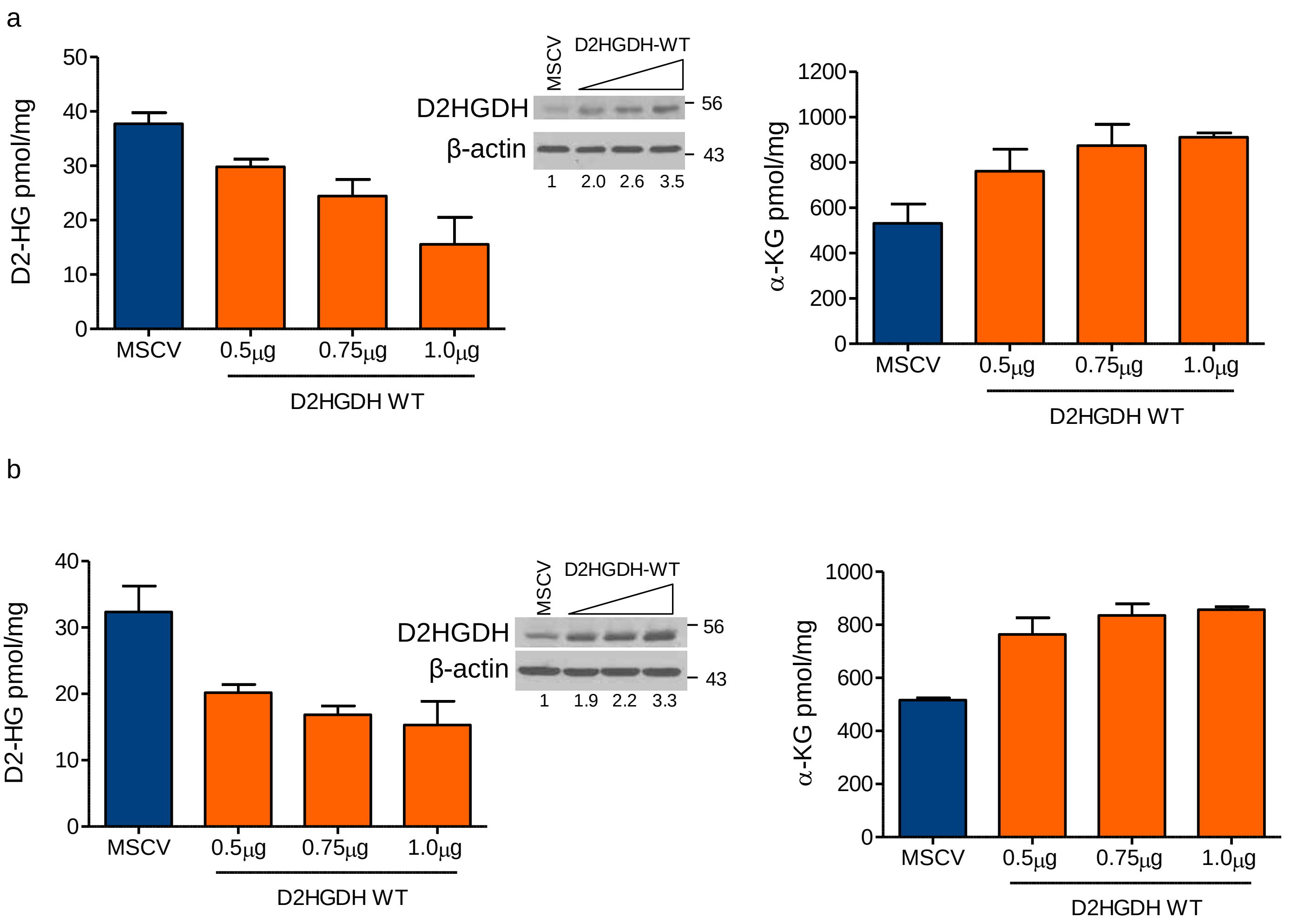


**Supplementary Figure 6. HPLC-ESI-MS-based quantification of D2-HG and α-KG in cells transiently expressing wild-type D2HGDH.** Metabolites were extracted from HEK-293 transiently expressing an empty vector or a WT D2HGDH construct. **a)** and **b)** represent two independent transient transfection assays, and in each instance the left panel depicts D2-HG level and the right panel, α-KG. Western blot examination confirmed the subtle increase in D2HGDH expression in this model, which resulted in a progressive, significant, decrease in D2-HG levels (left panels in **a)** and **b)**, $p=0.0002$ and $p=0.0003$, respectively – ANOVA). Concordantly, increasing D2HGDH expression significantly elevated the levels of α-KG in these cells (right panels, in **a)** and **b)**, $p=0.0068$ and $p<0.0001$, respectively – ANOVA). All data shown represent mean and SD of three replicates/sample type. The data from panel a) are shown in Figure 1d, which depicts the relative abundance of D2-HG and α-KG. Densitometric quantification, normalized by β-actin, is show at the bottom of the western blots.

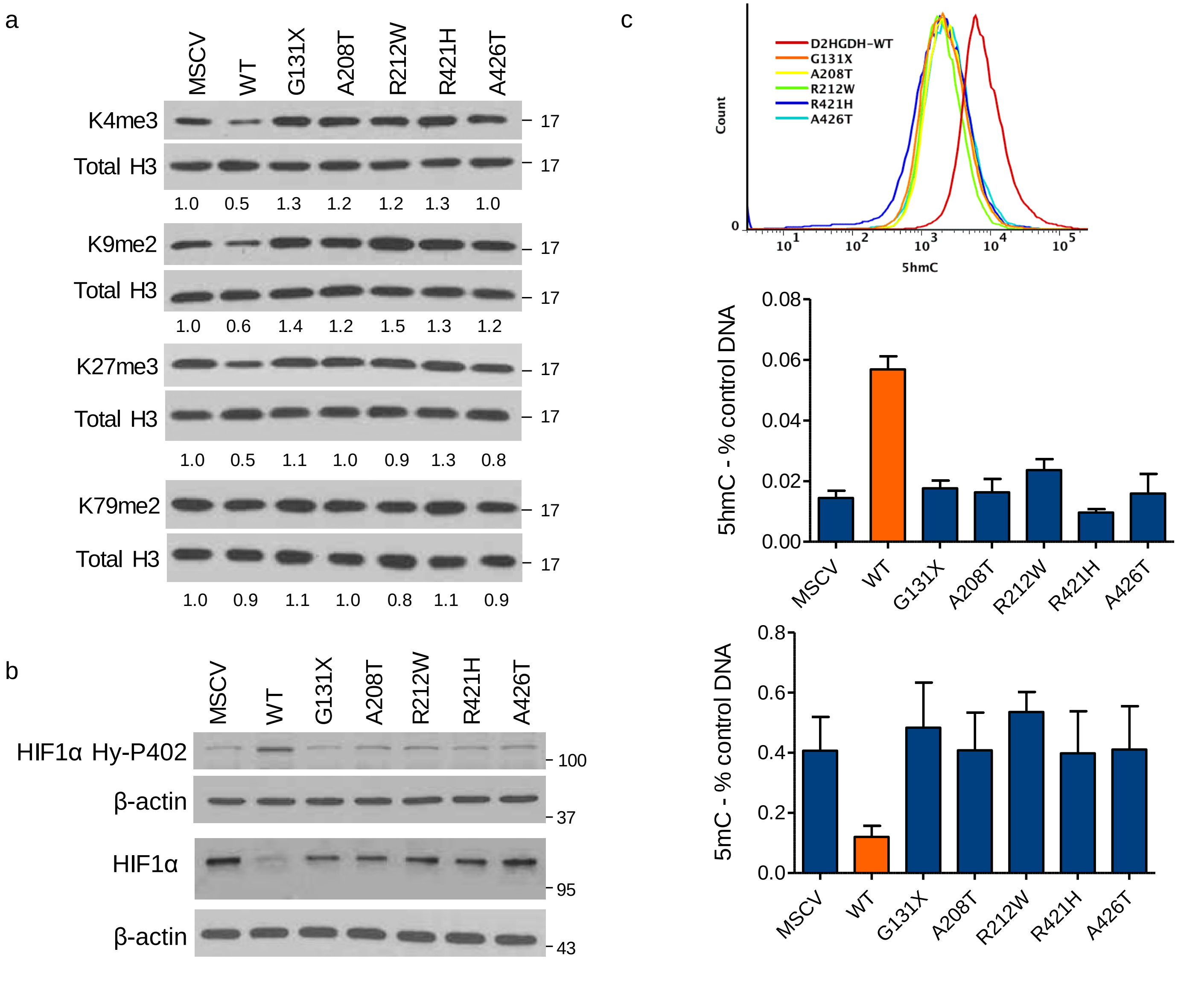


**Supplementary Figure 7. Differential effects of WT and mutant D2HGDH towards α-KG-dependent dioxygenases a) Histone demethylases**: the methylation of H3 lysine residues was determined by western blot in HEK-293 stably expressing WT or mutant D2HGDH; whereas the WT enzyme suppressed K4, K9, K27 methylation, expression of the mutant proteins had little or no effect, and mimicked the empty MSCV control cells. Note that the H3K79me2 level is not influenced by D2HGDH, as this lysine is not regulated by the Jumonji family of α-KG-dependent demethylases. Densitometric quantification, normalized by β-actin, is show at the bottom of each panel. **b) HIF1α prolyl-hydroxylases**: under hypoxia (1% $O_2$, 16h), the levels of HIF1α hydroxylation (Pro-402) were increased and total HIF1α markedly decreased in cells expressing WT but not any of the mutant D2HGDH enzymes. **c) TET enzymes**: Top panel: The levels of 5hmC were quantified by intracellular FACS and found to be lower in cells expressing mutant D2HGDH than in WT cells, as shown by the left-shift of D2HGDH-mutant expressing cells in the histogram. Mid panel: The quantification of 5hmC marks was also performed using a capture and detection antibody-based approach. Abundance of 5hmC marks was measured by absorbance, and reported as relative values normalized by a positive control, a polynucleotide containing 20% 5hmC. Cells expressing empty vector (MSCV) or any of the D2HGDH mutant enzymes had significantly lower levels of 5hmC- modified DNA than the D2HGDH WT cells ($p<0.001$, one-way ANOVA, $p<0.05$, Bonferroni's multiple comparison post-test). These data represent the mean and SD of an assay performed in triplicate, confirmed in a biological replicate. Bottom panel) The quantification of 5mC was performed as above; DNA methylation was significantly higher in mutant than in WT D2HGDH cells ($p<0.001$, one-way ANOVA, $p<0.05$, Bonferroni's multiple comparison post-test). The data show represent the combined values of three biological replicates, performed in triplicate or duplicate. The western blot data shown in a) and b) were confirmed in biological replicates at least three times, and all results independently validated with a transient transfection model (Figure 1).

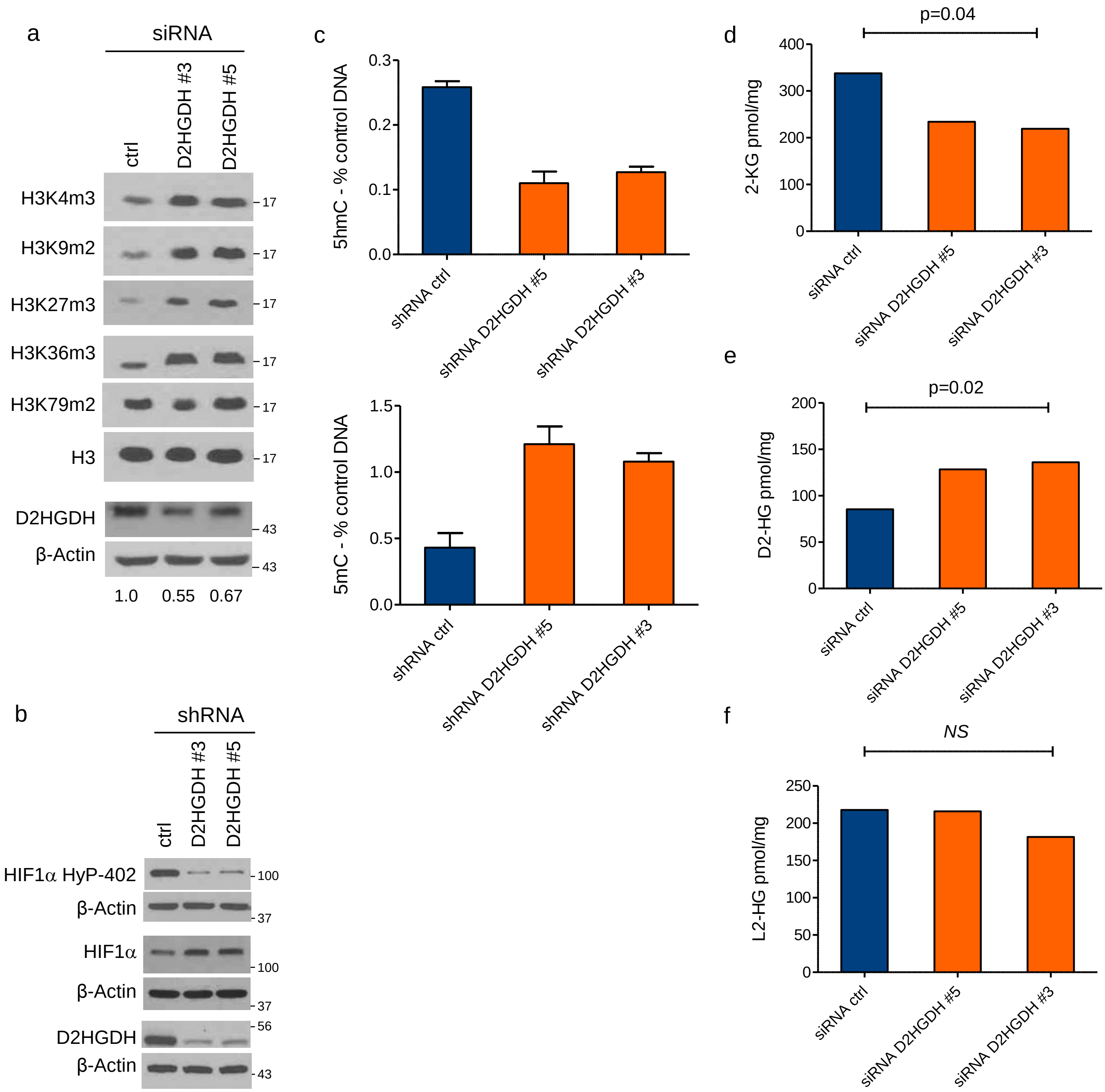


**Supplementary Figure 8. Transient and stable knockdown (KD) of D2HGDH in HEK-293 cells. a)** Transient transfection of two siRNA oligonucleotides directed at D2HGDH or a control siRNA led to increase in the methylation levels of H3K4, H3K9, H3K27 and H3K36; but not in H3K79 which is not regulated by α-KG-dependent HDMs. **b)** Upon exposure to hypoxic conditions for 16h, cells with stable shRNA-based KD of D2HGDH showed marked suppression of hydroxylated HIF1α with consequent accumulation of total HIF1α. Efficacy of the transient (siRNA) or stable (shRNA) KD of D2HGDH is shown by western blot (a and b). Note that the transient KD only partially suppresses D2HGDH expression (~60%). Densitometric quantifications are shown at the bottom. **c)** The levels of the DNA marks 5hmC and 5mC (top and bottom panels) were significantly lower and higher, respectively, in D2HGDH-KD cells than their controls ($p<0.0001$, ANOVA); **d)**, **e)** and **f)** show that the transient, partial, KD of D2HGDH significantly modify D2-HG ($p= 0.04$, ANOVA), α-KG ($p=0.02$, ANOVA), but not L2-HG levels ($p=0.4$, ANOVA), as determined by LC/MS. The 5hmC and 5mC measurements shown represent the mean and SD of five data points derived two independent assays. The LC/MS data shown is the mean of an experiment performed with two replicates; similar results were obtained in an independent assay performed with a single replicate.

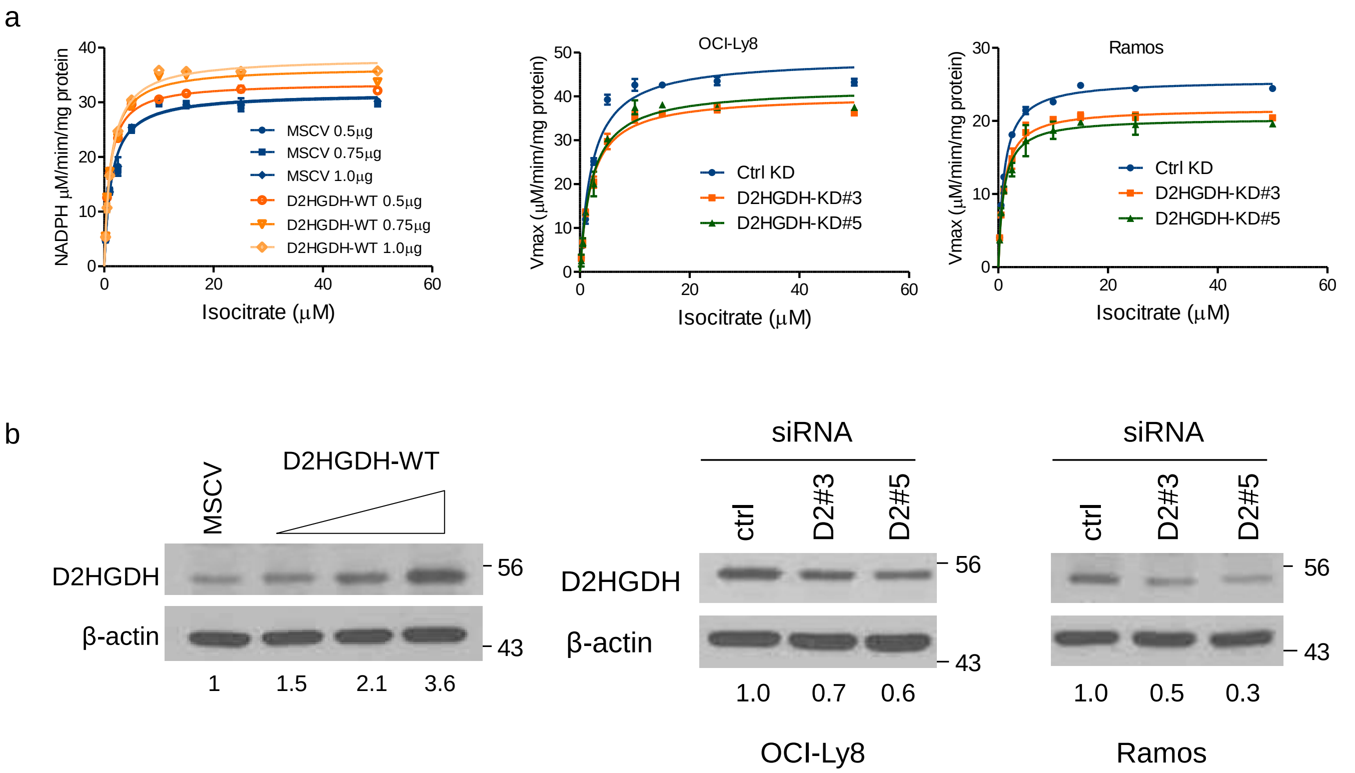


**Supplementary Figure 9. D2HGDH expression and IDH activity a)** Left - Transient expression of increasing amounts of WT D2HGDH significantly and progressively increased IDH activity in comparison to that of MSCV-expressing isogenic cells ($p<0.0001$ ANOVA, $p<0.05$ Bonferroni's Multiple Comparison post-test). Middle and Right panels - Transient knockdown of D2HGDH in the B cell lymphoma cell lines significantly decreased IDH activity in comparison to isogenic controls ($p<0.0001$; $p=0.0003$; ANOVA, for OCI-Ly8 and Ramos, respectively - $p<0.05$ Bonferroni's Multiple Comparison post-test). In **b)** western blot-based examination of D2HGDH expression in the proteins used for the IDH activity assay (shown in a) confirms its progressive elevation in the transient transfection of HEK-293 cells, and partial knockdown in the B cell lymphoma cell lines OCI-Ly8 and Ramos. Densitometric analysis is shown below the western blot.

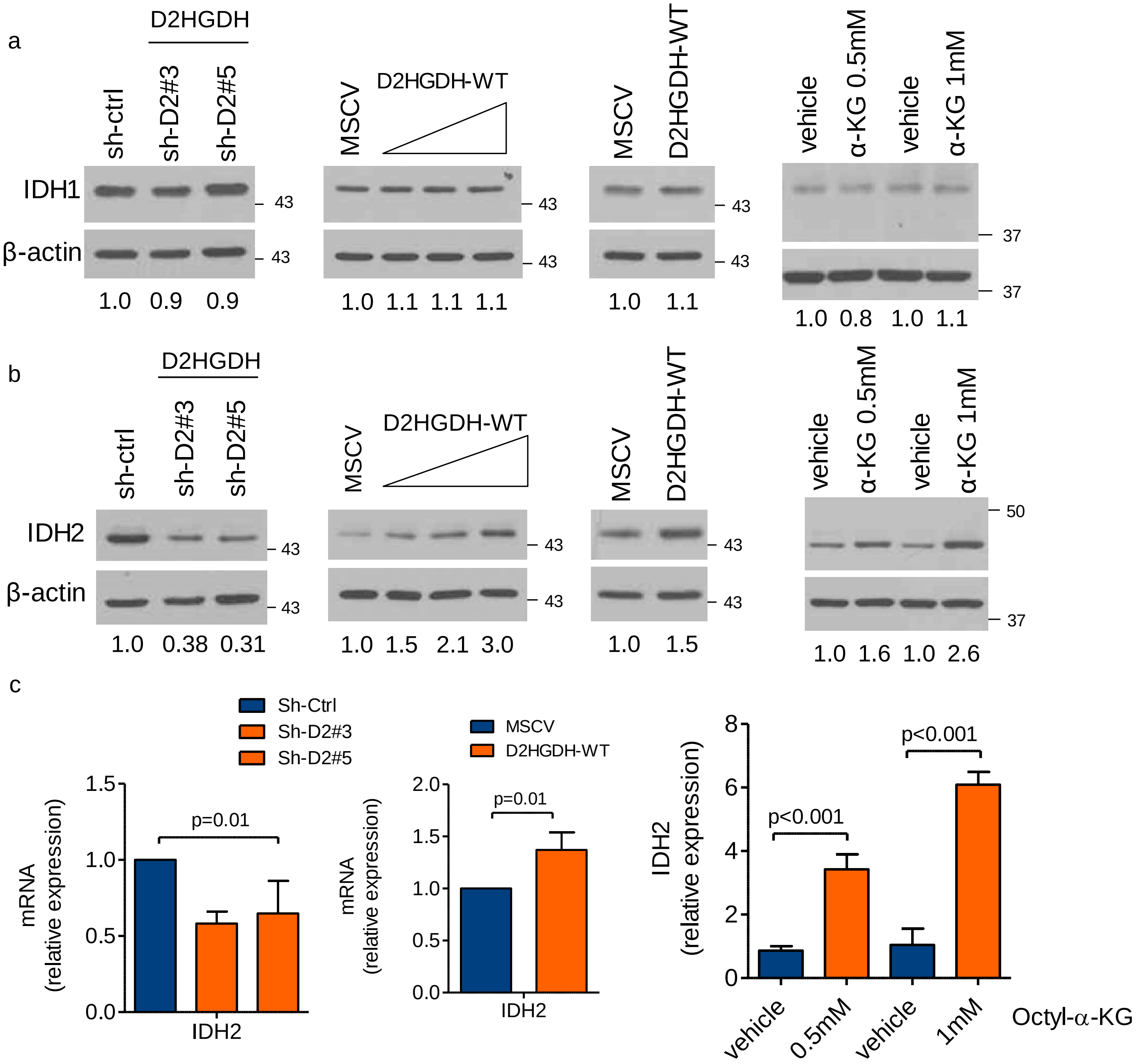


**Supplementary Figure 10. IDH1 and IDH2 expression in models of gain and loss of D2HGDH and following exposure to synthetic cell-permeable α-KG**. **a)** Western blots show that the expression of IDH1 is not modified by D2HGDH knockdown (left panel), its transient or stable ectopic expression (middle panels), or exposure to the cell-permeable octyl-α-KG (right panel). **b)** Western blot analysis show IDH2 downregulation in D2HGDH knockdown HEK-293 cells (left panel), and its upregulation in models of D2HGDH transient or stable expression (middle panels) or upon exposure to the octyl α-KG (right panel). Densitometric quantifications are shown at the bottom of the western blots. These assays were repeated two to four times. **c)** Left panel - real-time RT-PCR of HEK-293 cells with stable D2HGDH knockdown show a significant downregulation of IDH2 (p=0.01, ANOVA). Middle - quantification of IDH2 by real-time RT-PCR demonstrate a significantly higher IDH2 expression in D2HGDH-WT cells than in the empty-vector isogenic controls. (p=0.01, two-tailed Student's t-test). Right - quantification of IDH2 by real-time RT-PCR demonstrate a significantly higher IDH2 expression in HEK-293 cells exposed to synthetic α-KG than vehicle control (p<0.001, two-tailed Student's t-test). The data shown in **c)** (left and middle panels) represent mean and SD of three independent biological replicates (each assay performed in triplicate), displayed as relative expression (sh-ctrl vs. sh-D2HGDH, or MSCV vs. D2HGDH-WT); the data shown in the right panel is the mean -/+ SD of a representative assay performed in triplicate – two independent biological replicates were completed and yielded similar results. In all instances, *IDH2* expression was normalized to that of a housekeeping gene *TBP*, relative quantification achieved by calculating ΔΔCt, and expression defined as $2^{-\Delta\Delta Ct}$, where cells expressing control vector, or vehicle, represent the baseline.

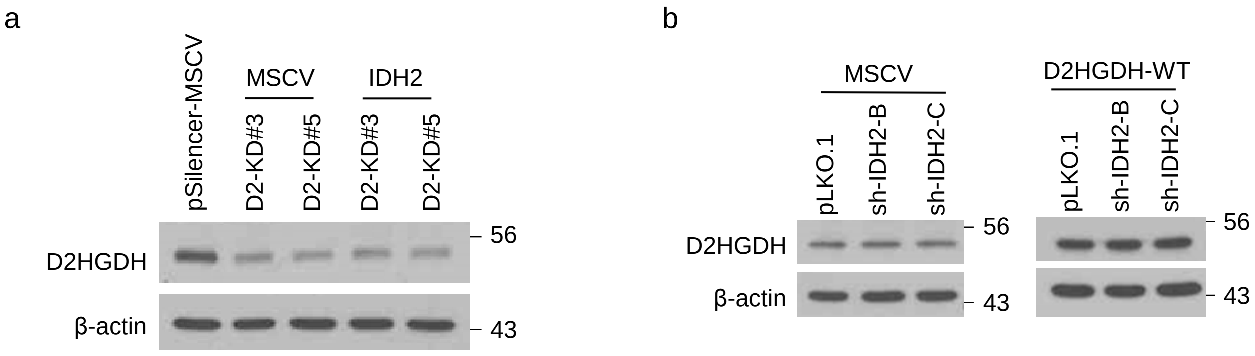


**Supplementary Figure 11. D2HGDH levels in models of IDH2 ectopic expression or knockdown. a)** Western blot analysis shows that ectopic expression of IDH2 in models of D2HGDH knockdown (KD#3 and KD#5) does not change its expression – compare levels of D2HGDH in KD#3 and KD#5 MSCV-only cells vs. KD#3 and KD#5 in IDH2 cells. Expectedly, the D2HGDH levels are lower in KD#3 and KD#5 cells than in control pSilencer/MSCV cells. **b)** shRNA-mediated knockdown of IDH2 did not change D2HGDH expression levels in MSCV control cells (left panel) or in their isogenic counterparts expressing WT D2HGDH (right panel). Expectedly, the levels of D2HGDH are higher in the D2HGDH-WT cells than in MSCV controls.

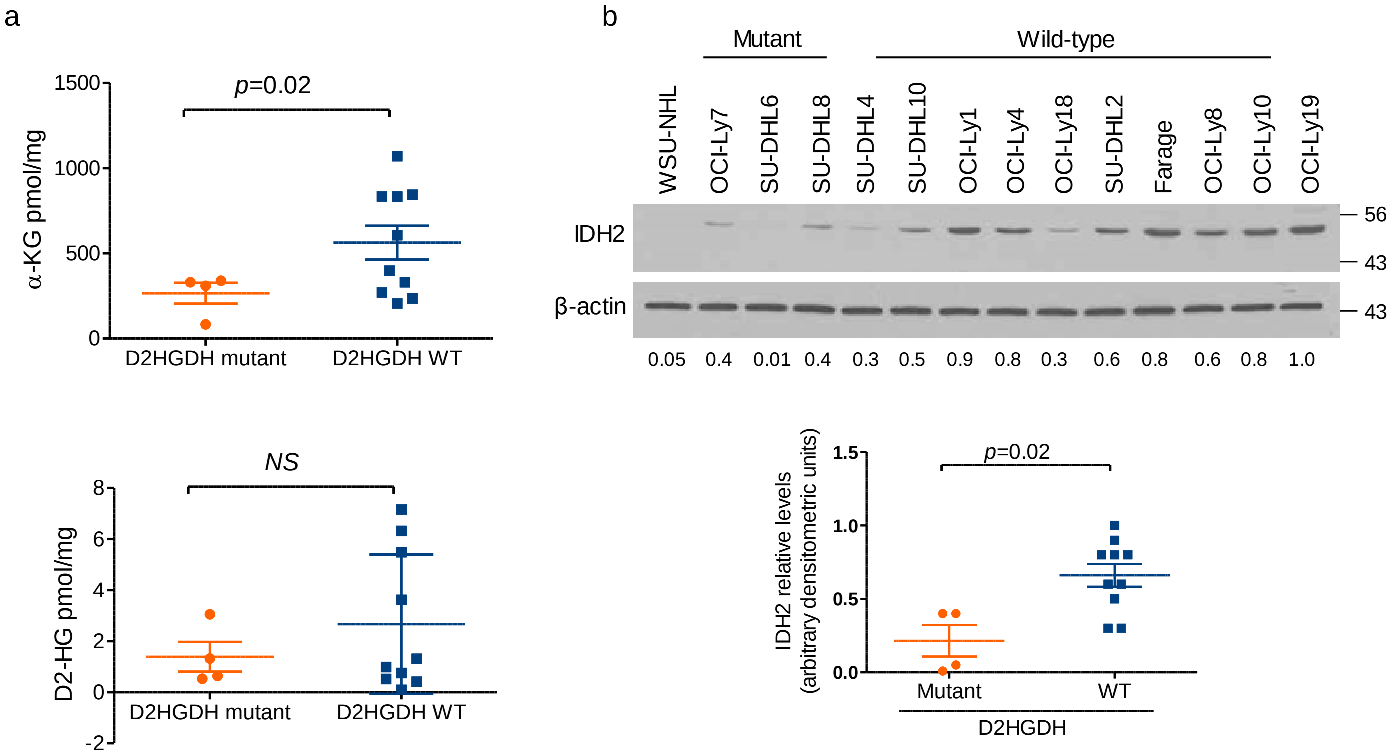


**Supplementary Figure 12**. **Interplay between D2HGDH, α-KG and IDH2 in DLBCL**. **a)** HPLC-ESI-MS-based quantification of α-KG and D2-HG in 14 parental DLBCL cell lines showed significantly lower levels of α-KG in D2HGDH mutant DLBCLs than in WT cells, but no significant difference in the abundance of D2-HG. For each cell line, the data shown represent the median value of three independent assays, each performed in triplicate (p=0.02, two-tailed Student's t-test). **b)** Western blot analysis of IDH2 in a set of 14 DLBCL cell lines, including four D2HGDH mutant and 10 wild-type. Densitometric quantification of IDH2 (normalized by β-actin) is shown below the blots and in graphic display with its statistical significance denoted (Mann-Whitney test). The metabolite quantification in a) was normalized by protein concentration.

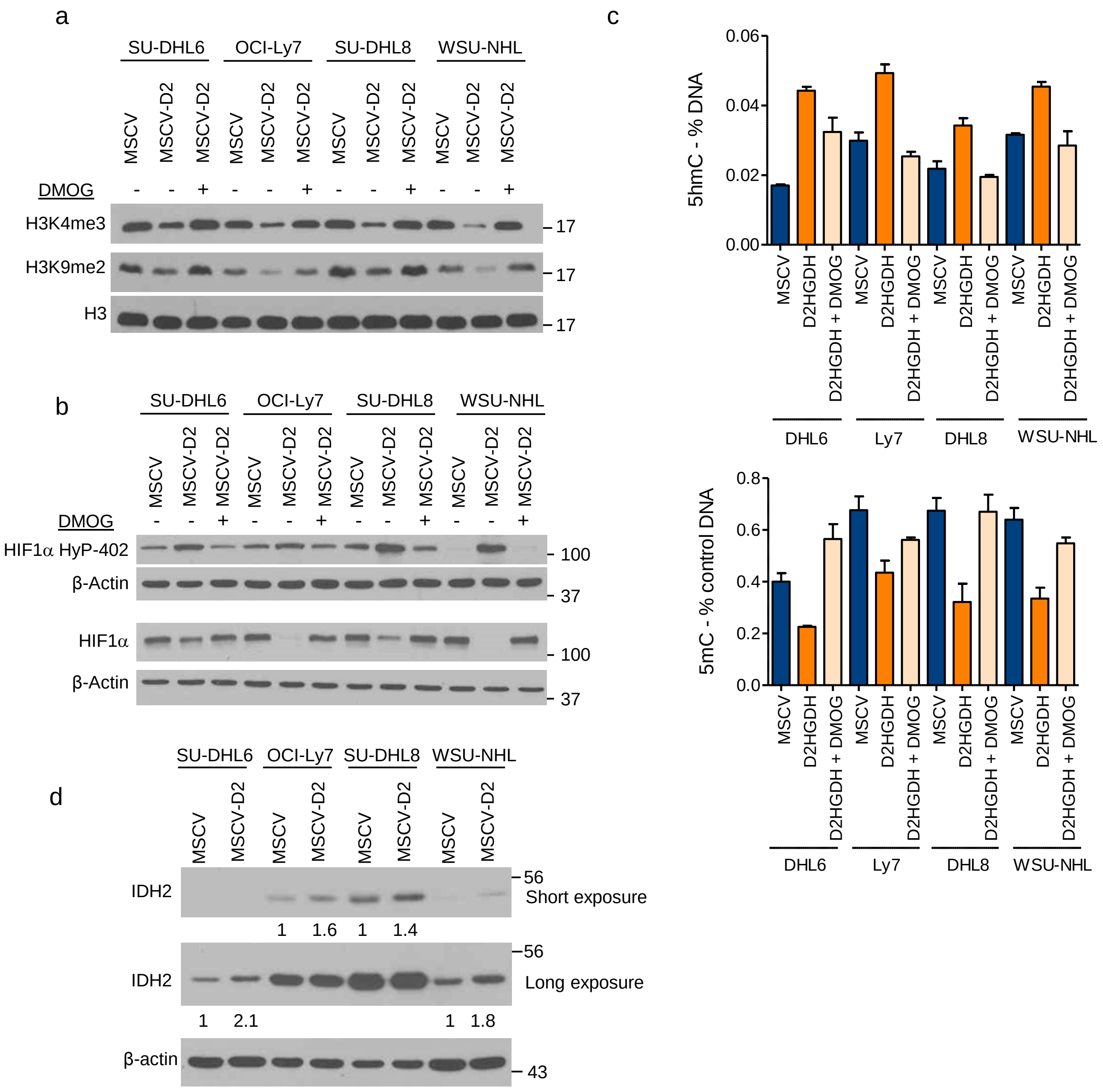


**Supplementary Figure 13. Genetic modulation and functional analysis of D2HGDH-mutant DLBCL cell lines.** Isogenic models of stable expression of an empty vector (MSCV) or WT D2HGDH were generated by retrovirus transduction in four independent D2HGDH-mutant DLBCL cell lines. **a)** Ectopic expression of D2HGDH decreased H3K4 and H3K9 methylation in all four models, an effect that was largely reversed by exposure to 1mM of dimethyloxalylglycine (DMOG), a competitive inhibitor of α-KG. **b)** The Hydroxylation of proline 402 in HIF1α-was elevated (top panel) and total HIF1α expression decreased (bottom panel) by expression of WT D2HGDH – in both instances the values were returned to baseline following exposure to DMOG. **c)** Quantification of 5hmC or 5mC show that ectopic expression of D2HGDH-WT in DLBCL mutant cells significantly elevated the abundance of 5hmC marks (top) ($p<0.0001$, ANOVA) and lowered total DNA methylation (5mC levels) (bottom) ($p<0.0001$, ANOVA). For both measurements, the effects of D2HGDH were largely abrogated by exposure to DMOG. The abundance of these modifications are reported as relative values normalized by a positive control, a polynucleotide containing 20% 5hmC or 50% 5mC. Experiments show in a) and b) were repeated twice; data in c) represent the mean and SD of a representative experiment (out of two independent assays) performed in triplicate. **d)** Stable ectopic expression of D2HGDH in the four D2HGDH-mutant DLBCL cell lines induced IDH2 expression. Densitometric quantification (normalized by β-actin) is shown at the bottom of the western blots

2b

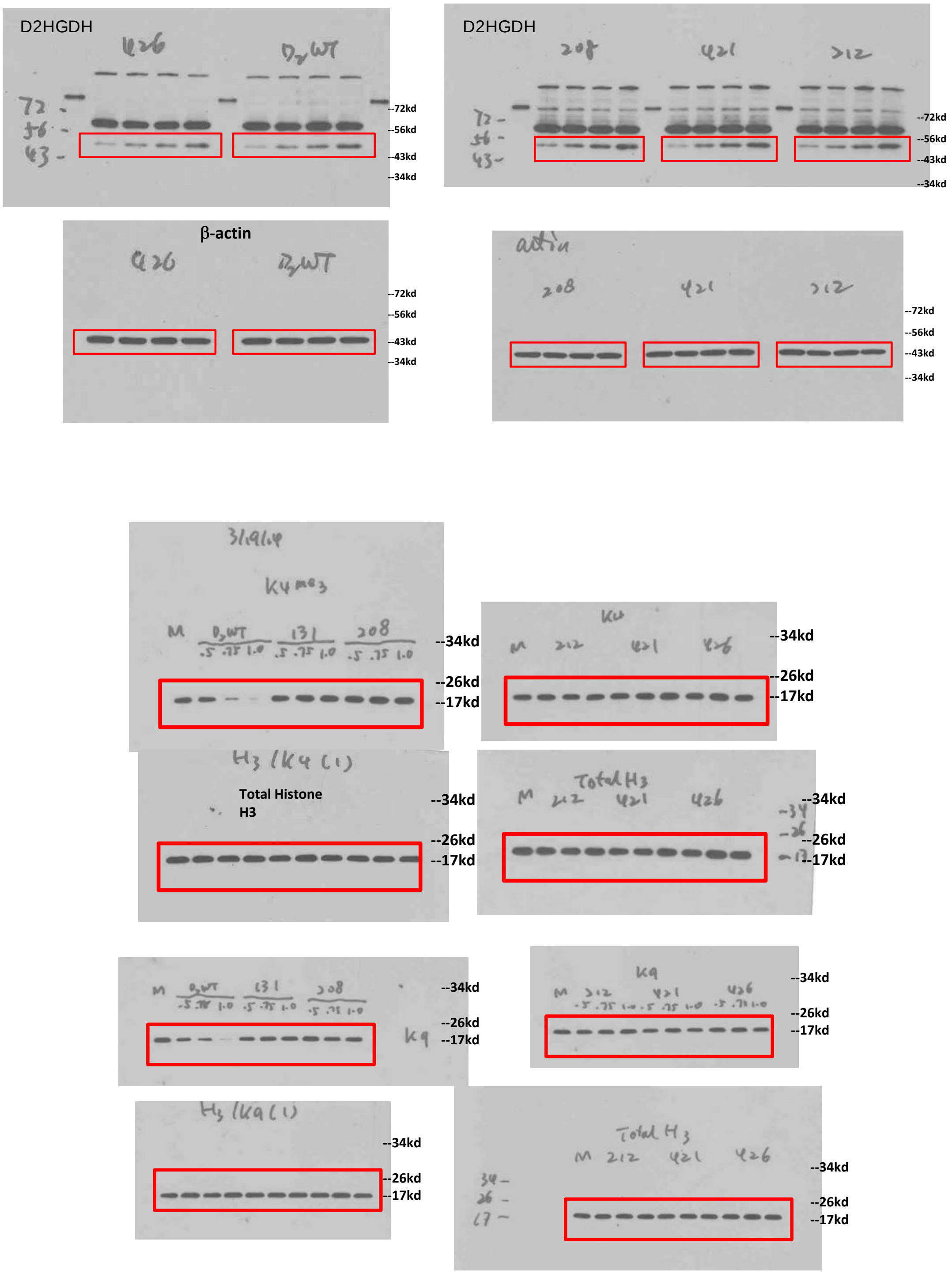


**Supplementary Figure 14. Full scans of uncropped blots presented in the main paper.** Red boxes indicate the cropped regions. Molecular weight markers are indicated in kDa.

2b

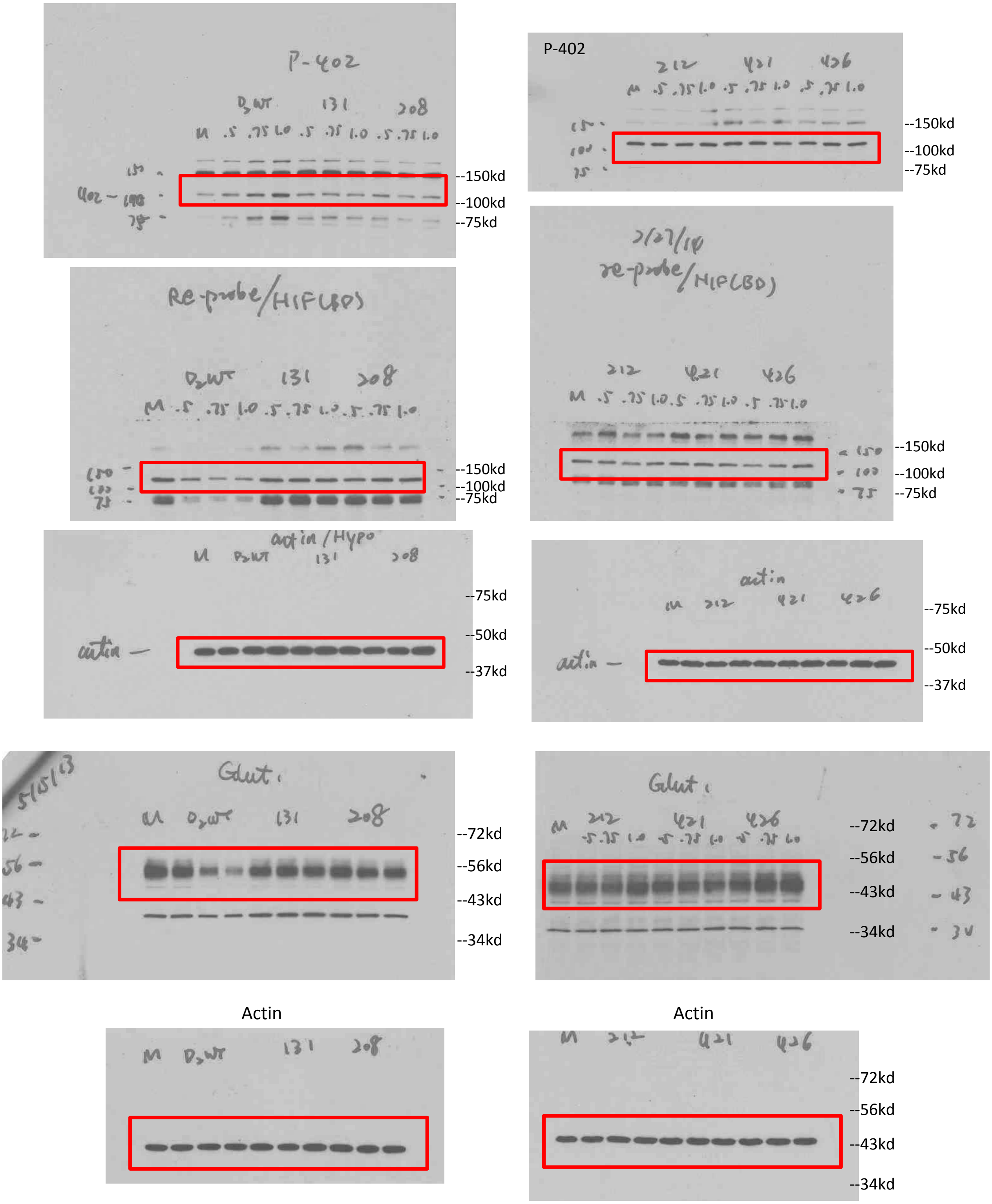


**Supplementary Figure 14 -** continued

3a

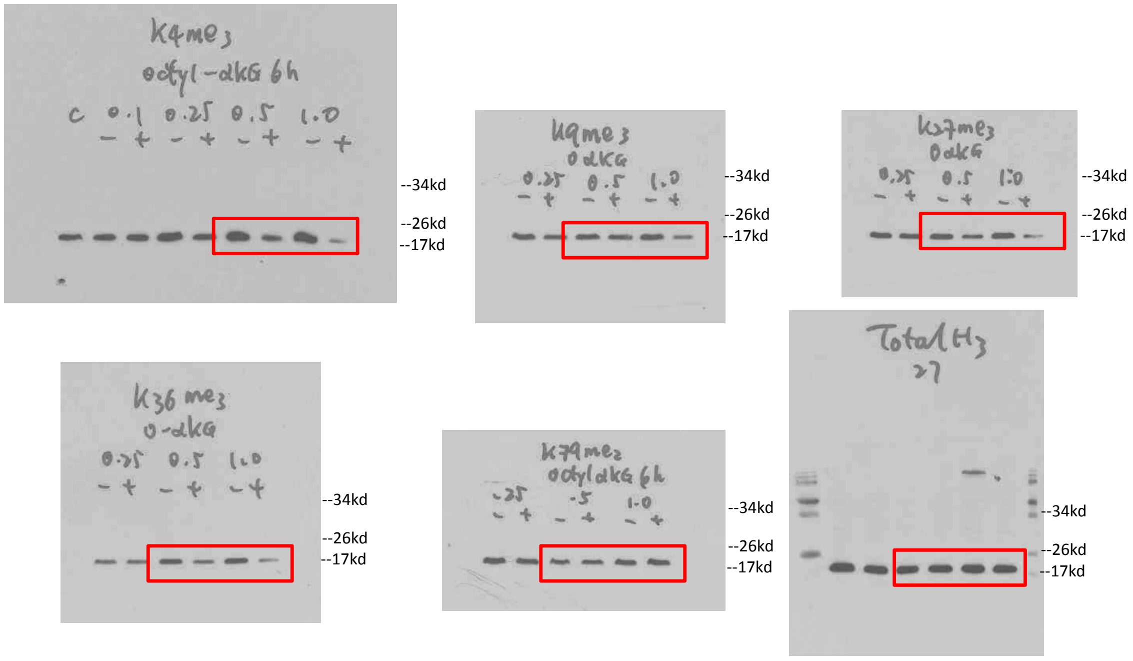


3b

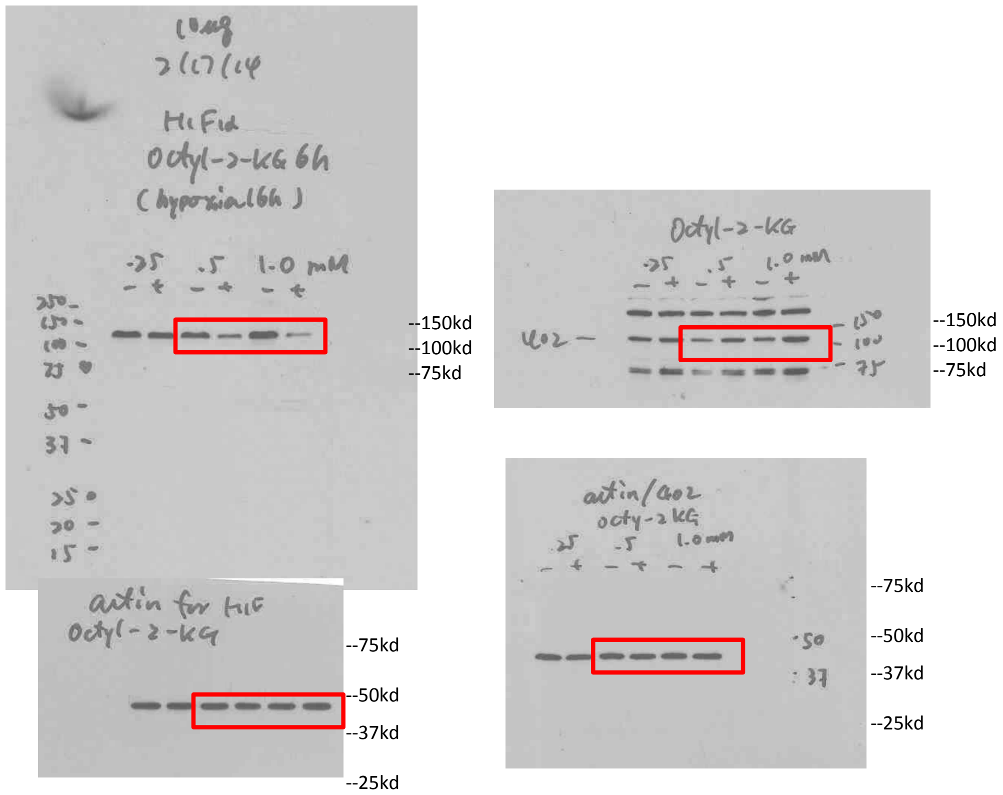


**Supplementary Figure 14** - continued

3d

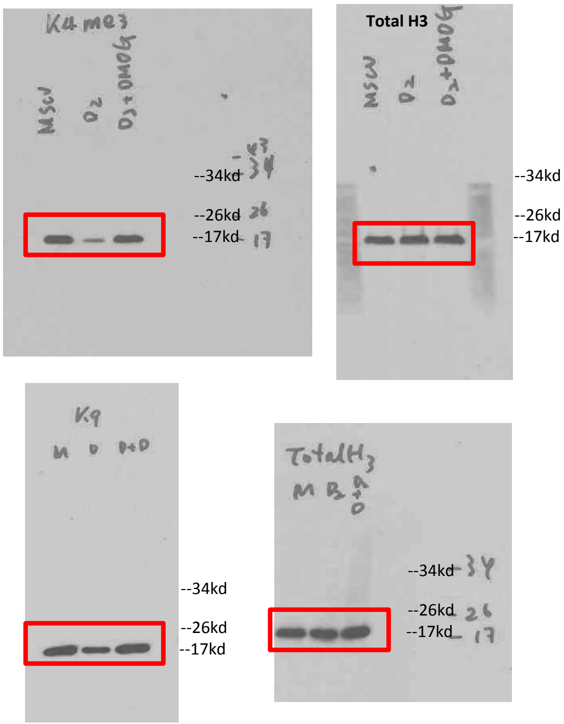


3e

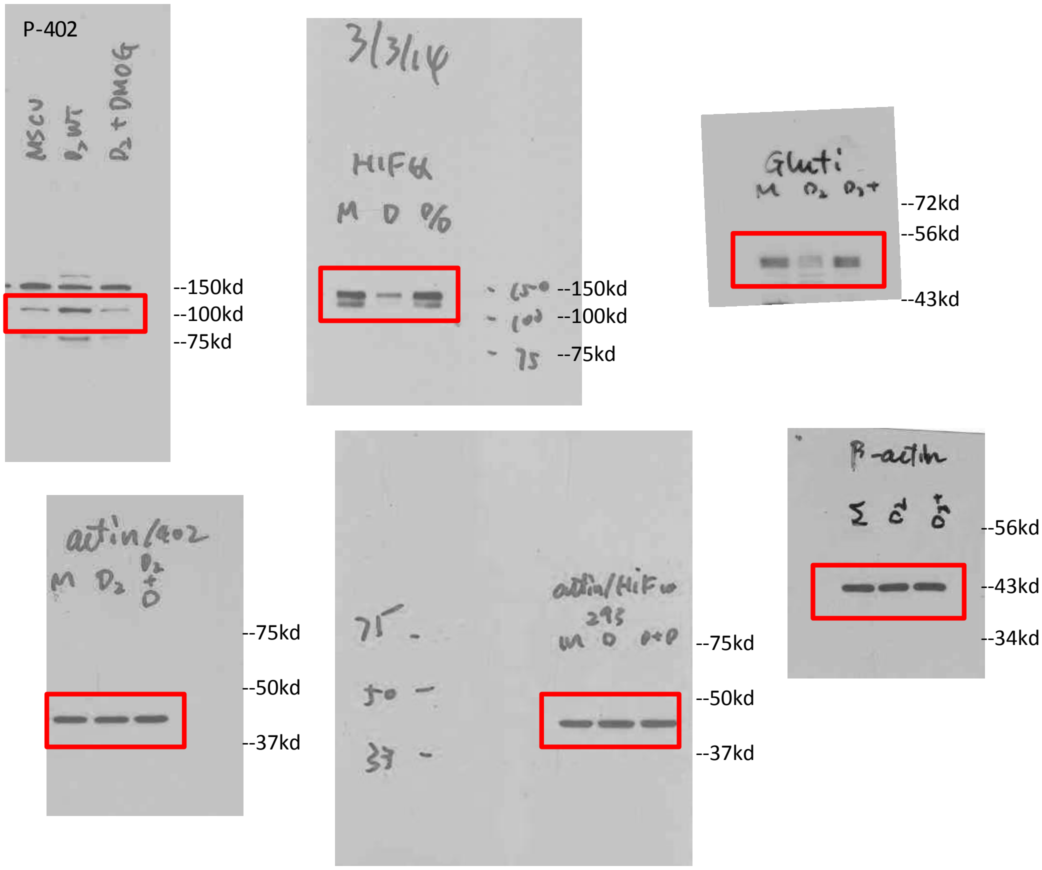


**Supplementary Figure 14 -** continued

4a

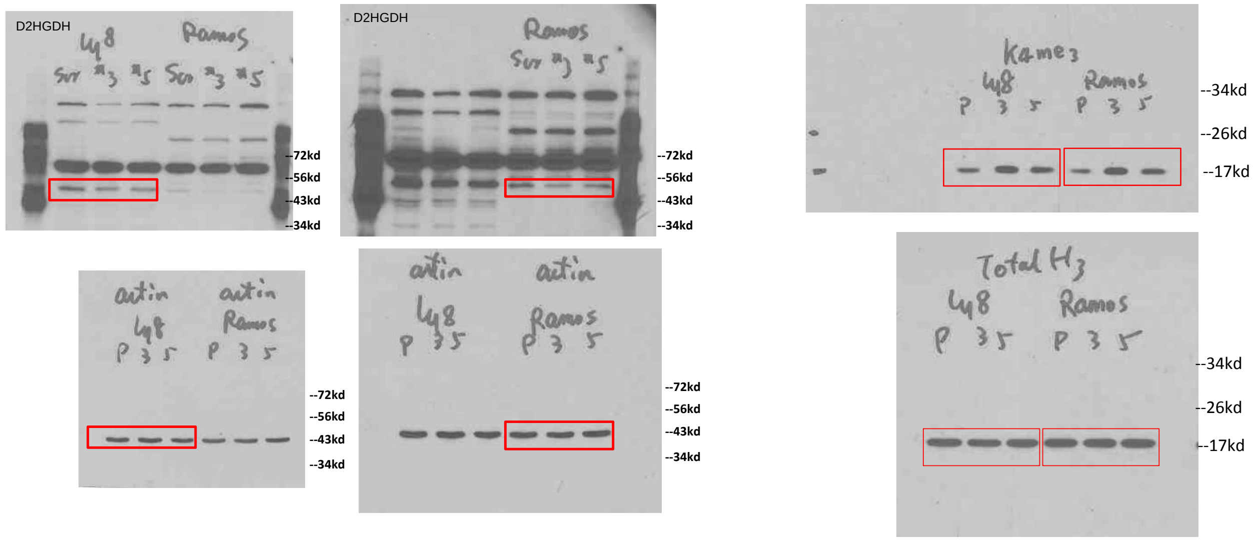


4b

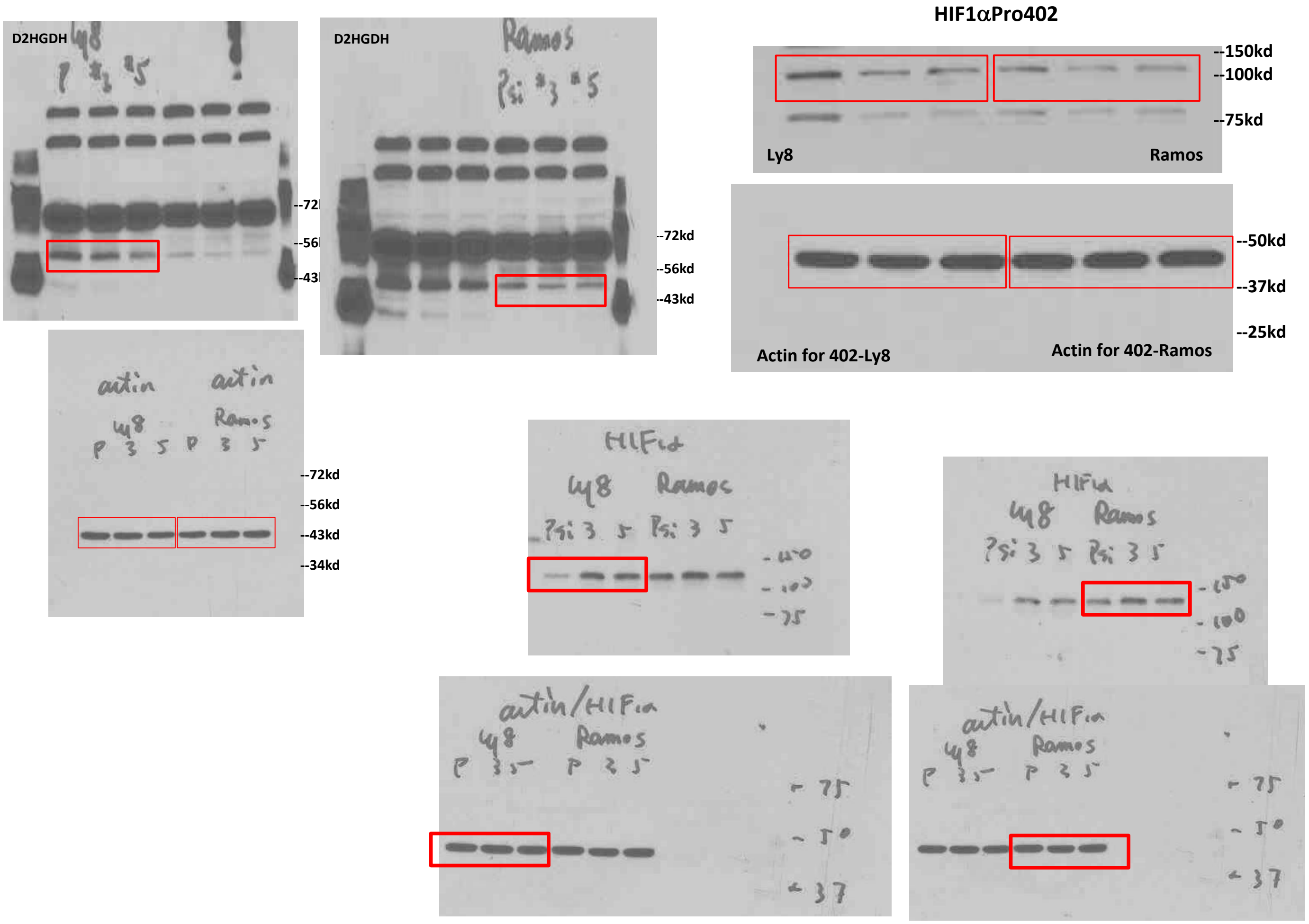


**Supplementary Figure 14** - continued

5a

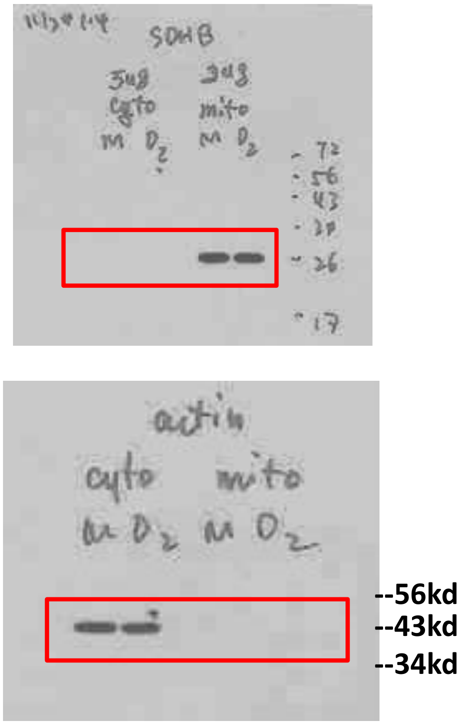


5b

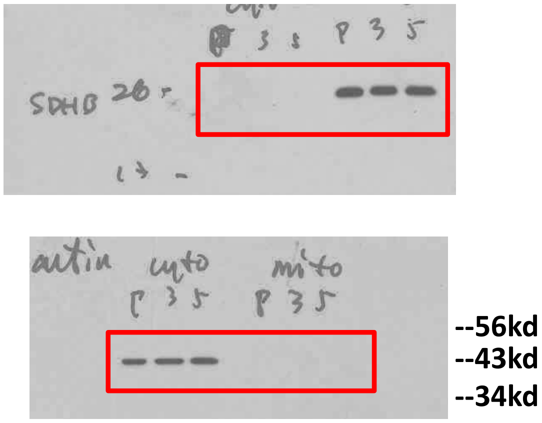


6a

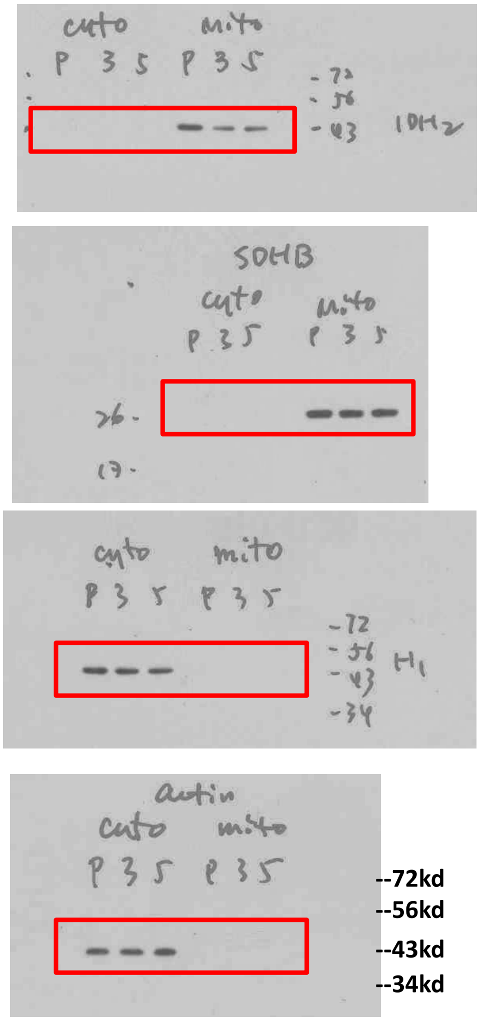


6a

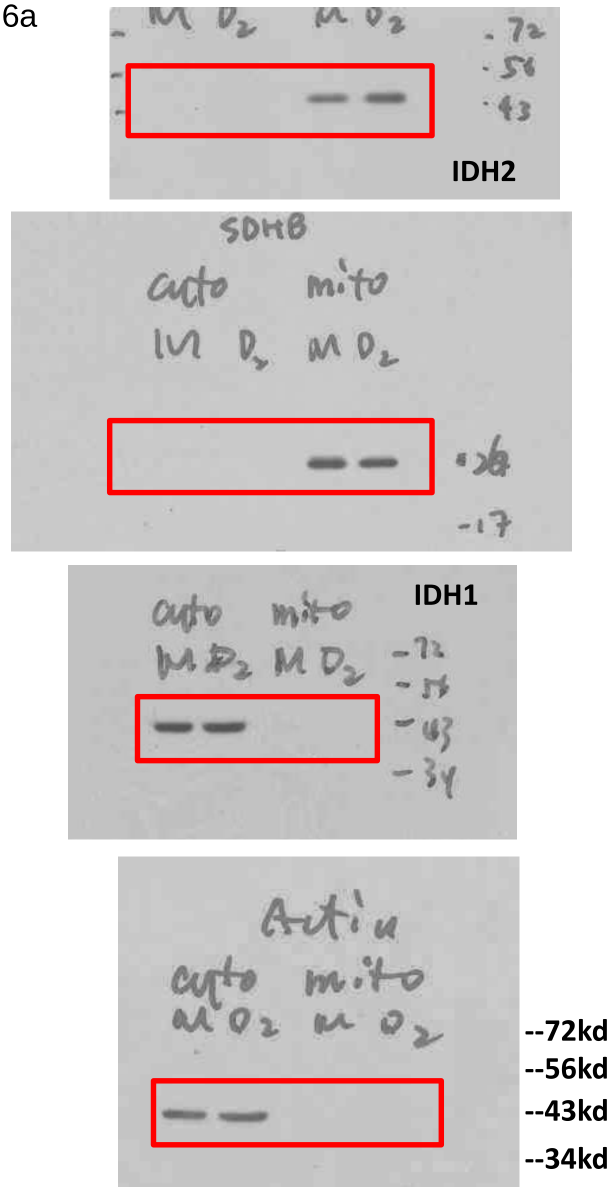


**Supplementary Figure 14** - continued

6b

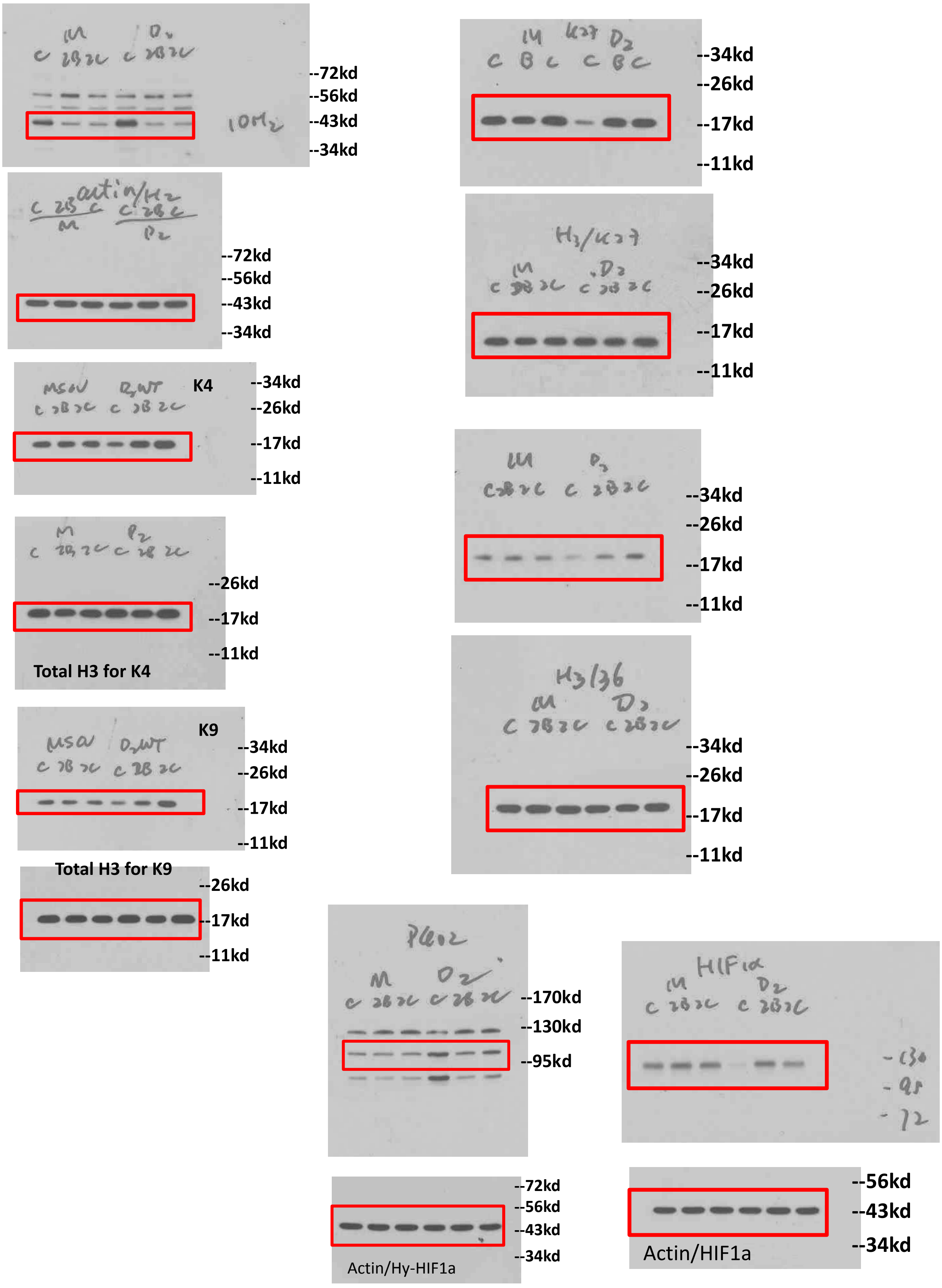


**Supplementary Figure 14** - continued

6d

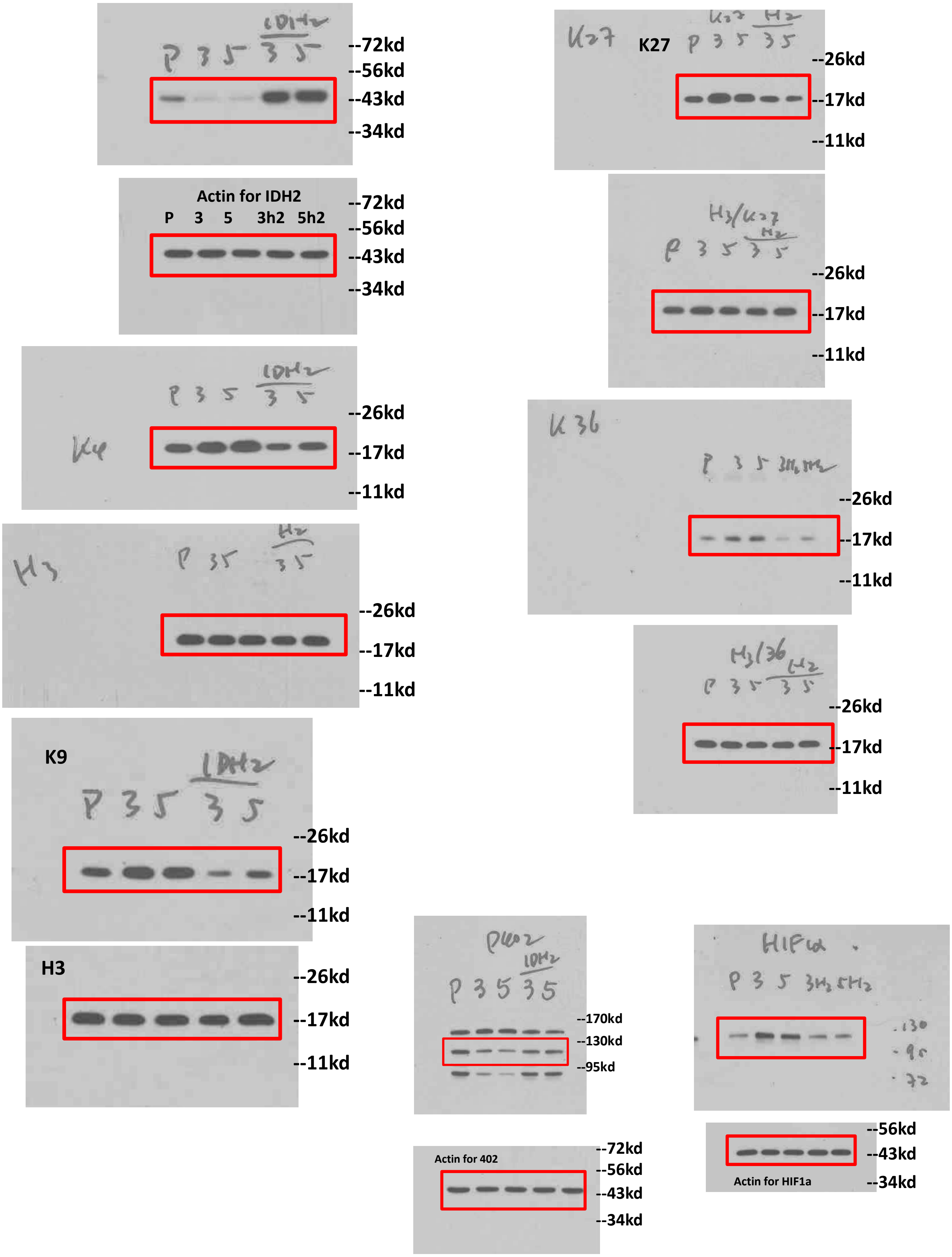


**Supplementary Figure 14** - continued

7a

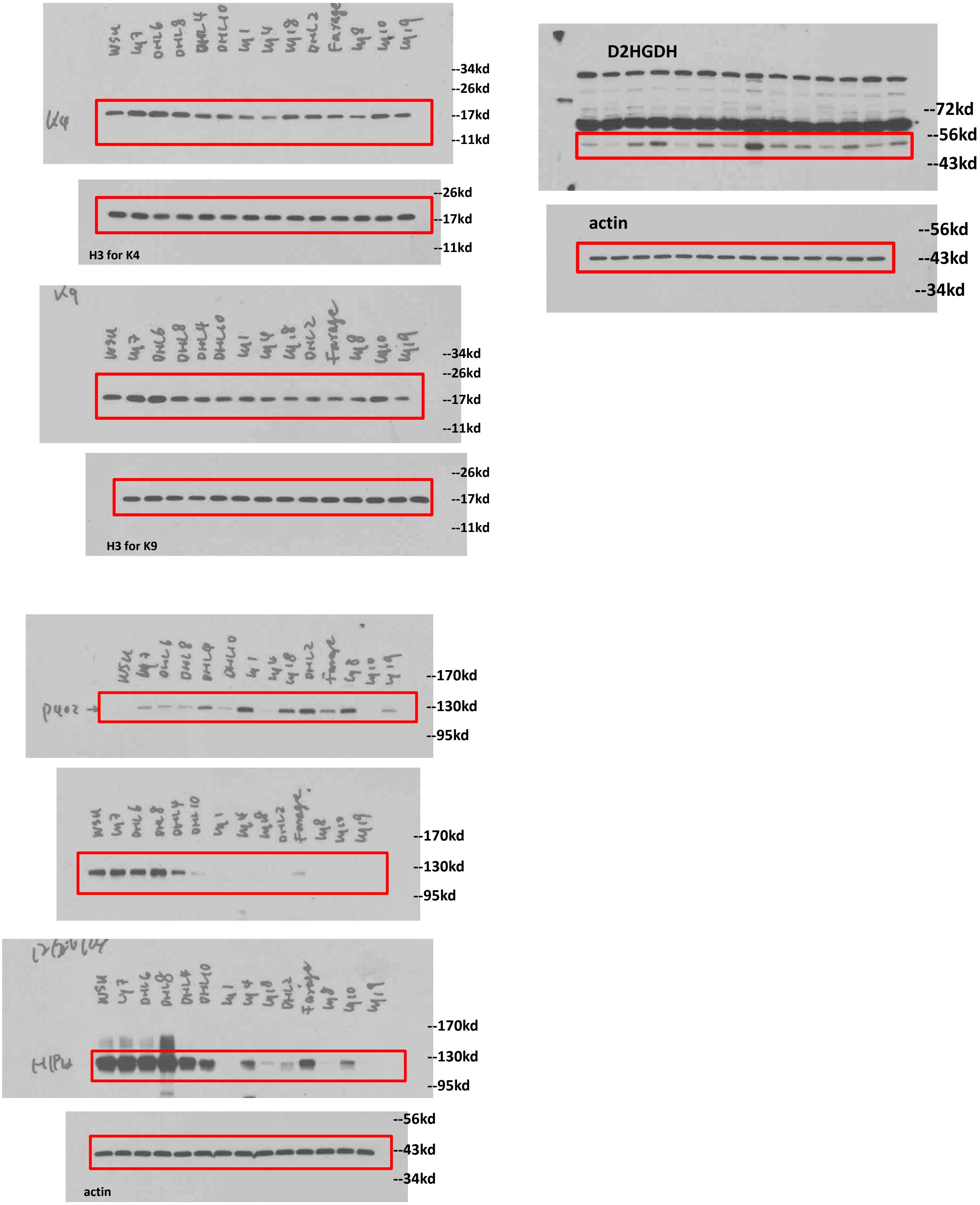


**Supplementary Figure 14** - continued

7c

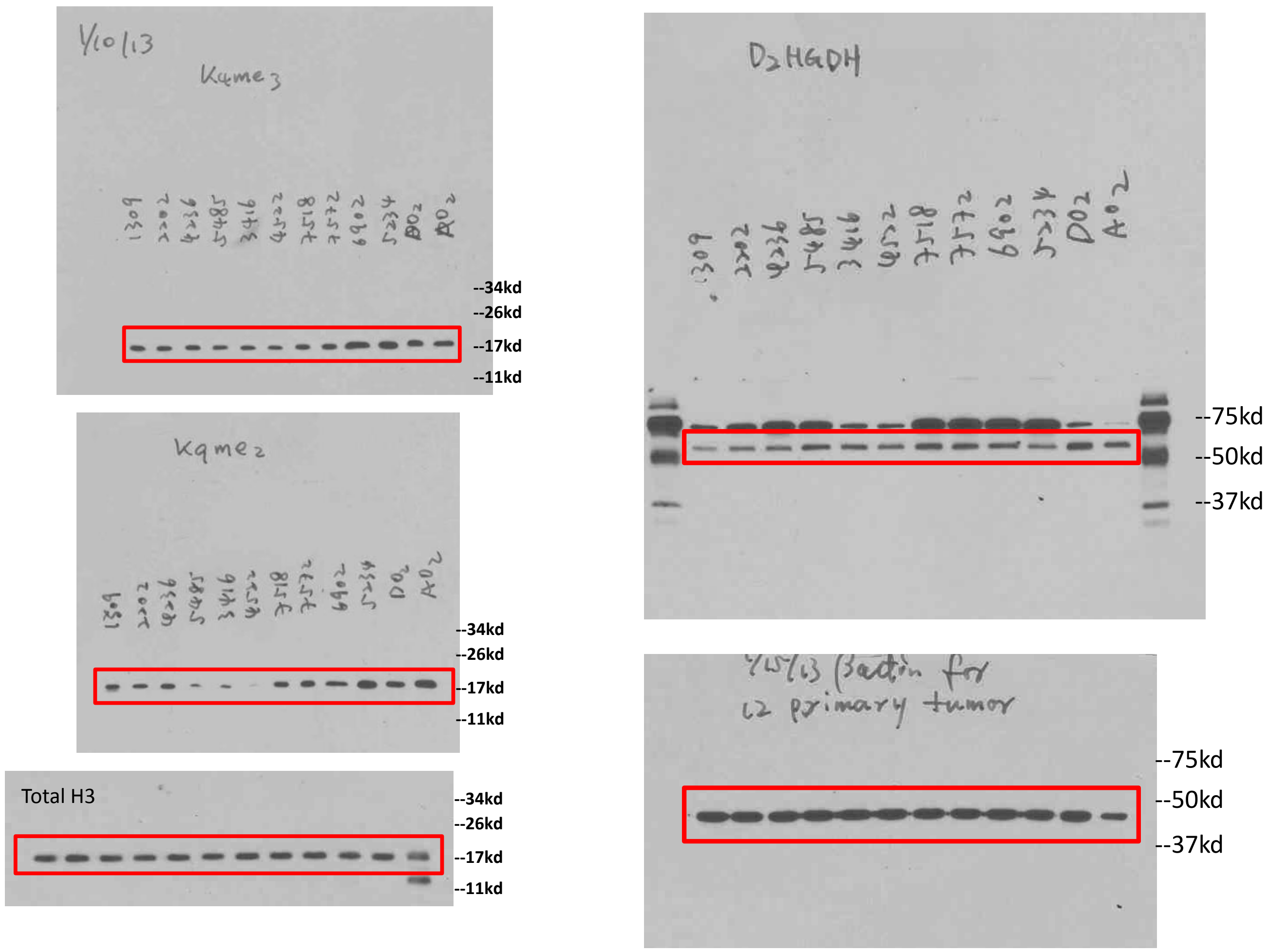


**Supplementary Figure 14** - continued

**Supplementary Table 1 - D2HGDH mutational status in DLBCL**

| Sample ID | Ex2 | Ex3 | Ex4 | Ex5 | Ex6 | Ex7 | Ex8 | Ex9 | Ex10 |
|---|---|---|---|---|---|---|---|---|---|
| 517 | c.292+28T>GT | c.293-18A>G | WT | WT | c.685-9T>CT | WT | c.1012G>AG p.Val338Ile c.1082C>TC p.Ala361Val c.1107T>CT; p.Asp369Asp | WT | c.1307-24A>G |
| 559 | c.292+28T>GT | WT | WT | WT | WT | WT | c.1063G>AG,p.Gly355Ser | WT | c.1307-24A>GA |
| 642 | c.292+28T>GT | c.293-18A>AG | WT | WT | c.685-9T>CT | WT | c.1082C>TC p.Ala361Val | WT | c.1307-24A>GA |
| 865 | c.292+28T>GT | c.293-18A>AG | c.490+40T>CT | WT | c.685-9T>C | WT | c.1012G>AG p.Val338Ile c.1082C>TC p.Ala361Val | WT | c.1307-24A>GA |
| 1057 | c.292+28T>GT | c.293-18A>AG | c.490+40T>CT | WT | c.685-9T>CT | WT | c.1012G>AG p.Val338Ile c.1107T>CT; p.Asp369Asp | WT | c.1307-24A>G |
| 1143 | c.292+28T>GT | WT | WT | WT | WT | WT | WT | WT | WT |
| 1309 | c.292+28T>GT | WT | c.351-46G>AG;c.490+40T>CT | WT | c.685-9T>CT | WT | WT | WT | c.1307-24A>GA |
| 1320 | c.164G>AG,p.Arg55Gln c.292+28T>G | c.293-18A>G | c.490+40T>CT | WT | c.685-9T>C | WT | c.1012G>AG p.Val338Ile c.1082C>TC p.Ala361Val c.1107T>CT; p.Asp369Asp | c.1306+68T>CT | c.1307-24A>GA |
| 1326 | c.43C>CG, p.Arg15Gly c.164G>AG,p.Arg55Gln | WT | c.490+40T>CT | WT | c.685-9T>CT | WT | c.1012G>AG p.Val338Ile | c.1306+68T>CT | WT |
| 1423 | c.43C>CG, p.Arg15Gly c.164G>AG,p.Arg55Gln c.292+28T>GT | WT | WT | WT | WT | WT | WT | WT | WT |
| 1479 | c.164G>AG,p.Arg55Gln c.292+28T>GT | WT | WT | WT | WT | WT | WT | WT | WT |
| 1650 | c.43C>CG, p.Arg15Gly c.292+28T>GT | c.293-18A>AG | WT | WT | c.685-9T>CT | WT | c.1082C>TC p.Ala361Val | WT | WT |
| 1750 | c.292+28T>GT | c.293-18A>G | WT | WT | c.685-9T>C | WT | WT | WT | c.1307-24A>G |
| 2202 | c.292+28T>GT | c.293-18A>AG | WT | WT | c.685-9T>CT | WT | c.1082C>TC p.Ala361Val | c.1306+68T>CT | c.1307-24A>GA c.1377C>AC,p.Pro459Pro |
| 2216 | WT | WT | WT | WT | WT | WT | WT | WT | WT |
| 2323 | c.292+28T>GT | WT | WT | WT | c.685-9T>CT | WT | WT | c.1306+68T>CT | c.1307-24A>GA |
| 2485 | WT | WT | WT | WT | WT | WT | WT | WT | WT |
| 2633 | c.292+28T>GT | c.293-18A>AG | WT | WT | c.685-9T>C | WT | c.1082C>T p.Ala361Val | c.1272G>AG p.Pro424Pro | c.1307-24A>GA |
| 2992 | c.1-91 C>GC c.292+28T>GT | c.293-18A>AG | c.490+40T>CT | WT | c.685-9T>CT | WT | c.1012G>AG p.Val338Ile | WT | WT |
| 3002 | c.292+28T>GT | WT | WT | WT | WT | WT | WT | WT | c.1307-24A>G c.1377C>AC,p.Pro459Pro |
| 3271 | c.164G>AG,p.Arg55Gln c.292+28T>GT | c.293-18A>AG | c.490+40T>CT | WT | c.685-9T>CT | WT | c.1107T>CT; p.Asp369Asp | c.1306+68T>CT | c.1307-24A>G |
| 3279 | c.292+28T>GT | WT | WT | WT | WT | WT | WT | WT | c.1307-24A>GA c.1377C>AC,p.Pro459Pro |
| 3410 | c.164G>AG,p.Arg55Gln | c.293-18A>G | c.490+40T>CT | WT | c.685-9T>C | WT | c.1012G>AG p.Val338Ile c.1082C>TC p.Ala361Va | WT | WT |
| 3416 | c.164G>AG,p.Arg55Gln c.292+28T>GT | WT | c.490+40T>CT | WT | WT | WT | WT | WT | WT |
| 3466 | c.164G>AG,p.Arg55Gln c.292+28T>GT | WT | WT | WT | WT | WT | WT | WT | WT |
| 3522 | WT | c.293-18A>AG | c.490+40T>CT | WT | c.685-9T>CT | WT | c.1012G>AG p.Val338Ile | c.1306+68T>CT | c.1307-24A>G |
| 3533 | c.164G>AG,p.Arg55Gln c.292+28T>GT | WT | c.490+40T>CT | WT | c.685-9T>CT | WT | WT | c.1306+68T>CT | c.1307-24A>GA c.1388A>TA,p.Glu463Va |
| 3717 | c.292+28T>GT | WT | WT | WT | c.685-9T>CT | WT | c.1012G>AG p.Val338Ile c.1082C>TC p.Ala361Va | WT | c.1307-24A>GA |
| 3832 | c.164G>AG,p.Arg55Gln c.292+28T>GT | WT | WT | WT | c.685-9T>CT | WT | c.1082C>T p.Ala361Val | WT | c.1307-24A>GA |
| 4018 | c.164G>AG,p.Arg55Gln | c.293-18A>GA | WT | WT | c.685-9T>CT | WT | c.1082C>T p.Ala361Val | WT | WT |
| 4021 | c.164G>AG,p.Arg55Gln c.292+28T>GT | WT | c.490+40T>CT | WT | c.685-9T>CT | WT | c.1012G>AG p.Val338Ile | WT | c.1307-24A>G |
| 4236 | c.164G>AG,p.Arg55Gln c.292+28T>GT | WT | c.490+40T>CT | WT | c.685-9T>CT | WT | c.1107T>CT; p.Asp369Asp | WT | c.1307-24A>GA |
| 4350 | c.292+28T>GT | c.293-18A>GA | WT | WT | c.685-9T>CT | WT | c.1082C>T p.Ala361Val | WT | WT |
| 4375 | c.164G>AG,p.Arg55Gln c.292+28T>GT | WT | WT | WT | WT | WT | WT | WT | WT |
| 4450 | c.292+28T>GT | WT | WT | WT | WT | WT | WT | WT | c.1307-24A>G |
| 4470 | c.164G>AG,p.Arg55Gln c.292+28T>GT | c.293-18A>GA | c.490+40T>CT | WT | c.685-9T>CT | WT | WT | WT | WT |
| 4494 | c.292+28T>GT | c.293-18A>GA | WT | WT | c.685-9T>CT | WT | c.1082C>T p.Ala361Val | WT | c.1307-24A>G |
| 4522 | c.164G>AG,p.Arg55Gln c.292+28T>GT | c.293-18A>GA | c.490+40T>CT | WT | c.685-9T>CT | WT | WT | WT | c.1307-24A>GA |
| 4554 | c.292+28T>GT | c.293-18A>GA | WT | WT | c.685-9T>CT | WT | WT | WT | c.1307-24A>GA |
| 4756 | c.1-91 C>GC c.292+28T>GT | WT | WT | WT | WT | WT | WT | WT | c.1307-24A>GA c.1377C>AC,p.Pro459Pro |
| 5028 | c.1-91 C>GC c.292+28T>GT | WT | WT | WT | WT | WT | WT | WT | WT |
| 5058 | c.292+28T>GT | c.293-18A>AG | WT | WT | c.685-9T>CT | WT | c.1082C>CT,p.Ala361Val | WT | c.1307-24A>GA |
| 5081 | c.164G>AG,p.Arg55Gln c.292+28T>GT | c.293-18A>G | c.490+40T>CT | WT | c.685-9T>C | WT | c.1082C>TC p.Ala361Val | c.1306+68T>CT | c.1307-24A>GA |
| 5159 | WT | WT | WT | WT | WT | WT | WT | c.1306+68T>CT | c.1307-24A>GA |
| 5160 | c.292+28T>GT | c.293-18A>GA | c.490+40T>CT | WT | c.685-9T>CT | WT | c.1012G>AG p.Val338Ile c.1082C>TC p.Ala361Va | c.1306+68T>CT | c.1307-24A>G |
| 5186 | WT | c.293-18A>G | c.490+40T>CT | WT | c.685-9T>C | WT | c.1082C>TC p.Ala361Val | c.1306+68T>CT | c.1307-24A>GA |
| 5190 | c.164G>AG,p.Arg55Gln | c.293-18A>GA | c.490+40T>CT | WT | c.685-9T>CT c.774G>AG, p.Ser258Ser | WT | c.1012G>AG p.Val338Ile | c.1306+68T>CT | c.1307-24A>GA |
| 5204 | WT | c.293-18A>GA | WT | WT | c.685-9T>CT | WT | c.1082C>TC p.Ala361Val | WT | c.1307-24A>GA |
| 5234 | c.292+28T>GT | c.293-18A>GA | c.490+40T>CT | WT | c.685-9T>CT | WT | c.1012G>AG p.Val338Ile c.1107T>CT; p.Asp369Asp | c.1276G>AG,p.Ala426Thr c.1306+68T>CT | WT |
| 5278 | WT | c.293-18A>GA | WT | WT | c.685-9T>CT | WT | c.1082C>TC p.Ala361Val | WT | c.1307-24A>GA |
| 5283 | WT | c.293-18A>GA | WT | WT | c.685-9T>CT | WT | WT | WT | c.1307-24A>GA |
| 5364 | c.164G>AG,p.Arg55Gln | WT | WT | WT | WT | WT | c.1064G>AG p.Gly355Ser | WT | c.1307-24A>GA |
| 5370 | c.164G>AG,p.Arg55Gln c.292+28T>GT | c.293-18A>GA | WT | WT | c.685-9T>CT; c.853+48G>AG | WT | c.1012G>AG p.Val338Ile | WT | WT |
| 5485 | c.164G>AG,p.Arg55Gln c.292+28T>GT | WT | WT | WT | WT | WT | WT | WT | c.1307-24A>GA c.1377C>AC,p.Pro459Pro |
| 5536 | c.164G>AG,p.Arg55Gln c.292+28T>GT | c.293-18A>GA | WT | WT | c.685-9T>CT | WT | WT | WT | c.1307-24A>GA |
| 5707 | c.164G>AG,p.Arg55Gln c.292+28T>GT | c.293-18A>GA | c.490+40T>CT | WT | c.685-9T>CT | WT | c.1012G>AG p.Val338Ile | WT | WT |
| 5782 | c.292+28T>GT | WT | WT | WT | WT | WT | WT | c.1306+68T>CT | c.1307-24A>GA |
| 5935 | c.164G>AG,p.Arg55Gln | WT | WT | WT | WT | WT | WT | WT | WT |
| 6290 | c.164G>AG,p.Arg55Gln c.292+28T>GT | c.293-18A>G | c.490+40T>CT | WT | c.685-9T>C | WT | c.1082C>TC p.Ala361Val | c.1306+68T>CT | c.1307-24A>GA |
| 6614 | c.164G>AG,p.Arg55Gln c.292+28T>GT | c.293-18A>G | c.490+40T>CT | WT | c.685-9T>C | WT | c.1082C>TC p.Ala361Val | c.1306+68T>CT | c.1307-24A>G |
| 6697 | c.292+28T>GT | c.293-18A>G | WT | WT | c.685-9T>C | WT | c.1082C>T p.Ala361Val | WT | WT |
| 6902 | c.292+28T>GT | WT | c.490+40T>CT | WT | c.685-9T>CT | WT | WT | c.1276G>AG,p.Ala426Thr c.1306+68T>CT | c.1307-24A>G |
| 6971 | c.164G>AG,p.Arg55Gln c.292+28T>GT | c.293-18A>GA | c.490+40T>CT | WT | c.685-9T>CT | WT | c.1012G>AG p.Val338Ile | WT | WT |
| 7041 | c.292+28T>GT | c.293-18A>GA | WT | WT | c.685-9T>CT | WT | c.1082C>T p.Ala361Val | WT | c.1307-24A>GA |
| 7089 | c.164G>AG,p.Arg55Gln c.292+28T>GT | c.293-18A>G | c.490+40T>C | WT | c.685-9T>CT | WT | c.1012G>AG p.Val338Ile c.1104+39C>TC | c.1306+68T>C | c.1307-24A>G |
| 7119 | c.292+28T>GT | c.293-18A>G | c.490+40T>C | WT | c.685-9T>C | WT | c.1012G>AG p.Val338Ile c.1082C>TC p.Ala361Val c.1107T>CT; p.Asp369Asp | c.1306+68T>C | c.1307-24A>G |
| 7295 | c.164G>AG,p.Arg55Gln c.292+28T>GT | WT | WT | WT | WT | WT | WT | WT | WT |
| 7518 | c.292+28T>GT | c.293-18A>G | WT | WT | c.685-9T>C | WT | c.1082C>TC p.Ala361Val | WT | c.1307-24A>GA |
| 7572 | c.292+28T>GT | c.293-18A>GA | c.490+40T>C | WT | c.685-9T>CT | WT | c.1012G>AG p.Val338Ile | c.1306+68T>CT | c.1307-24A>G c.1377C>AC,p.Pro459Pro |
| 7924 | c.292+28T>GT | c.293-18A>G | c.490+40T>C | WT | c.685-9T>C | WT | c.1012G>AG p.Val338Ile | c.1306+68T>C | c.1307-24A>G |
| 7970 | c.292+28T>GT | c.293-18A>GA | WT | WT | c.685-9T>CT | WT | c.1082C>TC p.Ala361Val | WT | WT |
| 7987 | c.164G>AG,p.Arg55Gln c.292+28T>GT | c.293-18A>GA | WT | WT | c.685-9T>CT | WT | c.1082C>TC p.Ala361Val | WT | WT |
| A1 | c.164G>AG p.Arg55Gln, c.292+28T>GT | WT | WT | WT | WT | WT | WT | WT | WT |
| A2 | c.292+28T>GT | c.293-18A>GA | c.490+40T>CT | c.634C>TC p.Arg212Trp | c.685-9T>CT | WT | c.1012G>AG p.Val338Ile | c.1306+68T>CT | c.1307-24A>GA |
| A3 | c.164G>AG p.Arg55Gln, c.292+28T>GT | c.293-18A>GA | c.490+40T>CT | WT | c.685-9T>CT | WT | c.1012G>AG p.Val338Ile | c.1306+68T>CT | c.1307-24A>GA |
| A4 | c.292+28T>GT | WT | WT | WT | WT | WT | WT | c.1306+68T>CT | c.1307-24A>GA |
| A5 | c.164G>AG p.Arg55Gln, c.292+28T>GT | c.293-18A>GA | c.490+40T>CT | WT | c.685-9T>CT | WT | c.1012G>AG p.Val338Ile | c.1306+68T>CT | c.1307-24A>GA |
| A6 | c.164G>AG p.Arg55Gln, c.292+28T>GT | c.293-18A>G | c.490+40T>C | WT | c.685-9T>C | WT | c.1012G>A p.Val338Ile | WT | c.1307-24A>G |
| B1 | c.292+28T>G | c.293-18A>GA | WT | WT | c.685-9T>CT | WT | c.1082C>TC p.Ala361Val | WT | c.1307-24A>GA, c.1377C>AC p.Pro459Pro |
| B2 | c.292+28T>GT | c.293-18A>GA | c.490+40T>CT | WT | c.685-9T>CT | WT | c.1012G>AG p.Val338Ile | c.1306+68T>CT | c.1307-24A>G, c.1377C>AC p.Pro459Pro |
| B3 | c.292+28T>GT | c.293-18A>GA | WT | WT | c.685-9T>C | WT | WT | WT | c.1307-24A>GA |
| B4 | WT | WT | WT | WT | WT | WT | WT | WT | WT |
| B5 | WT | WT | WT | WT | WT | WT | WT | WT | c.1307-24A>GA |
| B6 | WT | WT | WT | WT | WT | WT | WT | WT | WT |
| C1 | c.164G>AG p.Arg55Gln, c.292+28T>G | c.293-18A>G | c.490+40T>CT | WT | c.685-9T>C | WT | c.1012G>AG p.Val338Ile | c.1306+68T>CT | c.1307-24A>G |
| C2 | c.292+28T>GT | c.293-18A>GA | WT | WT | c.685-9T>CT | WT | WT | WT | c.1307-24A>GA |
| C3 | c.164G>AG p.Arg55Gln, c.292+28T>GT | c.293-18A>G | WT | WT | c.685-9T>CT | WT | c.1082C>TC p.Ala361Val | c.1306+68T>CT | c.1307-24A>GA |
| C4 | WT | c.293-18A>GA | c.490+40T>CT | WT | c.685-9T>CT | WT | c.1012G>AG p.Val338Ile | c.1306+68T>CT | c.1307-24A>GA |
| C5 | c.164G>AG p.Arg55Gln, c.292+28T>GT | c.293-18A>GA | c.490+40T>CT | WT | c.685-9T>CT | WT | c.1012G>AG p.Val338Ile | c.1306+68T>CT | c.1307-24A>GA |
| C6 | c.164G>AG p.Arg55Gln, c.292+28T>GT | WT | WT | WT | WT | WT | WT | WT | c.1307-24A>GA |
| D1 | c.292+28T>GT | WT | WT | WT | WT | WT | c.1012G>AG p.Val338Ile | c.1306+68T>CT | c.1307-24A>G |
| D2 | c.164G>AG p.Arg55Gln, c.292+28T>GT | c.293-18A>GA | c.490+40T>CT | c.634C>TC p.Arg212Trp | c.685-9T>CT | WT | c.1012G>AG p.Val338Ile | c.1306+68T>CT | c.1307-24A>GA |
| D3 | c.164G>AG p.Arg55Gln, c.292+28T>G | c.293-18A>G | WT | WT | c.685-9T>C | WT | c.1012G>AG p.Val338Ile,c.1082C>TC p.Ala361Val | c.1306+68T>CT | c.1307-24A>GA |
| D4 | c.164G>AG p.Arg55Gln, c.292+28T>GT | c.293-18A>GA | WT | WT | c.685-9T>CT | WT | WT | c.1306+68T>CT | c.1307-24A>G |
| D5 | c.164G>AG p.Arg55Gln, c.292+28T>GT | c.293-18A>G | c.490+40T>C | WT | c.685-9T>CT | WT | c.1012G>A p.Val338Ile | c.1306+68T>CT | c.1307-24A>G |
| D6 | c.164G>AG p.Arg55Gln, c.292+28T>GT | c.293-18A>GA | c.490+40T>CT | WT | c.685-9T>CT | WT | c.1012G>AG p.Val338Ile | c.1306+68T>CT | c.1307-24A>GA |
| E1 | c.164G>AG p.Arg55Gln, c.292+28T>G | c.293-18A>GA | WT | WT | c.685-9T>CT | WT | WT | WT | c.1307-24A>GA |
| E2 | c.164G>AG p.Arg55Gln, c.292+28T>GT | c.293-23A>TA, c.293-18A>GA | c.490+40T>CT | WT | c.685-9T>CT | WT | c.1012G>AG p.Val338Ile | c.1306+68T>CT | c.1307-24A>G |
| E3 | c.292+28T>GT | c.293-18A>G | c.490+40T>CT | WT | c.685-9T>C | WT | c.1012G>AG p.Val338Ile | c.1306+68T>CT | c.1307-24A>G |
| E4 | c.292+28T>GT | c.293-18A>G | WT | WT | c.685-9T>C | WT | WT | WT | c.1307-24A>G |
| E5 | WT | WT | WT | WT | WT | WT | WT | WT | WT |
| E6 | c.164G>AG p.Arg55Gln, c.292+28T>GT | c.293-23A>TA, c.293-18A>GA | c.490+40T>CT | WT | c.685-9T>CT | WT | c.1012G>AG p.Val338Ile | c.1306+68T>CT | c.1307-24A>GA |
| F1 | c.164G>AG p.Arg55Gln, c.292+28T>GT | c.293-18A>GA | c.490+40T>CT | WT | c.685-9T>CT | WT | c.1012G>AG p.Val338Ile | WT | c.1307-24A>GA |
| F2 | c.164G>AG p.Arg55Gln, c.292+28T>GT | WT | WT | WT | WT | WT | WT | WT | WT |
| F3 | c.292+28T>G | c.293-18A>GA | WT | WT | c.685-9T>CT | WT | c.1038C>TC p.Asn346Asn, c.1082C>TC p.Ala361Val | c.1306+68T>CT | c.1307-24A>GA |
| F4 | c.292+28T>GT | c.293-18A>G | WT | WT | c.685-9T>C | WT | WT | WT | c.1307-24A>G |
| F5 | c.164G>AG p.Arg55Gln, c.292+28T>GT | c.293-18A>GA | c.490+40T>CT | WT | c.685-9T>CT | WT | c.1012G>AG p.Val338Ile | c.1306+68T>CT | c.1307-24A>GA |
| F6 | Fw not good and there is no reverse | c.293-18A>GA | WT | WT | c.685-9T>CT | WT | c.1082C>TC p.Ala361Val | c.1306+68T>CT | c.1307-24A>G |
| G1 | c.164G>A p.Arg55Gln, c.292+28T>G | c.293-18A>G | c.490+40T>C | WT | c.685-9T>C | WT | c.1012G>AG p.Val338Ile, c.1107T>CT,p.Asp369Asp | c.1306+68T>C | c.1307-24A>G |
| G2 | c.164G>AG p.Arg55Gln, c.292+28T>G | c.293-18A>GA | WT | WT | c.685-9T>CT | WT | c.1082C>TC p.Ala361Val | WT | c.1307-24A>GA |
| G3 | c.164G>AG p.Arg55Gln, c.292+28T>GT | WT | WT | WT | WT | WT | WT | WT | WT |
| G4 | c.164G>AG p.Arg55Gln, c.292+28T>G | c.293-18A>G | WT | WT | c.685-9T>C | WT | c.1012G>AG p.Val338Ile,c.1082C>TC p.Ala361Val | c.1306+68T>C | c.1307-24A>G |
| G5 | c.164G>AG p.Arg55Gln, c.292+28T>G | WT | WT | WT | WT | WT | WT | WT | c.1307-24A>GA |
| G6 | c.292+28T>G | WT | WT | WT | WT | WT | WT | WT | c.1307-24A>G |
| H1 | c.164G>AG p.Arg55Gln, c.292+28T>G | WT | WT | WT | WT | WT | WT | WT | WT |
| H2 | c.164G>AG p.Arg55Gln, c.292+28T>GT | WT | WT | WT | WT | WT | WT | WT | c.1307-24A>GA, c.1377C>AC p.Pro459Pro |
| H3 | c.164G>AG p.Arg55Gln, c.292+28T>G | c.293-18A>GA | c.490+40T>CT | WT | c.685-9T>CT | WT | c.1012G>AG p.Val338Ile | WT | c.1307-24A>GA |
| H4 | c.292+28T>GT | c.293-18A>GA | c.490+40T>CT | WT | c.685-9T>CT | WT | c.1012G>AG p.Val338Ile | c.1306+68T>CT | c.1307-24A>GA |
| H5 | c.292+28T>GT | WT | WT | WT | WT | WT | WT | WT | WT |
| H6 | NA | WT | c.490+40T>CT | WT | c.685-9T>CT | WT | c.1012G>AG p.Val338Ile | na | WT |
| DHL10 | c.292+28T>GT | WT | WT | WT | WT | WT | WT | WT | c.1307-24A>GA |
| DHL16 | WT | WT | WT | WT | WT | WT | WT | WT | WT |
| DHL2 | c.164G>AG,p.Arg55Gln | c.293-18A>GA | WT | WT | c.685-9T>CT | WT | c.1012G>GA p.Val338Ile | WT | c.1307-24A>GA |
| DHL4 | WT | c.293-18A>G | WT | WT | c.685-9T>C | WT | c.1082C>TC p.Ala361Val | WT | WT |
| DHL5 | c.292+28T>GT | c.293-18A>G | WT | WT | c.685-9T>C | WT | c.1082C>TC p.Ala361Val | c.1306+68T>CT | c.1307-24A>G |
| DHL6 | c.164G>AG,p.Arg55Gln c.292+28T>GT | c.293-18A>G | c.490+40T>CT | WT | c.685-9T>C | WT | c.1082C>TC p.Ala361Val | c.1262G>AG, p.Arg421His | c.1307-24A>GA |
| DHL7 | WT | WT | WT | WT | WT | WT | WT | WT | WT |
| DHL8 | c.164G>AG,p.Arg55Gln c.292+28T>GT | c.293-18A>G | c.490+40T>CT | c.622G>AG, p.Ala208Thr | c.685-9T>CT | WT | WT | c.1306+68T>CT | c.1307-24A>GA |
| Farage | WT | WT | WT | WT | WT | WT | WT | WT | WT |
| HBL-1 | c.292+28T>G | c.293-18A>G | WT | WT | c.685-9T>C | WT | c.1082C>TC p.Ala361Val | WT | WT |
| HT | WT | WT | WT | WT | WT | WT | WT | WT | WT |
| K1106P | c.164G>AG,p.Arg55Gln | WT | WT | WT | WT | WT | WT | WT | WT |
| K422 | c.164G>AG,p.Arg55Gln | c.293-18A>GA | WT | WT | c.685-9T>CT | WT | c.1082C>TC p.Ala361Val | c.1306+68T>CT | c.1307-24A>GA |
| Ly1 | c.164G>AG,p.Arg55Gln | WT | c.490+40T>CT | WT | WT | WT | WT | c.1306+68T>CT | c.1307-24A>GA |
| Ly10 | c.164G>AG,p.Arg55Gln c.292+28T>GT | c.293-18A>GA | c.490+40T>CT | WT | c.685-9T>CT | WT | WT | c.1306+68T>CT | c.1307-24A>G |
| Ly18 | WT | c.293-18A>GA | WT | WT | c.685-9T>CT | WT | WT | WT | WT |
| Ly19 | WT | WT | WT | WT | WT | WT | WT | WT | WT |
| Ly3 | c.164G>AG,p.Arg55Gln c.292+28T>GT | c.293-18A>GA | c.490+40T>CT | WT | c.685-9T>CT | WT | WT | c.1306+68T>CT | c.1307-24A>G |
| Ly4 | WT | WT | WT | WT | WT | WT | WT | WT | WT |
| Ly7 | c.1-91 C>GC c.292+28T>GT | c.293-18A>G | c.490+40T>C | WT | c.685-9T>C | WT | c.1012G>A p.Val338Ile c.1107T>CT; p.Asp369Asp | c.1276G>AG,p.Ala426Thr c.1306+68T>CT | c.1307-24A>G |
| Ly8 | c.292+28T>GT | c.293-18A>GA | WT | WT | c.685-9T>CT | WT | c.1082C>TC p.Ala361Val | WT | c.1307-24A>GA |
| NU-DHL1 | c.292+28T>GT | c.293-18A>GA | WT | WT | c.685-9T>CT | WT | c.1082C>TC p.Ala361Val | WT | WT |
| NU-DUL1 | c.164G>AG,p.Arg55Gln | c.293-18A>GA | WT | WT | c.685-9T>CT | WT | c.1012G>A p.Val338Ile | c.1306+68T>CT | c.1307-24A>GA |
| Pfeiffer | WT | WT | c.490+40T>CT | WT | WT | WT | WT | WT | WT |
| RC-K8 | c.292+28T>GT | c.293-18A>GA | WT | WT | c.685-9T>CT | WT | c.1082C>TC p.Ala361Val | WT | c.1307-24A>GA |
| Toledo | c.164G>AG,p.Arg55Gln c.292+28T>GT | c.293-18A>G | c.490+40T>CT | WT | c.685-9T>CT | WT | WT | c.1306+68T>CT | c.1307-24A>GA |
| U2932 | c.164G>AG,p.Arg55Gln c.292+28T>G | c.293-18A>G | c.490+40T>CT | WT | c.685-9T>C | WT | c.1012G>A p.Val338Ile | c.1306+68T>C | WT |
| USC-DHL1 | c.164G>AG,p.Arg55Gln c.292+28T>GT | WT | WT | WT | WT | WT | WT | WT | WT |
| WSU-NHL | c.164G>AG,p.Arg55Gln c.292+28T>GT | WT | c.392delG; Gly131fsX21 | WT | WT | WT | c.1064G>AG p.Gly355Ser | WT | c.1307-24A>GA |

rs62191976: c.1-91 C>GC= 2.7% (4%)
rs4675887: c.43C>CG, p.Arg15Gly= 2% (3.8%)
rs77940364: c.164G>AG p.Arg55Gln= 46.6% (21.7%)
rs62191977: c.292+28T>GT= 77% (73%)

rs4073889: c.293-18A>GA=61.4% (5
rs145731647: c.293-23A>TA=1.3% (

TMP-ESP-2-24261803: c.351-46G>AG= 0.6% (0.02 - 0.04)
rs78147778: c.490+40T>CT= 39.1% (22%)

rs4234097: c.685-9T>CT= 68.2% (52.7%)
TMP-ESP-2-242684213: c.774G>AG, p.Ser258Ser= 0.6% (0.0
rs180698643: c.853+48G>AG= 0.6% (0.001%)

rs1106639: c.1012G>AG p.Val338Ile= 31% (21%)
rs139321130: c.1063G>AG,p.Gly355Ser= 0.6% (0.001%)
rs1105273:c.1082C>TC p.Ala361Val= 28.3% (21.4%)
rs141343442: c.1107T>CT,p.Asp369Asp= 6% (2.8%)

rs139611034: c.1262G>A, p.Arg421His= 0.6% (0.01
TMP-ESP-2-242695395: c.1272G>AG p.Pro424Pro=
rs146578303: c.1276G>AG,p.Ala426Thr= 2% (<0.01%)
rs62192024: c.1306+68T>CT= 37.1% (33.7%)

rs6756901: c.1307-24A>GA= 67.5% (62.1%)
rs143940595: c.1377C>AC p.Pro459Pro= 6% (0.01%)

**Supplementary Table 2 - Copy number at the D2HGDH locus**

| | Q-PCR Upstream #1 | | Q-PCR Upstream #2 | | MLPA Ex2 | | Q-PCR Ex3 | | Q-PCR Ex4 | | Q-PCR Ex5 | | MLPA Ex6 | | MLPA Ex7 | | Q-PCR Ex8 | | MLPA Ex9 | | MLPA Ex10 | |
|---|---|---|---|---|---|---|---|---|---|---|---|---|---|---|---|---|---|---|---|---|---|---|
| Sample ID | Mean | Std-dev | Mean | Std-dev | Mean | Std-dev | Mean | Std-dev | Mean | Std-dev | Mean | Std-dev | Mean | Std-dev | Mean | Std-dev | Mean | Std-dev | Mean | Std-dev | Mean | Std-dev |
| A01 | 0.78 | n/a | 0.80 | n/a | 0.85 | 0.18 | 0.88 | n/a | 1.21 | n/a | 1.02 | n/a | 0.92 | 0.16 | 1.10 | 0.69 | 1.05 | n/a | 1.81 | 1.02 | 0.94 | 0.55 |
| B01 | 0.80 | 0.11 | 0.77 | 0.08 | 1.44 | 0.16 | 0.76 | 0.07 | 1.06 | 0.12 | 1.08 | 0.11 | 1.50 | 0.21 | 1.50 | 0.14 | 0.97 | 0.40 | 1.22 | 0.24 | 1.00 | 0.31 |
| C01 | 1.01 | 0.32 | 0.80 | 0.15 | 0.85 | 0.16 | 0.99 | 0.29 | 0.77 | 0.20 | 0.71 | 0.13 | 1.00 | 0.20 | 0.86 | 0.14 | 0.94 | 0.22 | 1.01 | 0.24 | 0.89 | 0.31 |
| D01 | 0.94 | 0.15 | 0.84 | 0.09 | 0.75 | 0.08 | 1.16 | 0.07 | 0.86 | 0.12 | 0.89 | 0.07 | 0.79 | 0.16 | 1.08 | 0.58 | 1.13 | 0.20 | 1.84 | 0.85 | 1.29 | 0.20 |
| E01 | 1.07 | 0.30 | 0.76 | 0.05 | 1.00 | 0.16 | 0.99 | 0.27 | 0.82 | 0.01 | 1.13 | 0.13 | 1.15 | 0.20 | 1.03 | 0.14 | 1.42 | 0.04 | 0.95 | 0.24 | 1.02 | 0.31 |
| F01 | 1.16 | 0.05 | 0.68 | 0.03 | 1.00 | 0.16 | 0.86 | 0.14 | 0.79 | 0.03 | 0.90 | 0.26 | 0.97 | 0.20 | 0.94 | 0.14 | 1.31 | 0.16 | 0.86 | 0.24 | 1.14 | 0.31 |
| A02 | 1.08 | 0.27 | 0.85 | 0.11 | 0.99 | 0.06 | 1.09 | 0.35 | 1.14 | 0.15 | 1.06 | 0.14 | 1.06 | 0.15 | 0.73 | 0.58 | 1.19 | 0.20 | 0.68 | 0.84 | 1.02 | 0.18 |
| D02 | 1.00 | 0.21 | 0.46 | 0.11 | 1.00 | 0.16 | 1.51 | 0.01 | 0.69 | 0.23 | 0.95 | 0.33 | 1.05 | 0.20 | 0.92 | 0.14 | 0.85 | n/a | 0.83 | 0.24 | 0.98 | 0.31 |
| H02 | 1.04 | 0.21 | 0.70 | 0.07 | 0.97 | 0.16 | 1.08 | 0.04 | 0.80 | 0.04 | 0.86 | 0.27 | 0.95 | 0.20 | 0.96 | 0.14 | 1.14 | 0.24 | 0.85 | 0.24 | 0.96 | 0.31 |
| B03 | 0.96 | 0.00 | 0.84 | 0.01 | 1.12 | 0.02 | 0.97 | 0.06 | 1.00 | 0.03 | 0.82 | 0.01 | 1.15 | 0.03 | 0.85 | 0.02 | 1.09 | n/a | 1.28 | 0.08 | 1.05 | 0.19 |
| D03 | 0.91 | 0.09 | 0.67 | n/a | 0.86 | 0.03 | 0.85 | 0.07 | 0.72 | 0.05 | 0.75 | 0.05 | 1.21 | 0.05 | 0.99 | 0.04 | 0.84 | 0.06 | 1.38 | 0.09 | 0.99 | 0.20 |
| E03 | 1.21 | 0.16 | 0.95 | 0.10 | 1.12 | 0.05 | 1.20 | 0.11 | 0.82 | 0.08 | 0.88 | 0.48 | 1.36 | 0.21 | 0.91 | 0.48 | 1.10 | 0.10 | 0.88 | 0.85 | 0.97 | 0.31 |
| F03 | 1.11 | 0.08 | 0.89 | 0.11 | 1.12 | 0.07 | 1.25 | n/a | 0.94 | 0.10 | 1.00 | 0.08 | 1.46 | 0.22 | 0.99 | 0.49 | 1.29 | 0.10 | 0.98 | 0.85 | 0.96 | 0.31 |
| A04 | 1.17 | 0.09 | 0.93 | 0.07 | 1.52 | 0.22 | 1.16 | 0.13 | 0.80 | 0.07 | 0.94 | 0.09 | 1.15 | 0.18 | 1.59 | 0.70 | 1.15 | 0.09 | 2.08 | 1.03 | 1.02 | 0.55 |
| C04 | 0.86 | 0.13 | 0.71 | 0.03 | 0.78 | 0.03 | 0.79 | 0.12 | 1.05 | 0.11 | 0.81 | 0.28 | 1.34 | 0.17 | 0.87 | 0.48 | 1.10 | n/a | 1.05 | 0.69 | 1.16 | 0.21 |
| D04 | 1.08 | 0.15 | 0.84 | 0.10 | 1.09 | 0.11 | 1.27 | 0.83 | 1.56 | 1.33 | 0.90 | 0.11 | 1.25 | 0.23 | 0.75 | 0.24 | 1.29 | 0.19 | 0.89 | 0.45 | 1.03 | 0.03 |
| G04 | 0.97 | 0.14 | 0.69 | 0.03 | 1.14 | 0.03 | 1.21 | 0.02 | 0.91 | 0.29 | 0.82 | n/a | 1.14 | 0.17 | 0.76 | 0.48 | 0.83 | 0.01 | 0.76 | 0.69 | 1.02 | 0.21 |
| F04 | 1.63 | 0.34 | 0.93 | 0.20 | 0.99 | 0.10 | 1.38 | 0.29 | 1.18 | 0.29 | 1.03 | 0.22 | 1.26 | 0.14 | 0.84 | 0.42 | 1.08 | 0.29 | 0.95 | 0.63 | 1.10 | 0.21 |
| H04 | 0.98 | 0.10 | 0.96 | 0.06 | 0.75 | 0.04 | 0.92 | 0.14 | 0.70 | 0.13 | 0.92 | 0.06 | 1.06 | 0.06 | 0.85 | 0.04 | 0.91 | 0.08 | 1.28 | 0.10 | 0.92 | 0.20 |
| A05 | 1.02 | 0.02 | 1.05 | 0.03 | 0.99 | 0.05 | 1.09 | 0.10 | 1.43 | 0.41 | 1.08 | 0.02 | 1.34 | 0.07 | 1.08 | 0.06 | 1.10 | 0.01 | 1.60 | 0.12 | 1.02 | 0.20 |
| B05 | 1.26 | 0.29 | 1.13 | n/a | 1.40 | 0.11 | 1.01 | 0.23 | 0.74 | 0.78 | 0.90 | 0.02 | 1.27 | 0.23 | 0.75 | 0.24 | 0.84 | 0.05 | 0.81 | 0.45 | 1.19 | 0.03 |
| C05 | 0.99 | 0.04 | 0.80 | 0.04 | 0.90 | 0.12 | 1.25 | 0.16 | 0.96 | 0.09 | 0.96 | 0.05 | 0.84 | 0.23 | 1.16 | 0.24 | 1.26 | 0.06 | 1.70 | 0.46 | 0.85 | 0.05 |
| D05 | 1.55 | 0.13 | 1.03 | 0.10 | 0.90 | 0.30 | 1.74 | 0.23 | 0.91 | 0.11 | 0.97 | 0.09 | 1.00 | 0.14 | 0.86 | 0.34 | 1.10 | 0.13 | 0.65 | 0.73 | 0.90 | 0.03 |
| F05 | 0.92 | n/a | 0.71 | 0.09 | 0.89 | 0.11 | 1.13 | 0.11 | 0.91 | 0.18 | 0.88 | 0.04 | 1.38 | 0.24 | 0.82 | 0.24 | 1.18 | 0.03 | 1.00 | 0.46 | 0.88 | 0.04 |
| G05 | 1.03 | 0.19 | 0.80 | 0.05 | 0.92 | 0.02 | 1.35 | 0.17 | 0.99 | 0.11 | 1.06 | 0.09 | 1.07 | 0.03 | 0.93 | 0.02 | 1.24 | 0.10 | 1.21 | 0.08 | 1.11 | 0.18 |
| H05 | 0.82 | 0.19 | 0.75 | 0.03 | 1.01 | 0.11 | 1.25 | 0.16 | 1.23 | 0.25 | 1.18 | 0.04 | 1.32 | 0.24 | 0.90 | 0.24 | 1.52 | 0.13 | 1.11 | 0.46 | 1.08 | 0.05 |
| SU-DHL4 | 0.99 | 0.08 | 0.79 | 0.07 | 1.04 | 0.03 | 1.30 | 0.07 | 1.03 | 0.03 | 1.01 | 0.07 | 1.04 | 0.12 | 1.02 | 0.09 | 1.09 | 0.04 | 1.12 | 0.06 | 0.90 | 0.03 |
| SU-DHL5 | 0.87 | 0.18 | 0.82 | 0.01 | 1.04 | 0.03 | 1.17 | 0.04 | 1.05 | 0.02 | 0.84 | 0.15 | 0.92 | 0.12 | 0.91 | 0.09 | 1.00 | 0.16 | 0.98 | 0.06 | 0.93 | 0.03 |
| SU-DHL7 | 0.93 | 0.08 | 0.85 | 0.01 | 1.43 | 0.08 | 1.05 | 0.01 | 1.03 | 0.12 | 0.97 | 0.03 | 1.26 | 0.14 | 1.31 | 0.11 | 1.09 | 0.05 | 1.29 | 0.09 | 1.21 | 0.07 |
| SU-DHL8 | 1.06 | 0.06 | 1.01 | 0.03 | 1.06 | 0.12 | 0.74 | 0.05 | 0.80 | 0.02 | 0.80 | 0.05 | 0.72 | 0.15 | 2.54 | 0.30 | 0.88 | 0.02 | 3.26 | 0.37 | 1.29 | 0.15 |
| SU-DHL9 | 0.94 | 0.06 | 0.89 | 0.04 | 1.05 | 0.04 | 1.09 | 0.15 | 1.50 | 0.12 | 1.33 | 0.06 | 1.02 | 0.20 | 0.74 | 0.48 | 1.39 | 0.06 | 0.67 | 0.85 | 1.00 | 0.31 |
| SU-DHL10 | 0.86 | 0.06 | 0.86 | 0.03 | 1.01 | 0.61 | 0.96 | 0.23 | 1.56 | 0.19 | 1.32 | 0.05 | 1.09 | 0.55 | 1.14 | 0.58 | 1.37 | 0.07 | 1.05 | 0.50 | 1.08 | 0.47 |
| SU-DHL-16 | 0.99 | 0.07 | 1.00 | 0.06 | 1.18 | 0.04 | 1.22 | 0.16 | 1.31 | 0.12 | 1.09 | 0.07 | 1.35 | 0.20 | 0.99 | 0.48 | 1.34 | 0.13 | 0.81 | 0.85 | 0.92 | 0.31 |
| Farage | 1.01 | 0.09 | 1.05 | 0.19 | 0.88 | 0.10 | 1.06 | 0.21 | 1.59 | 0.13 | 1.42 | 0.11 | 0.68 | 0.14 | 1.66 | 0.43 | 1.48 | 0.12 | 1.85 | 0.64 | 1.23 | 0.22 |
| HBL1 | 0.83 | 0.05 | 0.83 | 0.03 | 1.36 | 0.06 | 1.15 | 0.27 | 1.02 | 0.02 | 1.03 | 0.04 | 1.11 | 0.13 | 1.18 | 0.10 | 1.03 | 0.04 | 1.16 | 0.08 | 0.94 | 0.04 |
| HT | 0.90 | 0.10 | 0.76 | 0.05 | 1.01 | 0.05 | 0.92 | 0.10 | 1.00 | 0.04 | 1.01 | 0.06 | 1.00 | 0.13 | 1.03 | 0.10 | 1.02 | 0.05 | 1.11 | 0.08 | 1.04 | 0.06 |
| Karpas 422 | 0.89 | 0.08 | 0.83 | 0.05 | 1.01 | 0.01 | 1.28 | 0.19 | 1.20 | 0.06 | 1.20 | 0.07 | 0.99 | 0.12 | 0.99 | 0.08 | 1.12 | 0.05 | 1.01 | 0.06 | 0.88 | 0.01 |
| Ly1 | 0.87 | 0.03 | 0.81 | 0.00 | 1.03 | 0.02 | 1.13 | 0.03 | 1.08 | 0.13 | 1.07 | 0.02 | 1.09 | 0.12 | 1.17 | 0.09 | 1.04 | 0.02 | 1.09 | 0.06 | 0.73 | 0.02 |
| Ly7 | 0.87 | 0.12 | 0.85 | 0.02 | 1.01 | 0.04 | 0.83 | 0.21 | 0.75 | 0.01 | 0.82 | 0.03 | 0.88 | 0.13 | 0.90 | 0.09 | 0.84 | 0.01 | 0.95 | 0.07 | 0.89 | 0.04 |
| Ly8 | 0.87 | 0.10 | 1.33 | 1.13 | 1.36 | 0.14 | 1.01 | 0.13 | 0.97 | 0.06 | 0.91 | 0.11 | 0.84 | 0.15 | 2.93 | 0.31 | 0.95 | 0.06 | 3.11 | 0.33 | 0.80 | 0.08 |
| Ly10 | 1.03 | 0.31 | 0.87 | 0.11 | 1.19 | 0.02 | 1.03 | 0.15 | 1.09 | 0.17 | 1.01 | 0.11 | 0.70 | 0.14 | 2.83 | 0.27 | 0.94 | 0.10 | 2.99 | 0.28 | 0.90 | 0.08 |
| Ly18 | 0.70 | 0.12 | 0.81 | 0.07 | 1.02 | 0.02 | 1.26 | 0.21 | 0.97 | 0.14 | 1.05 | 0.08 | 0.93 | 0.12 | 0.98 | 0.09 | 0.98 | 0.08 | 1.05 | 0.06 | 1.09 | 0.02 |
| Ly19 | 0.81 | 0.02 | 0.94 | 0.02 | 0.77 | 0.02 | 1.27 | 0.08 | 0.95 | 0.30 | 1.09 | 0.02 | 0.78 | 0.12 | 0.75 | 0.09 | 0.95 | 0.03 | 0.85 | 0.06 | 1.02 | 0.03 |
| Pfeiffer | 0.93 | 0.13 | 0.89 | 0.05 | 1.00 | 0.01 | 0.96 | 0.21 | 1.09 | 0.14 | 1.10 | 0.07 | 1.03 | 0.12 | 1.00 | 0.08 | 1.14 | 0.06 | 1.08 | 0.06 | 1.01 | 0.01 |
| Toledo | 0.95 | 0.03 | 1.12 | 0.48 | 1.00 | 0.02 | 1.05 | 0.07 | 0.97 | 0.07 | 0.95 | 0.23 | 1.09 | 0.03 | 1.04 | 0.02 | 1.05 | 0.34 | 1.14 | 0.08 | 1.13 | 0.18 |
| U2932 | 0.52 | 0.07 | 0.33 | n/a | 0.50 | 0.02 | 0.78 | 0.16 | 0.45 | 0.05 | 0.41 | n/a | 0.51 | 0.03 | 0.51 | 0.02 | 0.54 | 0.07 | 0.62 | 0.08 | 0.53 | 0.18 |
| WSU-NHL | 1.13 | 0.24 | 1.01 | 0.27 | 0.89 | 0.11 | 1.30 | 0.30 | 0.95 | 0.16 | 1.10 | 0.20 | 1.06 | 0.23 | 0.76 | 0.24 | 1.12 | 0.49 | 0.70 | 0.46 | 0.97 | 0.04 |
| USC-DHL1 | 1.08 | 0.14 | 1.12 | 0.01 | 0.99 | 0.02 | 1.04 | 0.27 | 0.91 | 0.06 | 0.91 | 0.07 | 1.17 | 0.03 | 1.06 | 0.02 | 0.88 | 0.02 | 1.16 | 0.08 | 0.91 | 0.18 |
| NU-DUL1 | 0.73 | n/a | 1.06 | n/a | 1.00 | 0.61 | 1.52 | n/a | n/a | n/a | 1.40 | n/a | 1.01 | 0.55 | 1.03 | 0.58 | 1.26 | n/a | 0.98 | 0.50 | 0.90 | 0.48 |
| NU-DH-1 | 1.78 | 0.36 | 1.12 | 0.23 | 1.09 | 0.02 | 2.42 | 0.71 | 1.08 | 0.33 | 1.08 | 0.23 | 1.15 | 0.03 | 1.11 | 0.02 | 1.38 | 0.29 | 1.17 | 0.08 | 1.19 | 0.18 |

The cell line U2932 showed a pattern of putative single copy loss across the entire D2HDGH locus and the upstream region (4 Mbp from D2HGDH's transcription start site).
This finding was in agreement with an earlier report on the karyotype of this cell line (Leukemia and Lymphoma, 2002; 43: 2179 -2189).
The threshold for positive calls (copy number loss) was 0.7 or, when only one exon appeared to be targeted, 0.65.

**Supplementary Table 3 - α-KG abundance in multiple D2HGDH models in [U-$^{13}$C]-glutamine labeling assay**

| Sample ID | unspiked % enriched | spiked % enriched | fold dilution | nmoles in solution | nmoles of α-KG/mg of protein | Relative abundance |
|---|---|---|---|---|---|---|
| **MSCV** | 0.6031 | 0.3972 | 1.5184 | 48.2273 | 0.1901 | 0.46 |
| **D2HGDH -WT** | 0.5584 | 0.4568 | 1.2224 | 112.4016 | 0.4107 | 1.00 |
| **D2HGDH - R212W** | 0.5947 | 0.4429 | 1.3427 | 72.9414 | 0.3004 | 0.73 |
| **D2HGDH -A426T** | 0.5761 | 0.3829 | 1.5046 | 49.5471 | 0.1938 | 0.47 |

4h

| | | | | | | |
|---|---|---|---|---|---|---|
| **MSCV** | 0.6205 | 0.4651 | 1.3341 | 74.8230 | 0.2872 | 0.75 |
| **D2HGDH -WT** | 0.6045 | 0.4952 | 1.2207 | 113.2662 | 0.3850 | 1.00 |
| **D2HGDH - R212W** | 0.6112 | 0.4219 | 1.4487 | 55.7184 | 0.1898 | 0.49 |
| **D2HGDH -A426T** | 0.5941 | 0.3878 | 1.5320 | 46.9947 | 0.1809 | 0.47 |

8h

## Supplementary Table 4 – Summary of IDH $V_{max}$ and $K_m$ in multiple D2HGDH genetic models

Stable expression – gain-of-function

| Cell ID | Total cell lysate - HEK-293 | |
|---|---|---|
| | $V_{max}$ (mean ± SD) | $K_m$ (mean ± SD) |
| MSCV (n=3) | 29.7 (±0.51) | 0.69 (±0.06) |
| D2HGDH – WT (n=3) | 33.9 (±0.74) | 0.56 (±0.06) |
| D2HGDH – G131X (n=3) | 26.9 (±0.55) | 0.76 (±0.08) |
| D2HGDH – A208T (n=3) | 27.4 (±0.50) | 0.82 (±0.07) |
| D2HGDH – R212W (n=3) | 29.8 (±0.57) | 0.78 (±0.07) |
| D2HGDH – R421H (n=3) | 26.1 (±0.50) | 0.66 (±0.06) |
| D2HGDH – A426T (n=3) | 29.2 (±0.58) | 0.62 (±0.06) |

Stable expression – loss-of-function

| Cell ID | Total cell lysate – HEK-293 KD | |
|---|---|---|
| | $V_{max}$ (mean ± SD) | $K_m$ (mean ± SD) |
| si-control (n=3) | 30.4 (±0.38) | 0.57 (±0.03) |
| si-D2HGDH#3 (n=3) | 25.0 (±0.29) | 0.59 (±0.03) |
| si-D2HGDH#5 (n=3) | 25.9 (±0.28) | 0.52 (±0.03) |

Transient expression – gain-of-function

| Cell ID | Total cell lysate HEK-293 – transient transfection | |
|---|---|---|
| | $V_{max}$ (mean ± SD) | $K_m$ (mean ± SD) |
| MSCV 0.5µg (n=3) | 31.7 (±0.53) | 1.33 (±0.10) |
| MSCV 0.75µg (n=3) | 31.5 (±0.56) | 1.31 (±0.11) |
| MSCV 1.0µg (n=3) | 31.7 (±0.53) | 1.33 (±0.10) |
| D2 – WT 0.5µg (n=3) | 33.5 (±0.34) | 0.96 (±0.04) |
| D2 – WT 0.75µg (n=3) | 36.4 (±0.49) | 1.12 (±0.07) |
| D2 – WT 1.0µg (n=3) | 38.1 (±0.39) | 1.29 (±0.06) |

Transient expression – loss-of-function

| Cell ID | Total cell lysate – OCY-Ly8 KD | |
|---|---|---|
| | $V_{max}$ (mean ± SD) | $K_m$ (mean ± SD) |
| si-control (n=3) | 48.7 (±1.29) | 2.22 (±0.25) |
| si-D2HGDH#3 (n=3) | 40.2 (±0.69) | 2.08 (±0.15) |
| si-D2HGDH#5 (n=3) | 41.9 (±0.93) | 2.20 (±0.21) |

Transient expression – loss-of-function

| Cell ID | Total cell lysate – Ramos KD | |
|---|---|---|
| | $V_{max}$ (mean ± SD) | $K_m$ (mean ± SD) |
| si-control (n=3) | 25.6 (±0.20) | 1.06 (±0.04) |
| si-D2HGDH#3 (n=3) | 21.6 (±0.25) | 1.05 (±0.06) |
| si-D2HGDH#5 (n=3) | 19.5 (±0.24) | 0.89 (±0.05) |

Stable expression – loss-of-function

| | Subcellular fractions – HEK-293 | | | |
|---|---|---|---|---|
| Cell ID | Mitochondria | | Cytosol | |
| | $V_{max}$ (mean ± SD) | $K_m$ (mean ± SD) | $V_{max}$ (mean ± SD) | $K_m$ (mean ± SD) |
| si-control (n=3) | 84.9 (±1.42) | 1.01 (±0.07) | 17.9 (±0.23) | 0.67 (±0.04) |
| si-D2HGDH#3 (n=3) | 55.3 (±1.20) | 1.10 (±0.10) | 17.6 (±0.24) | 0.72 (±0.04) |
| si-D2HGDH#5 (n=3) | 54.4 (±0.89) | 0.88 (±0.06) | 17.5 (±0.21) | 0.80(±0.04) |

Transient expression – gain-of-function

| | Subcellular fractions – HEK-293 | | | |
|---|---|---|---|---|
| Cell ID | Mitochondria | | Cytosol | |
| | $V_{max}$ (mean ± SD) | $K_m$ (mean ± SD) | $V_{max}$ (mean ± SD) | $K_m$ (mean ± SD) |
| MSCV (n=3) | 58.8 (±1.18) | 1.01 (±0.09) | 17.6 (±0.26) | 0.83 (±0.05) |
| D2HGDH – WT (n=3) | 84.3 (±1.66) | 1.06 (±0.09) | 17.7 (±0.26) | 0.91 (±0.06) |

**Supplementary Table 5. IDH activity assay in total cell lysates**

**Stable expression**

| **MSCV** | #1 | | | #2 | | | #3 | | |
|---|---|---|---|---|---|---|---|---|---|
| d-iso(uM) | A340nm/min | umol/min/mg | Vmax | A340nm/min | umol/min/mg | Vmax | A340nm/min | umol/min/mg | Vmax |
| 0.25 | 0.0027 | 5.71 | 29.14 | 0.0022 | 4.65 | 30.02 | 0.0018 | 3.81 | 29.88 |
| 0.5 | 0.0067 | 14.17 | | 0.0065 | 13.75 | | 0.0064 | 13.54 | |
| 1 | 0.0087 | 18.40 | Km | 0.0085 | 17.98 | Km | 0.0096 | 20.31 | Km |
| 2.5 | 0.0107 | 22.63 | 0.65 | 0.0114 | 24.12 | 0.74 | 0.0112 | 23.69 | 0.69 |
| 5 | 0.0120 | 25.39 | | 0.0118 | 24.96 | | 0.0111 | 23.48 | |
| 10 | 0.0135 | 28.56 | | 0.0142 | 30.04 | | 0.0140 | 29.62 | |
| 15 | 0.0130 | 27.50 | | 0.0135 | 28.56 | | 0.0140 | 29.62 | |
| 25 | 0.0133 | 28.14 | | 0.0135 | 28.56 | | 0.0135 | 28.56 | |
| 50 | 0.0135 | 28.56 | | 0.0141 | 29.83 | | 0.0135 | 28.56 | |

| **D2HGDH WT** | #1 | | | #2 | | | #3 | | |
|---|---|---|---|---|---|---|---|---|---|
| d-iso(uM) | A340nm/min | umol/min/mg | Vmax | A340nm/min | umol/min/mg | Vmax | A340nm/min | umol/min/mg | Vmax |
| 0.25 | 0.0029 | 6.48 | 33.34 | 0.0025 | 5.58 | 34.01 | 0.0035 | 7.82 | 34.56 |
| 0.5 | 0.0072 | 16.08 | | 0.0075 | 16.75 | | 0.0085 | 18.98 | |
| 1 | 0.0097 | 21.66 | Km | 0.0094 | 20.99 | Km | 0.0137 | 30.59 | Km |
| 2.5 | 0.0126 | 28.14 | 0.61 | 0.0124 | 27.69 | 0.67 | 0.0124 | 27.69 | 0.43 |
| 5 | 0.0125 | 27.91 | | 0.0126 | 28.14 | | 0.0130 | 29.03 | |
| 10 | 0.0144 | 32.15 | | 0.0145 | 32.38 | | 0.0152 | 33.94 | |
| 15 | 0.0145 | 32.38 | | 0.0144 | 32.15 | | 0.0150 | 33.49 | |
| 25 | 0.0144 | 32.15 | | 0.0145 | 32.38 | | 0.0155 | 34.61 | |
| 50 | 0.0145 | 32.38 | | 0.0155 | 34.61 | | 0.0152 | 33.94 | |

| **D2HGDH G131X** | #1 | | | #2 | | | #3 | | |
|---|---|---|---|---|---|---|---|---|---|
| d-iso(uM) | A340nm/min | umol/min/mg | Vmax | A340nm/min | umol/min/mg | Vmax | A340nm/min | umol/min/mg | Vmax |
| 0.25 | 0.0014 | 2.96 | 27.04 | 0.0022 | 4.65 | 27.04 | 0.0025 | 5.29 | 26.72 |
| 0.5 | 0.0030 | 6.35 | | 0.0040 | 8.46 | | 0.0062 | 13.12 | |
| 1 | 0.0084 | 17.77 | Km | 0.0080 | 16.92 | Km | 0.0084 | 17.77 | Km |
| 2.5 | 0.0107 | 22.63 | 0.88 | 0.0099 | 20.94 | 0.83 | 0.0107 | 22.63 | 0.6 |
| 5 | 0.0107 | 22.63 | | 0.0109 | 23.06 | | 0.0105 | 22.21 | |
| 10 | 0.0117 | 24.75 | | 0.0122 | 25.81 | | 0.0122 | 25.81 | |
| 15 | 0.0120 | 25.39 | | 0.0122 | 25.81 | | 0.0122 | 25.81 | |
| 25 | 0.0122 | 25.81 | | 0.0120 | 25.39 | | 0.0121 | 25.60 | |
| 50 | 0.0120 | 25.39 | | 0.0122 | 25.81 | | 0.0125 | 26.44 | |

| **D2HGDH A208T** | #1 | | | #2 | | | #3 | | |
|---|---|---|---|---|---|---|---|---|---|
| d-iso(uM) | A340nm/min | umol/min/mg | Vmax | A340nm/min | umol/min/mg | Vmax | A340nm/min | umol/min/mg | Vmax |
| 0.25 | 0.0020 | 5.02 | 27.5 | 0.0018 | 4.52 | 27.59 | 0.0022 | 5.53 | 27.45 |
| 0.5 | 0.0040 | 10.05 | | 0.0045 | 11.30 | | 0.0055 | 13.82 | |
| 1 | 0.0058 | 14.57 | Km | 0.0063 | 15.83 | Km | 0.0070 | 17.58 | Km |
| 2.5 | 0.0071 | 17.84 | 0.98 | 0.0073 | 18.34 | 0.87 | 0.0077 | 19.34 | 0.63 |
| 5 | 0.0092 | 23.11 | | 0.0090 | 22.61 | | 0.0103 | 25.87 | |
| 10 | 0.0100 | 25.12 | | 0.0106 | 26.63 | | 0.0111 | 27.88 | |
| 15 | 0.0111 | 27.88 | | 0.0105 | 26.38 | | 0.0100 | 25.12 | |
| 25 | 0.0100 | 25.12 | | 0.0105 | 26.38 | | 0.0106 | 26.63 | |
| 50 | 0.0107 | 26.88 | | 0.0108 | 27.13 | | 0.0105 | 26.38 | |

| **D2HGDH R212W** | #1 | | | #2 | | | #3 | | |
|---|---|---|---|---|---|---|---|---|---|
| d-iso(uM) | A340nm/min | umol/min/mg | Vmax | A340nm/min | umol/min/mg | Vmax | A340nm/min | umol/min/mg | Vmax |
| 0.25 | 0.0018 | 3.36 | 30.05 | 0.0025 | 4.67 | 29.94 | 0.0014 | 2.62 | 29.6 |
| 0.5 | 0.0068 | 12.71 | | 0.0058 | 10.84 | | 0.0079 | 14.77 | |
| 1 | 0.0096 | 17.95 | Km | 0.0096 | 17.95 | Km | 0.0104 | 19.44 | Km |
| 2.5 | 0.0126 | 23.55 | 0.79 | 0.0123 | 22.99 | 0.87 | 0.0123 | 22.99 | 0.7 |
| 5 | 0.0138 | 25.80 | | 0.0120 | 22.43 | | 0.0130 | 24.30 | |
| 10 | 0.0155 | 28.98 | | 0.0152 | 28.42 | | 0.0151 | 28.23 | |
| 15 | 0.0155 | 28.98 | | 0.0155 | 28.98 | | 0.0152 | 28.42 | |
| 25 | 0.0150 | 28.04 | | 0.0156 | 29.16 | | 0.0154 | 28.79 | |
| 50 | 0.0153 | 28.60 | | 0.0156 | 29.16 | | 0.0155 | 28.98 | |

| **D2HGDH R421H** | #1 | | | #2 | | | #3 | | |
|---|---|---|---|---|---|---|---|---|---|
| d-iso(uM) | A340nm/min | umol/min/mg | Vmax | A340nm/min | umol/min/mg | Vmax | A340nm/min | umol/min/mg | Vmax |
| 0.25 | 0.0020 | 4.02 | 26.24 | 0.0022 | 4.42 | 25.74 | 0.0025 | 5.02 | 26.35 |
| 0.5 | 0.0060 | 12.06 | | 0.0068 | 13.67 | | 0.0052 | 10.45 | |
| 1 | 0.0080 | 16.08 | Km | 0.0091 | 18.29 | Km | 0.0098 | 19.69 | Km |
| 2.5 | 0.0097 | 19.49 | 0.74 | 0.0096 | 19.29 | 0.58 | 0.0091 | 18.29 | 0.68 |
| 5 | 0.0111 | 22.31 | | 0.0111 | 22.31 | | 0.0111 | 22.31 | |
| 10 | 0.0123 | 24.72 | | 0.0126 | 25.32 | | 0.0130 | 26.13 | |
| 15 | 0.0130 | 26.13 | | 0.0125 | 25.12 | | 0.0125 | 25.12 | |
| 25 | 0.0126 | 25.32 | | 0.0125 | 25.12 | | 0.0130 | 26.13 | |
| 50 | 0.0125 | 25.12 | | 0.0124 | 24.92 | | 0.0126 | 25.32 | |

| **D2HGDH A426T** | #1 | | | #2 | | | #3 | | |
|---|---|---|---|---|---|---|---|---|---|
| d-iso(uM) | A340nm/min | umol/min/mg | Vmax | A340nm/min | umol/min/mg | Vmax | A340nm/min | umol/min/mg | Vmax |
| 0.25 | 0.0025 | 6.70 | 27.88 | 0.0021 | 5.63 | 29.36 | 0.0015 | 4.02 | 30.66 |
| 0.5 | 0.0063 | 16.88 | | 0.0042 | 11.25 | | 0.0048 | 12.86 | |
| 1 | 0.0073 | 19.56 | Km | 0.0066 | 17.68 | Km | 0.0079 | 21.17 | Km |
| 2.5 | 0.0087 | 23.31 | 0.46 | 0.0090 | 24.12 | 0.72 | 0.0087 | 23.31 | 0.69 |
| 5 | 0.0098 | 26.26 | | 0.0100 | 26.80 | | 0.0105 | 28.14 | |
| 10 | 0.0099 | 26.53 | | 0.0101 | 27.06 | | 0.0114 | 30.55 | |
| 15 | 0.0099 | 26.53 | | 0.0111 | 29.74 | | 0.0105 | 28.14 | |
| 25 | 0.0105 | 28.14 | | 0.0105 | 28.14 | | 0.0111 | 29.74 | |
| 50 | 0.0099 | 26.53 | | 0.0098 | 26.26 | | 0.0106 | 28.40 | |

Enzyme kinectis calculated using the Michaelis-Menten equation

**Transient Transfection**

| **MSCV (0.5ug)** | #1 | | | #2 | | | #3 | | |
|---|---|---|---|---|---|---|---|---|---|
| d-iso(uM) | A340nm/min | umol/min/mg | Vmax | A340nm/min | umol/min/mg | Vmax | A340nm/min | umol/min/mg | |
| 0.25 | 0.0012 | 4.95 | 32.56 | 0.0012 | 4.95 | 31.8 | 0.0013 | 5.36 | Vmax |
| 0.5 | 0.0026 | 10.72 | | 0.0027 | 11.13 | | 0.0026 | 10.72 | 30.89 |
| 1 | 0.0032 | 13.19 | Km | 0.0035 | 14.43 | Km | 0.0036 | 14.84 | |
| 2.5 | 0.0041 | 16.90 | 1.51 | 0.0041 | 16.90 | 1.34 | 0.0043 | 17.73 | Km |
| 5 | 0.0060 | 24.73 | | 0.0060 | 24.73 | | 0.0065 | 26.80 | 1.13 |
| 10 | 0.0073 | 30.09 | protein | 0.0072 | 29.68 | protein | 0.0074 | 30.51 | |
| 15 | 0.0074 | 30.51 | 39ug | 0.0073 | 30.09 | 39ug | 0.0070 | 28.86 | protein |
| 25 | 0.0075 | 30.92 | | 0.0074 | 30.51 | | 0.0070 | 28.86 | 39ug |
| 50 | 0.0074 | 30.51 | | 0.0073 | 30.09 | | 0.0070 | 28.86 | |

| **D2HGDH WT(0.5ug)** | #1 | | | #2 | | | #3 | | |
|---|---|---|---|---|---|---|---|---|---|
| d-iso(uM) | A340nm/min | umol/min/mg | Vmax | A340nm/min | umol/min/mg | Vmax | A340nm/min | umol/min/mg | |
| 0.25 | 0.0013 | 5.23 | 33.58 | 0.0013 | 5.23 | 33.71 | 0.0014 | 5.63 | Vmax |
| 0.5 | 0.0031 | 12.46 | | 0.0032 | 12.86 | | 0.0031 | 12.46 | 33.43 |
| 1 | 0.0040 | 16.08 | Km | 0.0042 | 16.88 | Km | 0.0045 | 18.09 | |
| 2.5 | 0.0058 | 23.31 | 1.02 | 0.0058 | 23.31 | 0.96 | 0.0059 | 23.71 | Km |
| 5 | 0.0071 | 28.54 | | 0.0075 | 30.14 | | 0.0075 | 30.14 | 0.9 |
| 10 | 0.0077 | 30.95 | protein | 0.0076 | 30.55 | protein | 0.0075 | 30.14 | |
| 15 | 0.0079 | 31.75 | 40ug | 0.0078 | 31.35 | 40ug | 0.0079 | 31.75 | protein |
| 25 | 0.0081 | 32.56 | | 0.0082 | 32.96 | | 0.0079 | 31.75 | 40ug |
| 50 | 0.0079 | 31.75 | | 0.0080 | 32.15 | | 0.0081 | 32.56 | |

| **MSCV(0.75)** | #1 | | | #2 | | | #3 | | |
|---|---|---|---|---|---|---|---|---|---|
| d-iso(uM) | A340nm/min | umol/min/mg | Vmax | A340nm/min | umol/min/mg | Vmax | A340nm/min | umol/min/mg | |
| 0.25 | 0.0012 | 4.95 | 31.87 | 0.0011 | 4.53 | 31.66 | 0.0012 | 4.95 | Vmax |
| 0.5 | 0.0026 | 10.72 | | 0.0026 | 10.72 | | 0.0025 | 10.31 | 31.09 |
| 1 | 0.0033 | 13.60 | Km | 0.0032 | 13.19 | Km | 0.0035 | 14.43 | |
| 2.5 | 0.0042 | 17.31 | 1.37 | 0.0042 | 17.31 | 1.36 | 0.0045 | 18.55 | Km |
| 5 | 0.0060 | 24.73 | | 0.0063 | 25.97 | | 0.0060 | 24.73 | 1.22 |
| 10 | 0.0074 | 30.51 | Protein | 0.0073 | 30.09 | protein | 0.0072 | 29.68 | |
| 15 | 0.0073 | 30.09 | 39ug | 0.0072 | 29.68 | 39ug | 0.0072 | 29.68 | protein |
| 25 | 0.0073 | 30.09 | | 0.0070 | 28.86 | | 0.0071 | 29.27 | 39ug |
| 50 | 0.0072 | 29.68 | | 0.0073 | 30.09 | | 0.0071 | 29.27 | |

| **D2HGDH WT(0.75ug)** | #1 | | | #2 | | | #3 | | |
|---|---|---|---|---|---|---|---|---|---|
| d-iso(uM) | A340nm/min | umol/min/mg | Vmax | A340nm/min | umol/min/mg | Vmax | A340nm/min | umol/min/mg | |
| 0.25 | 0.0015 | 5.36 | 36.83 | 0.0015 | 5.36 | 36.28 | 0.0016 | 5.72 | Vmax |
| 0.5 | 0.0033 | 11.79 | | 0.0035 | 12.50 | | 0.0038 | 13.58 | 36.24 |
| 1 | 0.0047 | 16.79 | Km | 0.0049 | 17.51 | Km | 0.0049 | 17.51 | |
| 2.5 | 0.0065 | 23.22 | 1.243 | 0.0064 | 22.87 | 1.12 | 0.0069 | 24.65 | Km |
| 5 | 0.0079 | 28.22 | | 0.0084 | 30.01 | | 0.0085 | 30.37 | 1.01 |
| 10 | 0.0098 | 35.01 | protein | 0.0097 | 34.66 | protein | 0.0097 | 34.66 | |
| 15 | 0.0099 | 35.37 | 45ug | 0.0097 | 34.66 | 45ug | 0.0098 | 35.01 | protein |
| 25 | 0.0099 | 35.37 | | 0.0096 | 34.30 | | 0.0098 | 35.01 | 45ug |
| 50 | 0.0095 | 33.94 | | 0.0095 | 33.94 | | 0.0093 | 33.23 | |

| **MSCV(1.0)** | #1 | | | #2 | | | #3 | | |
|---|---|---|---|---|---|---|---|---|---|
| d-iso(uM) | A340nm/min | umol/min/mg | Vmax | A340nm/min | umol/min/mg | Vmax | A340nm/min | umol/min/mg | |
| 0.25 | 0.0012 | 4.95 | 32.39 | 0.0011 | 4.53 | 31.82 | 0.0011 | 4.53 | Vmax |
| 0.5 | 0.0025 | 10.31 | | 0.0025 | 10.31 | | 0.0026 | 10.72 | 31.13 |
| 1 | 0.0032 | 13.19 | Km | 0.0033 | 13.60 | Km | 0.0034 | 14.02 | |
| 2.5 | 0.0041 | 16.90 | 1.49 | 0.0049 | 20.20 | 1.260 | 0.0043 | 17.73 | km |
| 5 | 0.0061 | 25.15 | | 0.0061 | 25.15 | | 0.0063 | 25.97 | 1.26 |
| 10 | 0.0073 | 30.09 | protein | 0.0075 | 30.92 | protein | 0.0071 | 29.27 | |
| 15 | 0.0073 | 30.09 | 39ug | 0.0072 | 29.68 | 39ug | 0.0070 | 28.86 | protein |
| 25 | 0.0075 | 30.92 | | 0.0070 | 28.86 | | 0.0070 | 28.86 | 39ug |
| 50 | 0.0073 | 30.09 | | 0.0074 | 30.51 | | 0.0073 | 30.09 | |

| **D2HGDH WT(1.0ug)** | #1 | | | #2 | | | #3 | | |
|---|---|---|---|---|---|---|---|---|---|
| d-iso(uM) | A340nm/min | umol/min/mg | Vmax | A340nm/min | umol/min/mg | Vmax | A340nm/min | umol/min/mg | |
| 0.25 | 0.0017 | 5.26 | 38.2 | 0.0018 | 5.57 | 38.49 | 0.0017 | 5.26 | Vmax |
| 0.5 | 0.0039 | 12.06 | | 0.0030 | 9.28 | | 0.0035 | 10.82 | 37.79 |
| 1 | 0.0056 | 17.31 | Km | 0.0052 | 16.08 | Km | 0.0053 | 16.39 | |
| 2.5 | 0.0079 | 24.42 | 1.23 | 0.0080 | 24.73 | 1.38 | 0.0081 | 25.04 | Km |
| 5 | 0.0098 | 30.30 | | 0.0099 | 30.61 | | 0.0098 | 30.30 | 1.26 |
| 10 | 0.0117 | 36.17 | protein | 0.0115 | 35.56 | protein | 0.0116 | 35.86 | |
| 15 | 0.0117 | 36.17 | 52ug | 0.0116 | 35.86 | 52ug | 0.0113 | 34.94 | protein |
| 25 | 0.0115 | 35.56 | | 0.0117 | 36.17 | | 0.0114 | 35.25 | 52ug |
| 50 | 0.0117 | 36.17 | | 0.0115 | 35.56 | | 0.0115 | 35.56 | |

Enzyme kinectis calculated using the Michaelis-Menten equation

**Supplementary Table 5 (continued)**

**Transient knockdown**

**OCI-Ly8 KD**

| | #1 | | | #2 | | | #3 | | |
|---|---|---|---|---|---|---|---|---|---|
| **siRNA ctrl** | A340nm/min | umol/min/mg | Vmax | A340nm/min | umol/min/mg | Vmax | A340nm/min | umol/min/mg | Vmax |
| d-iso(uM) | 0.0009 | 3.36 | 48.00 | 0.0008 | 2.99 | 49.91 | 0.0009 | 3.36 | 48.21 |
| 0.25 | 0.0017 | 6.36 | | 0.0015 | 5.61 | | 0.0016 | 5.98 | |
| 0.5 | 0.0031 | 11.59 | Km | 0.0030 | 11.22 | Km | 0.0035 | 13.09 | Km |
| 1 | 0.0065 | 24.30 | 2.24 | 0.0068 | 25.42 | 2.31 | 0.0069 | 25.80 | 2.13 |
| 2.5 | 0.0105 | 39.26 | | 0.0108 | 40.38 | | 0.0102 | 38.14 | |
| 5 | 0.0110 | 41.13 | | 0.0117 | 43.74 | | 0.0115 | 43.00 | |
| 10 | 0.0113 | 42.25 | | 0.0115 | 43.00 | | 0.0114 | 42.62 | |
| 15 | 0.0114 | 42.62 | | 0.0119 | 44.49 | | 0.0116 | 43.37 | |
| 25 | 0.0115 | 43.00 | | 0.0118 | 44.12 | | 0.0114 | 42.62 | |
| 50 | | | | | | | | | |

| | #1 | | | #2 | | | #3 | | |
|---|---|---|---|---|---|---|---|---|---|
| **D2HGDH siRNA #3** | A340nm/min | umol/min/mg | Vmax | A340nm/min | umol/min/mg | Vmax | A340nm/min | umol/min/mg | Vmax |
| d-iso(uM) | 0.0005 | 2.44 | 40.61 | 0.0008 | 3.90 | 40.78 | 0.0007 | 3.41 | 39.5 |
| 0.25 | 0.0013 | 6.33 | | 0.0012 | 5.85 | | 0.0016 | 7.79 | |
| 0.5 | 0.0029 | 14.13 | Km | 0.0028 | 13.64 | Km | 0.0026 | 12.67 | Km |
| 1 | 0.0041 | 19.97 | 2.25 | 0.0040 | 19.49 | 2.2 | 0.0045 | 21.92 | 1.84 |
| 2.5 | 0.0058 | 28.26 | | 0.0060 | 29.23 | | 0.0065 | 31.67 | |
| 5 | 0.0070 | 34.10 | | 0.0075 | 36.54 | | 0.0072 | 35.08 | |
| 10 | 0.0074 | 36.05 | | 0.0075 | 36.54 | | 0.0073 | 35.56 | |
| 15 | 0.0079 | 38.49 | | 0.0076 | 37.03 | | 0.0075 | 36.54 | |
| 25 | 0.0075 | 36.54 | | 0.0075 | 36.54 | | 0.0073 | 35.56 | |
| 50 | | | | | | | | | |

| | #1 | | | #2 | | | #3 | | |
|---|---|---|---|---|---|---|---|---|---|
| **D2HGDH siRNA #5** | A340nm/min | umol/min/mg | Vmax | A340nm/min | umol/min/mg | Vmax | A340nm/min | umol/min/mg | Vmax |
| d-iso(uM) | 0.0003 | 1.79 | 41.6 | 0.0003 | 1.79 | 42.65 | 0.0007 | 4.17 | 41.57 |
| 0.25 | 0.0011 | 6.55 | | 0.0010 | 5.95 | | 0.0013 | 7.74 | |
| 0.5 | 0.0022 | 13.10 | Km | 0.0024 | 14.29 | Km | 0.0023 | 13.70 | Km |
| 1 | 0.0039 | 23.22 | 2.1 | 0.0032 | 19.05 | 2.35 | 0.0030 | 17.86 | 2.17 |
| 2.5 | 0.0050 | 29.77 | | 0.0051 | 30.37 | | 0.0052 | 30.96 | |
| 5 | 0.0060 | 35.73 | | 0.0065 | 38.70 | | 0.0064 | 38.11 | |
| 10 | 0.0065 | 38.70 | | 0.0063 | 37.51 | | 0.0064 | 38.11 | |
| 15 | 0.0063 | 37.51 | | 0.0064 | 38.11 | | 0.0062 | 36.92 | |
| 25 | 0.0063 | 37.51 | | 0.0064 | 38.11 | | 0.0062 | 36.92 | |
| 50 | | | | | | | | | |

**Ramos KD**

| | #1 | | | 2 | | | #3 | | |
|---|---|---|---|---|---|---|---|---|---|
| **siRNA ctrl** | A340nm/min | umol/min/mg | Vmax | A340nm/min | umol/min/mg | Vmax | A340nm/min | umol/min/mg | Vmax |
| d-iso(uM) | 0.0012 | 3.86 | 25.4 | 0.0013 | 4.18 | 25.92 | 0.0013 | 4.18 | 25.49 |
| 0.25 | 0.0026 | 8.36 | | 0.0027 | 8.68 | | 0.0026 | 8.36 | |
| 0.5 | 0.0039 | 12.54 | Km | 0.0038 | 12.22 | Km | 0.0038 | 12.22 | Km |
| 1 | 0.0056 | 18.01 | 1.04 | 0.0057 | 18.33 | 1.06 | 0.0056 | 18.01 | 1.07 |
| 2.5 | 0.0067 | 21.54 | | 0.0068 | 21.86 | | 0.0065 | 20.90 | |
| 5 | 0.0070 | 22.51 | | 0.0071 | 22.83 | | 0.0070 | 22.51 | |
| 10 | 0.0076 | 24.44 | | 0.0078 | 25.08 | | 0.0078 | 25.08 | |
| 15 | 0.0076 | 24.44 | | 0.0077 | 24.76 | | 0.0075 | 24.12 | |
| 25 | 0.0075 | 24.12 | | 0.0077 | 24.76 | | 0.0076 | 24.44 | |
| 50 | | | | | | | | | |

| | #1 | | | 2 | | | #3 | | |
|---|---|---|---|---|---|---|---|---|---|
| **D2HGDH siRNA #3** | A340nm/min | umol/min/mg | Vmax | A340nm/min | umol/min/mg | Vmax | A340nm/min | umol/min/mg | Vmax |
| d-iso(uM) | 0.0012 | 4.02 | 21.76 | 0.0012 | 4.02 | 21.88 | 0.0012 | 4.02 | 21.45 |
| 0.25 | 0.0021 | 7.03 | | 0.0022 | 7.37 | | 0.0021 | 7.03 | |
| 0.5 | 0.0030 | 10.05 | Km | 0.0032 | 10.72 | Km | 0.0031 | 10.38 | Km |
| 1 | 0.0043 | 14.40 | 1.07 | 0.0049 | 16.41 | 0.97 | 0.0041 | 13.73 | 1.12 |
| 2.5 | 0.0059 | 19.76 | | 0.0055 | 18.42 | | 0.0051 | 17.08 | |
| 5 | 0.0060 | 20.10 | | 0.0060 | 20.10 | | 0.0061 | 20.43 | |
| 10 | 0.0060 | 20.10 | | 0.0063 | 21.10 | | 0.0062 | 20.77 | |
| 15 | 0.0063 | 21.10 | | 0.0062 | 20.77 | | 0.0060 | 20.10 | |
| 25 | 0.0060 | 20.10 | | 0.0062 | 20.77 | | 0.0061 | 20.43 | |
| 50 | | | | | | | | | |

| | #1 | | | 2 | | | #3 | | |
|---|---|---|---|---|---|---|---|---|---|
| **D2HGDH siRNA #5** | A340nm/min | umol/min/mg | Vmax | A340nm/min | umol/min/mg | Vmax | A340nm/min | umol/min/mg | Vmax |
| d-iso(uM) | 0.0012 | 3.94 | 19.25 | 0.0011 | 3.61 | 19.68 | 0.0011 | 3.61 | 19.68 |
| 0.25 | 0.0024 | 7.87 | | 0.0024 | 7.87 | | 0.0025 | 7.87 | |
| 0.5 | 0.0032 | 10.50 | Km | 0.0033 | 10.83 | Km | 0.0033 | 10.83 | Km |
| 1 | 0.0044 | 14.44 | 0.81 | 0.0039 | 12.80 | 0.93 | 0.0039 | 12.80 | 0.93 |
| 2.5 | 0.0051 | 16.73 | | 0.0049 | 16.08 | | 0.0049 | 16.08 | |
| 5 | 0.0055 | 18.05 | | 0.0055 | 18.05 | | 0.0056 | 18.05 | |
| 10 | 0.0055 | 18.05 | | 0.006 | 19.69 | | 0.0059 | 19.69 | |
| 15 | 0.0054 | 17.72 | | 0.0057 | 18.70 | | 0.0062 | 18.70 | |
| 25 | 0.0060 | 19.69 | | 0.0059 | 19.36 | | 0.0060 | 19.36 | |
| 50 | | | | | | | | | |

**Stable knockdown**

**HEK-293**

| | #1 | | | 2 | | | #3 | | |
|---|---|---|---|---|---|---|---|---|---|
| **siRNA ctrl** | A340nm/min | umol/min/mg | Vmax | A340nm/min | umol/min/mg | Vmax | A340nm/min | umol/min/mg | Vmax |
| d-iso(uM) | 0.0027 | 8.68 | 30.58 | 0.0028 | 9.00 | 30.85 | 0.0025 | 8.04 | 30.09 |
| 0.25 | 0.0037 | 11.90 | | 0.0039 | 12.54 | | 0.0048 | 15.43 | |
| 0.5 | 0.0061 | 19.61 | Km | 0.0068 | 21.86 | Km | 0.0070 | 22.51 | Km |
| 1 | 0.0069 | 22.19 | 0.66 | 0.0070 | 22.51 | 0.56 | 0.0071 | 22.83 | 0.49 |
| 2 | 0.0083 | 26.69 | | 0.0088 | 28.30 | | 0.0084 | 27.01 | |
| 4 | 0.0085 | 27.33 | | 0.0093 | 29.90 | | 0.0085 | 27.33 | |
| 5 | 0.0093 | 29.90 | | 0.0094 | 30.23 | | 0.0089 | 28.62 | |
| 10 | 0.0090 | 28.94 | | 0.0092 | 29.58 | | 0.0091 | 29.26 | |
| 15 | 0.0091 | 29.26 | | 0.0089 | 28.62 | | 0.0090 | 28.94 | |
| 25 | 0.0092 | 29.58 | | 0.0090 | 28.94 | | 0.0093 | 29.90 | |
| 50 | | | | | | | | | |

| | #1 | | | 2 | | | #3 | | |
|---|---|---|---|---|---|---|---|---|---|
| **D2HGDH siRNA #3** | A340nm/min | umol/min/mg | Vmax | A340nm/min | umol/min/mg | Vmax | A340nm/min | umol/min/mg | Vmax |
| d-iso | 0.0020 | 6.43 | 25.26 | 0.0022 | 7.07 | 24.63 | 0.0020 | 6.43 | 25.43 |
| 0.25 | 0.0032 | 10.29 | | 0.0035 | 11.25 | | 0.0044 | 14.15 | |
| 0.5 | 0.0050 | 16.08 | Km | 0.0053 | 17.04 | Km | 0.0053 | 17.04 | Km |
| 1 | 0.0059 | 18.97 | 0.67 | 0.0056 | 18.01 | 0.59 | 0.0059 | 18.97 | 0.51 |
| 2 | 0.0066 | 21.22 | | 0.0063 | 20.26 | | 0.0072 | 23.15 | |
| 4 | 0.0068 | 21.86 | | 0.0065 | 20.90 | | 0.0073 | 23.47 | |
| 5 | 0.0076 | 24.44 | | 0.0074 | 23.79 | | 0.0078 | 25.08 | |
| 10 | 0.0075 | 24.12 | | 0.0075 | 24.12 | | 0.0073 | 23.47 | |
| 15 | 0.0076 | 24.44 | | 0.0075 | 24.12 | | 0.0078 | 25.08 | |
| 25 | 0.0077 | 24.76 | | 0.0078 | 25.08 | | 0.0077 | 24.76 | |
| 50 | | | | | | | | | |

| | #1 | | | 2 | | | #3 | | |
|---|---|---|---|---|---|---|---|---|---|
| **D2HGDH siRNA #5** | A340nm/min | umol/min/mg | Vmax | A340nm/min | umol/min/mg | Vmax | A340nm/min | umol/min/mg | Vmax |
| d-iso | 0.0023 | 7.40 | 26 | 0.0022 | 7.07 | 25.91 | 0.0024 | 7.72 | 25.85 |
| 0.25 | 0.0036 | 11.58 | | 0.0036 | 11.58 | | 0.0041 | 13.18 | |
| 0.5 | 0.0055 | 17.68 | Km | 0.0061 | 19.61 | Km | 0.0058 | 18.65 | Km |
| 1 | 0.0067 | 21.54 | 0.53 | 0.0057 | 18.33 | 0.56 | 0.0062 | 19.94 | 0.49 |
| 2 | 0.0074 | 23.79 | | 0.0069 | 22.19 | | 0.0075 | 24.12 | |
| 4 | 0.0075 | 24.12 | | 0.0073 | 23.47 | | 0.0070 | 22.51 | |
| 5 | 0.0076 | 24.44 | | 0.0078 | 25.08 | | 0.0078 | 25.08 | |
| 10 | 0.0077 | 24.76 | | 0.0078 | 25.08 | | 0.0076 | 24.44 | |
| 15 | 0.0078 | 25.08 | | 0.0079 | 25.40 | | 0.0079 | 25.40 | |
| 25 | 0.0078 | 25.08 | | 0.0079 | 25.40 | | 0.0080 | 25.72 | |
| 50 | | | | | | | | | |

Enzyme kinectis calculated using the Michaelis-Menten equation

**Supplementary Table 6. IDH activiity assay in subcellular fractions**

**Cytosolic fraction**

| **MSCV** | A340nm/min | | | umol NADPH/min/mg | | | mean | SD | | |
|---|---|---|---|---|---|---|---|---|---|---|
| D-isocitrate(uM) | #1 | #2 | #3 | #1 | #2 | #3 | umolNADPH/min/mg | | | |
| 0.25 | 0.0014 | 0.0013 | 0.0013 | 4.33 | 4.02 | 4.02 | 4.12 | 0.18 | | |
| 0.5 | 0.0020 | 0.0021 | 0.0019 | 6.18 | 6.49 | 5.87 | 6.18 | 0.31 | Best fit values | Std. Error |
| 1 | 0.0035 | 0.0034 | 0.0033 | 10.82 | 10.51 | 10.20 | 10.51 | 0.31 | Vmax | |
| 2.5 | 0.0040 | 0.0039 | 0.0039 | 12.37 | 12.06 | 12.06 | 12.16 | 0.18 | 17.65 | 0.26 |
| 5 | 0.0049 | 0.0048 | 0.0050 | 15.15 | 14.84 | 15.46 | 15.15 | 0.31 | Km | |
| 10 | 0.0054 | 0.0057 | 0.0055 | 16.70 | 17.62 | 17.00 | 17.11 | 0.47 | 0 | 0.0585 |
| 25 | 0.0053 | 0.0054 | 0.0058 | 16.39 | 16.70 | 17.93 | 17.00 | 0.82 | | |
| 50 | 0.0053 | 0.0055 | 0.0057 | 16.44 | 17.06 | 17.68 | 17.06 | 0.62 | | |

| **D2HGDH WT** | A340nm/min | | | umol NADPH/min/mg | | | mean | SD | | |
|---|---|---|---|---|---|---|---|---|---|---|
| D-isocitrate(uM) | #1 | #2 | #3 | #1 | #2 | #3 | umolNADPH/min/mg | | | |
| 0.25 | 0.0013 | 0.0014 | 0.0013 | 3.88 | 4.18 | 3.88 | 3.98 | 0.17 | | |
| 0.5 | 0.0019 | 0.0020 | 0.0020 | 5.68 | 5.98 | 5.98 | 5.88 | 0.17 | Best fit values | Std. Error |
| 1 | 0.0034 | 0.0035 | 0.0032 | 10.16 | 10.46 | 9.56 | 10.06 | 0.46 | Vmax | |
| 2.5 | 0.0039 | 0.0040 | 0.0040 | 11.65 | 11.95 | 11.95 | 11.85 | 0.17 | 17.77 | 0.27 |
| 5 | 0.0050 | 0.0050 | 0.0052 | 14.94 | 14.94 | 15.54 | 15.14 | 0.35 | Km | |
| 10 | 0.0058 | 0.0057 | 0.0059 | 17.33 | 17.03 | 17.63 | 17.33 | 0.30 | 0.9171 | 0.0641 |
| 25 | 0.0057 | 0.0058 | 0.0056 | 17.03 | 17.33 | 16.73 | 17.03 | 0.30 | | |
| 50 | 0.0057 | 0.0058 | 0.0055 | 17.09 | 17.39 | 16.49 | 16.99 | 0.46 | | |

**Mitochondrial fraction**

| **MSCV** | A340nm/min | | | umol NADPH/min/mg | | | mean | SD | | |
|---|---|---|---|---|---|---|---|---|---|---|
| D-isocitrate(uM) | #1 | #2 | #3 | #1 | #2 | #3 | umolNADPH/min/mg | | | |
| 0.25 | 0.0004 | 0.0003 | 0.0004 | 11.69 | 8.77 | 11.69 | 10.72 | 1.69 | | |
| 0.5 | 0.0006 | 0.0007 | 0.0006 | 17.54 | 20.46 | 17.54 | 18.51 | 1.69 | Best fit values | Std. Error |
| 1 | 0.0011 | 0.0010 | 0.0012 | 32.15 | 29.23 | 35.08 | 32.15 | 2.92 | Vmax | |
| 2.5 | 0.0013 | 0.0014 | 0.0013 | 38.00 | 40.92 | 38.00 | 38.97 | 1.69 | 58.8 | 1.18 |
| 5 | 0.0017 | 0.0016 | 0.0016 | 49.69 | 46.77 | 46.77 | 47.74 | 1.69 | Km | |
| 10 | 0.0019 | 0.0020 | 0.0020 | 55.54 | 58.46 | 58.46 | 57.49 | 1.69 | 1.018 | 0.0932 |
| 25 | 0.0019 | 0.0019 | 0.0020 | 55.54 | 55.54 | 58.46 | 56.51 | 1.69 | | |
| 50 | 0.0021 | 0.0018 | 0.0018 | 61.58 | 52.79 | 52.79 | 55.72 | 5.08 | | |

| **D2HGDH WT** | A340nm/min | | | umol NADPH/min/mg | | | mean | SD | | |
|---|---|---|---|---|---|---|---|---|---|---|
| D-isocitrate(uM) | #1 | #2 | #3 | #1 | #2 | #3 | umolNADPH/min/mg | | | |
| 0.25 | 0.0005 | 0.0006 | 0.0005 | 14.62 | 17.54 | 14.62 | 15.59 | 1.69 | | |
| 0.5 | 0.0008 | 0.0009 | 0.0008 | 23.38 | 26.31 | 23.38 | 24.36 | 1.69 | Best fit values | Std. Error |
| 1 | 0.0016 | 0.0015 | 0.0015 | 46.77 | 43.85 | 43.85 | 44.82 | 1.69 | Vmax | |
| 2.5 | 0.0019 | 0.0017 | 0.0019 | 55.54 | 49.69 | 55.54 | 53.59 | 3.38 | 84.3 | 1.66 |
| 5 | 0.0025 | 0.0024 | 0.0025 | 73.08 | 70.15 | 73.08 | 72.10 | 1.69 | Km | |
| 10 | 0.0028 | 0.0029 | 0.0028 | 81.85 | 84.77 | 81.85 | 82.82 | 1.69 | 1.063 | 0.0948 |
| 25 | 0.0027 | 0.0028 | 0.0027 | 78.92 | 81.85 | 78.92 | 79.90 | 1.69 | | |
| 50 | 0.0027 | 0.0027 | 0.0026 | 79.18 | 79.18 | 76.25 | 78.20 | 1.69 | | |

Enzyme kinectis calculated using the Michaelis-Menten equation

**Supplementary Table 6 (continuation)**

**Cytosolic fraction**

| **siRNA ctrl** | A340nm/min | | | umol NADPH/min/mg | | | mean | SD | | |
|---|---|---|---|---|---|---|---|---|---|---|
| D-isocitrate(uM) | #1 | #2 | #3 | #1 | #2 | #3 | umolNADPH/min/mg | | | |
| 0.25 | 0.0018 | 0.0017 | 0.0016 | 5.10 | 4.82 | 4.54 | 4.82 | 0.28 | | |
| 0.5 | 0.0025 | 0.0024 | 0.0025 | 7.09 | 6.81 | 7.09 | 6.99 | 0.16 | Best fit va Std. Error | |
| 1 | 0.0042 | 0.0041 | 0.0041 | 11.91 | 11.63 | 11.63 | 11.72 | 0.16 | Vmax | |
| 2.5 | 0.0048 | 0.0047 | 0.0046 | 13.61 | 13.33 | 13.04 | 13.33 | 0.28 | 17.99 | 0.24 |
| 5 | 0.0058 | 0.0057 | 0.0056 | 16.45 | 16.16 | 15.88 | 16.16 | 0.28 | | |
| 10 | 0.0063 | 0.0062 | 0.0061 | 17.86 | 17.58 | 17.30 | 17.58 | 0.28 | Km | |
| 25 | 0.0061 | 0.0062 | 0.0060 | 17.30 | 17.58 | 17.01 | 17.30 | 0.28 | 0.6772 | 0.044 |
| 50 | 0.0059 | 0.0064 | 0.0060 | 16.78 | 18.21 | 17.07 | 17.35 | 0.75 | | |

| **D2HGDH siRNA #3** | A340nm/min | | | umol NADPH/min/mg | | | mean | SD | | |
|---|---|---|---|---|---|---|---|---|---|---|
| D-isocitrate(uM) | #1 | #2 | #3 | #1 | #2 | #3 | umolNADPH/min/mg | | | |
| 0.25 | 0.0015 | 0.0016 | 0.0014 | 4.54 | 4.84 | 4.24 | 4.54 | 0.30 | Best fit va Std. Error | |
| 0.5 | 0.0021 | 0.0021 | 0.0023 | 6.36 | 6.36 | 6.96 | 6.56 | 0.35 | Vmax | |
| 1 | 0.0037 | 0.0038 | 0.0036 | 11.20 | 11.51 | 10.90 | 11.20 | 0.30 | 17.66 | 0.24 |
| 2.5 | 0.0043 | 0.0044 | 0.0042 | 13.02 | 12.72 | 12.72 | 12.82 | 0.17 | Km | |
| 5 | 0.0052 | 0.0053 | 0.0051 | 15.74 | 16.05 | 15.44 | 15.74 | 0.30 | 0.7263 | 0.048 |
| 10 | 0.0056 | 0.0057 | 0.0055 | 16.96 | 17.26 | 16.65 | 16.96 | 0.30 | | |
| 25 | 0.0057 | 0.0059 | 0.0055 | 17.26 | 17.86 | 16.65 | 17.26 | 0.61 | | |
| 50 | 0.0055 | 0.0058 | 0.0054 | 16.71 | 17.62 | 16.40 | 16.91 | 0.63 | | |

| **D2HGDH siRNA #5** | A340nm/min | | | umol NADPH/min/mg | | | mean | SD | | |
|---|---|---|---|---|---|---|---|---|---|---|
| D-isocitrate(uM) | #1 | #2 | #3 | #1 | #2 | #3 | umolNADPH/min/mg | | | |
| 0.25 | 0.0013 | 0.0012 | 0.0012 | 4.25 | 3.92 | 3.92 | 4.03 | 0.19 | Best fit va Std. Error | |
| 0.5 | 0.0018 | 0.0019 | 0.0018 | 5.88 | 6.21 | 5.88 | 5.99 | 0.19 | Vmax | |
| 1 | 0.0032 | 0.0033 | 0.0031 | 10.46 | 10.78 | 10.13 | 10.46 | 0.33 | 17.57 | 0.21 |
| 2.5 | 0.0039 | 0.0041 | 0.0038 | 12.74 | 13.40 | 12.42 | 12.85 | 0.50 | Km | |
| 5 | 0.0048 | 0.0047 | 0.0048 | 15.69 | 15.36 | 15.69 | 15.58 | 0.19 | 0.8098 | 0.046 |
| 10 | 0.0052 | 0.0051 | 0.0052 | 16.99 | 16.67 | 16.99 | 16.88 | 0.19 | | |
| 25 | 0.0052 | 0.0051 | 0.005 | 16.99 | 16.34 | 16.34 | 16.56 | 0.38 | | |
| 50 | 0.0051 | 0.0052 | 0.0052 | 16.72 | 17.05 | 17.05 | 16.94 | 0.19 | | |

**Mitochondrial fraction**

| **siRNA ctrl** | A340nm/min | | | umol NADPH/min/mg | | | mean | SD | | |
|---|---|---|---|---|---|---|---|---|---|---|
| D-isocitrate(uM) | #1 | #2 | #3 | #1 | #2 | #3 | umolNADPH/min/mg | | | |
| 0.25 | 0.0007 | 0.0006 | 0.0007 | 17.29 | 14.82 | 17.29 | 16.46 | 1.43 | | |
| 0.5 | 0.0009 | 0.0010 | 0.0010 | 22.23 | 24.70 | 24.70 | 23.87 | 1.43 | Best fit va Std. Error | |
| 1 | 0.0019 | 0.0019 | 0.0017 | 46.92 | 46.92 | 41.98 | 45.28 | 2.85 | Vmax | |
| 2.5 | 0.0024 | 0.0025 | 0.0023 | 59.27 | 61.74 | 56.80 | 59.27 | 2.47 | 84.92 | 1.42 |
| 5 | 0.0030 | 0.0029 | 0.0028 | 74.09 | 71.62 | 69.15 | 71.62 | 2.47 | | |
| 10 | 0.0033 | 0.0035 | 0.0032 | 81.50 | 86.44 | 79.03 | 82.32 | 3.77 | Km | |
| 25 | 0.0034 | 0.0032 | 0.0032 | 83.97 | 79.03 | 79.03 | 80.67 | 2.85 | 1.012 | 0.07732 |
| 50 | 0.0031 | 0.0033 | 0.0032 | 76.80 | 81.76 | 79.28 | 79.28 | 2.48 | | |

| **D2HGDH siRNA #3** | A340nm/min | | | umol NADPH/min/mg | | | mean | SD | | |
|---|---|---|---|---|---|---|---|---|---|---|
| D-isocitrate(uM) | #1 | #2 | #3 | #1 | #2 | #3 | umolNADPH/min/mg | | | |
| 0.25 | 0.0003 | 0.0004 | 0.0002 | 9.74 | 12.99 | 6.50 | 9.74 | 3.25 | Best fit va Std. Error | |
| 0.5 | 0.0005 | 0.0005 | 0.0004 | 16.24 | 16.24 | 12.99 | 15.16 | 1.88 | Vmax | |
| 1 | 0.0009 | 0.0009 | 0.0008 | 29.23 | 29.23 | 25.98 | 28.15 | 1.88 | 55.35 | 1.20 |
| 2.5 | 0.0012 | 0.0011 | 0.0011 | 38.97 | 35.73 | 35.73 | 36.81 | 1.88 | Km | |
| 5 | 0.0014 | 0.0015 | 0.0014 | 45.47 | 48.72 | 45.47 | 46.55 | 1.88 | 1.105 | 0.108 |
| 10 | 0.0016 | 0.0018 | 0.0015 | 51.97 | 58.46 | 48.72 | 53.05 | 4.96 | | |
| 25 | 0.0017 | 0.0016 | 0.0015 | 55.21 | 51.97 | 48.72 | 51.97 | 3.25 | | |
| 50 | 0.0016 | 0.0017 | 0.0015 | 52.13 | 55.39 | 48.88 | 52.13 | 3.26 | | |

| **D2HGDH siRNA #5** | A340nm/min | | | umol NADPH/min/mg | | | mean | SD | | |
|---|---|---|---|---|---|---|---|---|---|---|
| D-isocitrate(uM) | #1 | #2 | #3 | #1 | #2 | #3 | umolNADPH/min/mg | | | |
| 0.25 | 0.0005 | 0.0004 | 0.0005 | 14.59 | 11.67 | 14.59 | 13.62 | 1.68 | Best fit va Std. Error | |
| 0.5 | 0.0007 | 0.0006 | 0.0006 | 20.42 | 17.51 | 17.51 | 18.48 | 1.68 | Vmax | |
| 1 | 0.0011 | 0.0010 | 0.0011 | 32.10 | 29.18 | 32.10 | 31.12 | 1.68 | 54.4 | 0.89 |
| 2.5 | 0.0013 | 0.0012 | 0.0013 | 37.93 | 35.01 | 37.93 | 36.96 | 1.68 | Km | |
| 5 | 0.0016 | 0.0015 | 0.0016 | 46.69 | 43.77 | 46.69 | 45.71 | 1.68 | 0.8854 | 0.068 |
| 10 | 0.0018 | 0.0017 | 0.0018 | 52.52 | 49.60 | 52.52 | 51.55 | 1.68 | | |
| 25 | 0.0019 | 0.0018 | 0.0017 | 55.44 | 49.60 | 49.60 | 51.55 | 3.37 | | |
| 50 | 0.0019 | 0.0018 | 0.0019 | 55.62 | 52.69 | 55.62 | 54.64 | 1.69 | | |

Enzyme kinectis calculated using the Michaelis-Menten equation

## Supplementary Table 7 - Mutational status of chromatin modifiers in DLBCL cell lines

| Cell line | D2HGDH | MLL2 | MLL3 | MLL4 | MLL5 | CREBBP | EP300 | EZH2 |
|---|---|---|---|---|---|---|---|---|
| WSU-NHL | Mutant | WT[1-3] | WT[1-3] | WT[1-3] | WT[1-3] | Mutant[1-3] | WT[1-3] | WT[1-3] |
| OCI-Ly7 | Mutant | WT[1-3] | Mutant[2] | WT[2] | WT[2] | WT[1-3,5] | WT[1-3,5] | WT[1-3,5] |
| SU-DHL6 | Mutant | Mutant[1,3,5] | WT[1,3,5] | WT[1,3,5] | WT[1,3,5] | WT[5]; Mutant[1,3] | Mutant[1,3,5,7] | Mutant[1,3,5,6] |
| SU-DHL8 | Mutant | Mutant[1,3] | WT[1,3] | WT[1,3] | WT[1,3] | Mutant[1,3] | Mutant[1,3,7] | WT[1,3,7] |
| SU-DHL4 | WT | WT[1-3] | WT[1-3] | WT[1-3] | WT[1-3] | WT[1-3] | WT[1,3] Del[7] | WT[2,7], Mutant[1,3] |
| SU-DHL10 | WT | Mutant [1,3] | WT[1,3] | WT[1,3] | WT[1,3] | Mutant[1,3] | Mutant[1,3,7] | WT[1,3,7] |
| OCI-Ly1 | WT | Mutant[1,3,5] | WT[1-3,5,6] | WT[1-3,5,6] | WT[1-3,5,6] | Mutant[1,3] WT[2,5] | WT[1-6] | Mutant[1-3,5,6] |
| OCI-Ly4 | WT | Mutant[1,3] | WT[1,3] | WT[1,3] | WT[1,3] | WT[1,3] | WT[1,3] | WT[1,3] |
| OCI-Ly18 | WT | Mutant[1,3] | WT[1] | WT[1,3] | WT[1,3] | WT[1,3] | WT[1,3] | WT[1,3] |
| SU-DHL2 | WT | WT[1,3] | WT[1,3] | WT[1,3] | WT[1,3] | WT[1,3] | Del[1,3] | WT[1,3] |
| Farage | WT | WT[2] | WT[2] | WT[2] | WT[2] | WT[2] | WT[2] | WT[2] |
| OCI-Ly8 | WT | WT[1-3] | WT[1-3] | WT[1-3] | WT[1-3] | Mutant[1,3] WT[2] | WT[1,3,4] | WT[1-3] |
| OCI-Ly10 | WT | WT[1-3] | WT[1-4] | WT[1-4] | WT[1-4] | WT[1-4] | WT[1,3,4] M[7] | WT[1-4,7] |
| OCI-Ly19 | WT | WT[2,5] | WT[2,5] | WT[2,5] | WT[2,5] | WT[2,5] | WT[2,5] | WT[2,5,7] |

The cell lines analyzed for the histone and DNA methylation, HIF hydroxylation and alpha-KG or D2-HG levels (Figure 7 and Supplementary Figure 12) are listed above.
The remaining cell lines sequenced for D2HGDH (Supplementary Table1) are listed below.

MLL2 and MLL3 encode histone methyltransferase that methylates H3K4; MLL2 and MLL3 mutations are likely loss-of-function thus leading to lower methylation of H3K4, yet cell lines mutant for both D2HGDH and MLL2 or MLL3 display high H3K4me3 levels.

The methylation of H3K4m3 was higher in OCI-Ly7, SU-DHL6 and SU-DHL8, than in SU-DHL10, OCI-Ly1, OCI-Ly4 or OCI- Ly18 and thus appear to be driven by D2HGDH

| Cell line | D2HGDH | MLL2 | MLL3 | MLL4 | MLL5 | CREBBP | EP300 | EZH2 |
|---|---|---|---|---|---|---|---|---|
| HBL-1 | WT | WT[1,3,4] | WT[1,3] M[4] | WT[1,3,4] | M[4] | WT[1,3] M[4] | WT[1,3,4] | WT[1,3,4] |
| HT | WT | WT[2] | WT[2] | WT[2] | WT[2] | WT[2] | WT[2] | WT[2,7] |
| K1106P | WT | NA | NA | NA | NA | NA | NA | NA |
| K422 | WT | WT[2,6] M[5] | WT[2,5,6] | WT[2,5,6] | WT[2,5,6] | WT[2] M[5] | M[5] | M[2,5,6] |
| OCI-Ly3 | WT | WT[1-4] | WT[1-4] | M[4] | WT[1-4] | WT[1-4] | WT[1-4] | WT[1-4] |
| NU-DHL1 | WT | M[5] | WT [5] | WT [5] | WT [5] | M[5] | WT [5] | WT[5,7] |
| NU-DUL1 | WT | M[5] | WT [5] | WT [5] | WT [5] | WT [5] | WT [5] | WT[5,7] |
| Pfeiffer | WT | WT[2] M[5] | WT[2] | WT[2] | WT[2] | M[2] | M[7] | WT[2,7] |
| RC-K8 | WT | WT[1-3] | WT[1-3] | WT[1-3] | WT[1-3] | WT[1-3] | Del[1,3] | WT[1-3] |
| SU-DHL5 | WT | WT[1,3] | WT[1,3] | WT[1,3] | WT[1,3] | Del[1,3] | WT[1,3] Del[7] | Del[1,3,7] |
| SU-DHL7 | WT | WT[1-3] | WT[1-3] | WT[1-3] | WT[1-3] | WT[2] M[1,3] | WT[1-3] | WT[1-3] |
| SU-DHL16 | WT | NA | NA | NA | NA | NA | NA | NA |
| Toledo | WT | M[5] | WT[2] M[7] | WT[2,5] | WT[2,5] | WT[2,5] | M[7] | WT[2,5,7] |
| U2932 | WT | WT[1-3] | WT[1-3] | WT[1-3] | WT[1-3] | WT[1-3] | WT[1-3] | WT[1-3] |
| USC-DHL1 | WT | NA | NA | NA | NA | NA | NA | NA |

**Supplementary Table 8 - Mutational status of chromatin modifiers in DLBCL**

| Tumor ID | D2HGDH | MLL2 (% variant allele) | MLL3 (% variant allele) | MLL4 | MLL5 | EZH2 (% variant allele) | EP300 (% variant allele) | CREBBP (% variant allele) | TET2 |
|---|---|---|---|---|---|---|---|---|---|
| **2202** | WT | c.C11968T:p.Q3990X (25%); c.C6520T:p.Q2174X (37%) | WT | WT | WT | WT | c.C5597T:p.P1866L (34%) | WT | WT |
| **3416** | WT | WT | c.C5636T:p.P1879L (20%) | WT | WT | WT | WT | WT | WT |
| **4236** | WT | WT | WT | WT | WT | WT | WT | WT | WT |
| **4522** | WT | WT | WT | WT | WT | WT | WT | WT | WT |
| **5234** | A426T | WT | WT | WT | WT | WT | WT | WT | WT |
| **5485** | WT | c.A15176T:p.H5059L (82%) | WT | WT | WT | c.A1769T:p.Y641F (38%) | c.T4197G:p.D1399E (39%) | c.C4378T:p.R1460X (78%) | WT |
| **6902** | A426T | WT | WT | WT | WT | WT | WT | WT | WT |
| **7518** | WT | c.10982delG:p.G3661fs (67%) | WT | WT | WT | c.C1907T:p.A687V (17%) | WT | WT | WT |
| **7572** | WT | WT | WT | WT | WT | WT | c.4057delT:p.F1353fs (40%) | WT | WT |

**Supplementary Table 9 - D2HGDH mutational status in cancer**

| Tumor Type | Mutation - aa modification | Frequency* | Somatic Status | References |
|---|---|---|---|---|
| Glioma (GBM) | M153T, S178G, S197T, N443S | <1% | confirmed somatic | TCGA (COSU329, COSU545) and Nature Genetics 2013;45(10):1141-9 |
| Endometrioid carcinoma | R107Q, V142I, A170V, R215Q, A420T | 1.2% | somatic or unknown | TCGA (COSU419) |
| Colon Rectal Cancer | G98S, S197G#, D403N, L505M | 4.7% (3/70 cases) #cell line | confirmed somatic | Nature 2012;488:660-4 |
| Lung adenocarcinoma/squamous cel | Q129H, S149F, G201W, K252N, T390K, G438C, A455T | <1% | somatic or unknown | TCGA (COSU417/418); Cell 2012;150(6):1107-20; Nature Genetics 2012, 44(10):1104-10 and :1111-6 |
| Melanoma | P141L, R215*, F336L, P424L, E447K, E475K | 1.7% | confirmed somatic | TCGA (COSU540) |
| Stomach - adenocarcinoma | G193R, A373T, D413L | <1% | confirmed somatic | TCGA (COSU413/) |
| Bladder | A446T, G477A, | <1% | confirmed somatic | TCGA (COSU541/581) |
| Burkitt Lymphoma | C172Y | <1% | unknown | Nature 2012;490:116-20 |

Green font - same residue was reoprted mutated in D-2-hydroxyglutaric aciduria (D2-HGA
Red font - same mutation found in D2-HGA patients
When it was investigated, all mutations were found to be heterozygou

*To be more stringent, all SNPs - rare or frequent - were removed from this analysis, even when the variant was described in COSMIC as a somatic ever

**Supplementary Table 10 – Primers sequence**

**D2HGDH – PCR and sequencing**
D-HGex2F 5'-TGCTTCTGCAAGCGTGTTTC-3'
D-HGex2R 5'-TTTGAAGCCTCCACGGGAAG-3'
D-HGex3F 5'-GAGTGACCACTTGCCTCATC-3'
D-HGex3R 5'-AACCAAGATGTCATCGGCTG-3'
D-HGex4F 5'-GCAGGGTAATCAGGATTTGG-3'
D-HGex4R 5'-GCCCTAACTCATTCACCCAC-3'
D-HGex5F 5'-GTTCCTTCTGGGTGGCTTG-3'
D-HGex5R 5'-ATGAGAGCCGTGAGAGGAC-3'
D-HGex6F 5'-GTCCATCCTTCAGCCTCTTG-3'
D-HGex6R 5'-CTTCCTCACACCAACAGTG-3'
D-HGex7F 5'-TGTTTGTTGCAGTGCCAGTC-3'
D-HGex7R 5'-TGTGTCCAGACGTGCAGAAG-3'
D-HGex8F 5'-TCTTGGCCACGAAAGATCAG-3'
D-HGex8R 5'-CTGTCTAGGCTGCACCAATG-3'
D-HGex9F 5'-ATACAGAACATGCTGCTGCC-3'
D-HGex9R 5'-GATATGCTAAGCCAGAGACC-3'
D-HGex10F 5'-ATCTTGGGAGGGGCTGTTG-3'
D-HGex10R 5'-TTGGCAGCAGCAGGAGTG-3'

**L2HGDH – PCR and sequencing**
L-HGex1F 5'-AAGGCGCGCCACTTCATTG-3'
L-HGex1R 5'-CGGGACAGGGAAATACGAAC-3'
L-HGex2F 5'-TGCATGTGAAGTTTGGCGAG-3'
L-HGex2R 5'-CACTGACATTCAGCATGAAAG-3'
L-HGex4F 5'-CTCCTTTGGGTCATACAATAG-3'
L-HGex4R 5'-CTGTGACAGGATTATCTAACTG-3'
L-HGex5F 5'-TAGCAGCAAGAAAAGCTTGG-3'
L-HGex5R 5'-ATGGAGGGCTGACTATATTC-3'
L-HGex6F 5'-GGTGCAATCATAGTAATGAC-3'
L-HGex6R 5'-ACTTAAAATACAGCCCTGTG-3'
L-HGex7F 5'-CCCTCTTGACCTATTCTAC-3'
L-HGex7R 5'-CATCTCCTTTATGACCACC-3'
L-HGex8F 5'-TGCATGAGAAGAAAGTGTTTTATG-3'
L-HGex8R 5'-CCAATCACAAATATGGGGATTTAC-3'
L-HGex9F 5'-GCCTAGATTTTTGTGATGAC -3'
L-HGex9R 5'-GTATTTACACTCCTTATCCC -3'
L-HGex10F 5'-CGCTGACTTGTAAAGTATCC-3'
L-HGex10R 5'-TGCAGTGGTTATCTTTGACC-3'

**IDH1/IDH2 – PCR and sequencing**
IDH1F 5'-GAGCTCTATATGCCATCACTGC-3'
IDH1R 5'-CAAGTTGGAAATTTCTGGGC-3'
IDH2F 5'-ATTCTGGTTGAAAGATGGCG-3'
IDH2R 5'-ACAAGAGGATGGCTAGGCG-3'

**D2HGDH – cloning, mutagenesis, RT-PCR**
D2F-BglII gatagatctAAGGAGGAGCCCGAGGTC
D2R-XhoI cactcgagAGCAGCAGGAGTGGCCGTCAG
D2-131F 5'GAACCTGGCCGTGAACCCACAGGGGGxCAACACAGGCATGGTG 3'
D2-131R 5'CACCATGCCTGTGTTGxCCCCCTGTGGGTTCACGGCCAGGTTC 3'
D2-208F 5'GGGGGAAACGTGGCAACCAACACTGGAGGCCTGCGGTTTCTTC 3'
D2-208R 5'GAAGAAACCGCAGGCCTCCAGTGTTGGTTGCCACGTTTCCCCC 3'
D2-212F 5' CAACCAACGCTGGAGGCCTGTGGTTTCTTCGATATGGCTCA 3'
D2-212R 5' TGAGCCATATCGAAGAAACCACAGGCCTCCAGCGTTGGTTG 3'
D2-421F 5'CGTGACTGACCTGCGCGCCCACCTCGGCCCGCACGCCAAGC 3'
D2-421R 5'GCTTGGCGTGCGGGCCGAGGTGGGCGCGCAGGTCAGTCACG 3'
D2-426F 5' GCCCGCCTCGGCCCGCACACCAAGCACGTGGTGGGCTATGG 3'
D2-426R 5' CCATAGCCCACCACGTGCTTGGTGTGCGGGCCGAGGCGGGC 3'
D2F-Rtime 5' GATATGGCTCACTGCATGGGAC 3' (exon 5)
D2R-Rtime 5' GTCTGCAGAACCTCAGCAAAGC 3' (exon 7)

**D2HGDH – siRNA**
D2HGDH shRNA #3 target sequence: CTGTCATGAATGTCCAGTA
5'- GATCCTGTCATGAATGTCCAGTAATTCAAGAGATTACTGGACATTCATGACAGAA - 3'
5' - AGCTTTCTGTCATGAATGTCCAGTAATCTCTTGAATTACTGGACATTCATGACAG - 3'
D2HGDH shRNA #5 target sequence: CCCAGCTGGGAAGACAGTT
5' - GATCCCCAGCTGGGAAGACAGTTATTCAAGAGATAACTGTCTTCCCAGCTGGCAA - 3'
5' - AGCTTTGCCAGCTGGGAAGACAGTTATCTCTTGAATAACTGTCTTCCCAGCTGGG - 3'

**D2HGDH – tagged constructs**
D2HGDH cloning into pHM6 – n-terminus HA tag:
D2HAFw-HindIII – ataagcttgCTGCCCCGTCGGCCTCT
D2HARv-NotI - aagcggccgcgaGGCCTGGCTGGGCA

D2HGDH cloning into p3xFLAG-CMV – c-terminus FLAG tag:
D2FlagFw-HindIII - ataagcttGAGCCCGAGGTCTCCGTC
D2FlagRv-BamHI - ttggatccGGCCTGGCTGGGCAGCGT

**IDH2 - cloning and RT-PCR oligos**
IDH2F-XhoI - tactcgagTCTCCAGCTTGGGATG
IDH2R-EcoRI - gagaattcCTCCACTGCAGCCATG
IDH2-QPCR-Fw 5' AGCTGGATGGGAACCAAGAC 3' (exon 9)
IDH2-QPCR-Rv 5' CTCGTTCAGCTTCACATTGC 3' (exon 11)

**Supplementary Table 11. Antibodies validation**

| Antibody | Commercial source | Validation |
|---|---|---|
| D2HGDH | Proteintech, #13895-1-AP | Antibodypedia |
| H3K4me3 | Cell Signaling Technologies #9751 | *Nature* 483, 474-78 (2012). |
| H3K9me2 | Cell Signaling Technologies #9753 | *Nature*, 464:306-10 (2010) |
| H3K27me3 | Cell Signaling Technologies #9733 | *Nature*, 464:306-10 (2010) |
| H3K36me3 | Cell Signaling Technologies #4909 | *Nature*, 469:231-05 (2011) |
| H3K79me2 | Cell Signaling Technologies #9757 | *Nature* 483, 474-78 (2012) |
| Total histone H3 | Cell Signaling Technologies #4499 | J Immunol 186:3986-96 (2011). |
| HIF1α | BD Biosciences #61095 | 1degreebio.org |
| HIF1α-hydroxyproline (Pro402) | Millipore #07-1585 | J. Biol. Chem., 286: 13041-51 (2011) |
| GLUT1 | Novus Biologicals # NB300-666 | Antibodypedia |
| IDH1 | Cell Signaling Technology #8137 | Science 324:261-5 (2009) |
| IDH2 | Abcam, #ab55271 | *Nature* 483, 474-78 (2012) |
| β-actin | Sigma-Aldrich, #A2228 | 1degreebio.org |

**Supplementary Table 12 - Metabolites quantification**

**D2HGDH stable expression cell model**

D2-HG pmol/mg of protein - assay#1, 5 replicates

| | | | | | |
|---|---|---|---|---|---|
| MSCV | 8.431277 | 9.934035 | 15.45281 | 13.72072 | 12.26284 |
| D2HGDH - WT | 1.502575 | 1.806834 | 2.834707 | 2.913745 | 2.851159 |
| D2HGDH - G131X | 8.921748 | 9.508083 | 7.696046 | 6.301116 | 6.145552 |
| D2HGDH - A208T | 4.66838 | 5.456726 | 6.028038 | 6.740521 | 4.368945 |
| D2HGDH - R212W | 5.536191 | 7.573859 | 6.988648 | 8.26961 | 7.989582 |
| D2HGDH - R421H | 1.335891 | 2.469225 | 2.337813 | 1.663264 | 1.389835 |
| D2HGDH - A426T | 2.344331 | 3.82037 | 2.875458 | 5.230389 | 5.053128 |

D2-HG pmol/mg of protein - assay#2, 3 replicates

| | | | |
|---|---|---|---|
| MSCV | 26.34733 | 19.78871 | 13.83083 |
| D2HGDH - WT | 9.383159 | 13.36428 | 9.20061 |
| D2HGDH - G131X | 17.01825 | 18.52593 | 22.26802 |
| D2HGDH - A208T | 23.99778 | 26.22417 | 20.56295 |
| D2HGDH - R421H | 5.51374 | 8.497204 | 7.246866 |
| D2HGDH - A426T | 7.529353 | 10.59393 | 7.630245 |

L2-HG pmol/mg of protein, 3 replicates

| | | | |
|---|---|---|---|
| MSCV | 179.3523 | 113.1284 | 123.4135 |
| D2HGDH - WT | 141.5041 | 148.9788 | 125.1581 |
| D2HGDH - G131X | 125.0785 | 116.308 | 109.4466 |
| D2HGDH - A208T | 167.5777 | 138.9724 | 155.4484 |
| D2HGDH - R421H | 111.0916 | 91.20035 | 92.75989 |
| D2HGDH - A426T | 120.1405 | 129.3849 | 106.5934 |

2-KG pmol/mg of protein, assay#1, 4 replicates

| | | | | |
|---|---|---|---|---|
| MSCV | 1971.995 | 2196.937 | 2416.924 | 2896.858 |
| D2HGDH - WT | 4719.369 | 4166.875 | 3449.139 | 3894.311 |
| D2HGDH - G131X | 1431.407 | 1766.205 | 1333.068 | 1651.335 |
| D2HGDH - A208T | 1843.989 | 2075.87 | 1531.019 | 1865.886 |
| D2HGDH - R421H | 1466.916 | 1622.627 | 1958.838 | 1030.705 |
| D2HGDH - A426T | 2050.196 | 1964.029 | 1959.822 | 2005.751 |

2-KG pmol/mg of protein, assay#2, 4 replicates

| | | | | |
|---|---|---|---|---|
| MSCV | 1855.615 | 1558.616 | 1744.538 | 2453.218 |
| D2HGDH - WT | 3999.018 | 2828.462 | 2733.508 | 2423.464 |
| D2HGDH - G131X | 238.939 | 539.663 | 480.198 | 578.263 |
| D2HGDH - A208T | 734.603 | 1043.636 | 887.809 | 1088.109 |
| D2HGDH - R421H | 1420.497 | 2164.322 | 1502.578 | 1198.894 |
| D2HGDH - A426T | 1830.906 | 1006.023 | 1047.18 | 1040.221 |

2-KG pmol/mg of protein, assay#3, 5 replicates

| | | | | | |
|---|---|---|---|---|---|
| MSCV | 1025.726 | 768.7465 | 776.5064 | 727.4821 | 1530.187 |
| D2HGDH - WT | 1581.188 | 763.424 | 1491.919 | 1513.157 | 1188.635 |
| D2HGDH - R212W | 740.7486 | 357.0364 | 924.6938 | 297.2062 | 583.04 |

**D2HGDH transient expression cell model**

D2-HG pmol/mg of protein - assay#1, 3 replicates

| | | | |
|---|---|---|---|
| MSCV | 36.64416 | 36.4722 | 40.08173 |
| D2HGDH - WT 0.5ug | 29.68443 | 27.44684 | 32.32854 |
| D2HGDH - WT 0.75ug | 22.03808 | 23.38029 | 27.88385 |
| D2HGDH - WT 1.0ug | 14.61641 | 20.90789 | 11.17389 |

D2-HG pmol/mg of protein - assay#2, 3 replicates

| | | | |
|---|---|---|---|
| MSCV | 31.75372 | 28.76147 | 36.49974 |
| D2HGDH - WT 0.5ug | 19.03621 | 21.51516 | 19.99729 |
| D2HGDH - WT 0.75ug | 16.49129 | 15.69138 | 18.33014 |
| D2HGDH - WT 1.0ug | 12.7291 | 19.3764 | 13.86678 |

2-KG pmol/mg of protein - assay#1, 3 replicates

| | | | |
|---|---|---|---|
| MSCV | 375.6534 | 546.3336 | 671.2131 |
| D2HGDH - WT 0.5ug | 848.2435 | 655.7721 | 779.4605 |
| D2HGDH - WT 0.75ug | 803.1845 | 839.1634 | 980.6399 |
| D2HGDH - WT 1.0ug | 930.7704 | 911.0911 | 893.8879 |

2-KG pmol/mg of protein - assay#2, 3 replicates

| | | | |
|---|---|---|---|
| MSCV | 521.091 | 507.3479 | 521.134 |
| D2HGDH - WT 0.5ug | 820.3983 | 774.3329 | 696.7507 |
| D2HGDH - WT 0.75ug | 814.7245 | 885.1288 | 805.5704 |
| D2HGDH - WT 1.0ug | 845.1482 | 860.8818 | 865.4253 |

**Supplementary Table 12 (continuation)**

**D2HGDH transient siRNA-KD model**

D2-HG pmol/mg of protein - assay#1, 2 replicates

| | | |
|---|---|---|
| si-RNA ctrl | 89.93771 | 80.97425 |
| si-RNA D2#5 | 114.5639 | 142.3544 |
| si-RNA D2#3 | 134.5945 | 137.5139 |

2-KG pmol/mg of protein - assay#1, 2 replicates

| | | |
|---|---|---|
| si-RNA ctrl | 313.5986 | 362.0601 |
| si-RNA D2#5 | 238.3543 | 229.6885 |
| si-RNA D2#3 | 206.4567 | 231.8885 |

L2-HG pmol/mg of protein - assay#1, 2 replicates

| | | |
|---|---|---|
| si-RNA ctrl | 227.8028 | 207.9399 |
| si-RNA D2#5 | 208.3678 | 223.5751 |
| si-RNA D2#3 | 152.5984 | 210.5491 |

**DLBCL cell lines - D2HGDH WT**

D2-HG pmol/mg of protein, 3 biological replicates (each representing the mean of technical triplicates)

| | | | |
|---|---|---|---|
| OCI-Ly1 | 11.94 | 4.19 | 5.49 |
| OCI-Ly4 | 0.08 | 0.52 | 0.59 |
| OCI-Ly18 | 1.56 | 1.21 | 1.31 |
| SU-DHL4 | 5.99 | 0.99 | 0.97 |
| SU-DHL10 | 18.40 | 3.62 | 0.26 |
| SU-DHL2 | 0.02 | 0.52 | 0.10 |
| Farage | 0.20 | 0.62 | 0.41 |
| OCI-Ly8 | 16.41 | 1.12 | 6.32 |
| OCI-Ly10 | 0.25 | 0.75 | 5.56 |
| OCI-Ly19 | 8.25 | 1.28 | 7.16 |

2-KG pmol/mg of protein, 3 biological replicates (each representing the mean of technical triplicates)

| | | | |
|---|---|---|---|
| OCI-Ly1 | 264.38 | 1509.22 | 398.72 |
| OCI-Ly4 | 186.97 | 302.15 | 234.85 |
| OCI-Ly18 | 699.86 | 1232.28 | 1070.77 |
| SU-DHL4 | 67.70 | 737.33 | 607.24 |
| SU-DHL10 | 267.42 | 2265.06 | 843.83 |
| SU-DHL2 | 139.60 | 270.09 | 8952.50 |
| Farage | 98.63 | 331.14 | 572.42 |
| OCI-Ly8 | 81.27 | 834.09 | 4894.93 |
| OCI-Ly10 | 65.09 | 205.27 | 448.81 |
| OCI-Ly19 | 1024.32 | 708.69 | 832.61 |

**DLBCL cell lines - D2HGDH mutant**

D2-HG pmol/mg of protein, 3 biological replicates (each representing the mean of technical triplicates)

| | | | |
|---|---|---|---|
| WSU-NHL | 9.84 | 0.53 | 0.40 |
| SU-DHL6 | 7.54 | 1.32 | 1.18 |
| SU-DHL8 | 12.39 | 0.51 | 0.64 |
| OCI-Ly7 | 0 | 0.69 | 5.40 |

2-KG pmol/mg of protein, 3 biological replicates (each representing the mean of technical triplicates)

| | | | |
|---|---|---|---|
| WSU-NHL | 143.47 | 330.17 | 803.20 |
| SU-DHL6 | 39.79 | 82.87 | 585.57 |
| SU-DHL8 | 171.76 | 309.72 | 783.20 |
| OCI-Ly7 | 339.31 | 1175.34 | 102.27 |

**Supplementary Note 1:**

No somatic variants were reported for D2HGDH in four published sequencing studies on DLBCL, accounting for over 200 patients. Below, we detail the potential reasons for these negative findings.

In Pasqualucci et al[1] only 6 DLBCLs (normal and tumour DNA pairs) were examined by whole-exome sequencing. Subsequently, the authors perform targeted re-sequencing of 56 genes (D2HGDH was NOT one of them) in 48 to 105 additional DLBCL biopsies. In that report, the size of the initial cohort, coupled with the low frequency of DH2HGDH mutations and the selection of a restricted list of candidate gene set for the subsequent analysis, likely explain the absence of D2HGDH variants in their DLBCL collection.

Zhang et al[2] defined their set of significantly mutated genes based on a discovery cohort of 34 paired DLBCL samples. According to their criteria, genes not previously associated with cancer were required to be mutated in at least two samples for inclusion in their list of significant candidates, which would eliminate genes with mutation frequencies lower than 6% (thus including D2HGDH which we found to be mutant in 3.3% of the primary samples or 5.8% primary samples + cell lines). In Zhang et al, the subsequent validation set was only screened for the genes identified in the smaller discovery cohort and thus would not have revealed D2HGDH mutations. Of note, Zhang et al. explored in great detail the degree of overlap between 'DLBCL genes' identified by theirs and the other studies discussed above; they found a surprisingly low concordance among the four studies at all mutation frequency levels, but especially for the genes at the lower end of the frequency spectrum. Remarkably, they noted that 30% of the genes mutated in at least 10% of the cases in their study were not detected by any of the remaining three studies and concluded that genetic heterogeneity in the disease

might contribute to the observed patterns of disparate mutations. The report on the 21 lymphoma cell lines studied by Zhang et al[2] is limited to the “322 DLBCL genes” that the authors identified in the initial 34 paired-samples cohort, and that does not include D2HGDH

The design of the study by Morin et al[5] was powered to pick up mutations at frequencies of 2.8% and higher, which would theoretically be sensitive enough to detect D2HGDH mutations at our estimated frequency of 5.4% (8 mutations in 148 samples – 4 in 120 primary tumors – 3.3%; 4 in 28 DLBCL cell lines 14%). However, in that study coding variants present in the dbSNP were removed at the first step of the analysis. If we apply these criteria to our cohort, 2 variants found in 4 of our primary biopsies (R421H and A426T), would have been excluded, leaving a residual frequency of D2HGDH mutations of 2.7%, which would fall within the margin of error of Morin et al.’s detection. Morin et al. also sequenced 10 DLBCL cell lines, and of those, the only ones that we report as having D2HGDH mutation are SU-DHL-6 and OCI-Ly7. Coincidently, these are exactly the cell lines with the R421H and A426T variants reported in dbSNP, which again would be removed from their analysis. We should indicate here that not only we demonstrated that these variants render D2HGDH inactive, but their frequency in non-malignant tissues (i.e., reference databases) is markedly lower than that of our DLBCL cohort. Finally, as referred to before, the A426T allele has been found to segregate with familial cases of D-2-hydroxyglutaric aciduria, further highlighting its biological relevance.

Lohr et al[8] studied a cohort of 49 normal and tumour DNA pairs. Although they applied algorithms to recognize significantly mutated genes at low frequency, their sample size would put them right at the limit of detection for D2HGDH variants (3.3% based on 4 D2HGDH mutants in 120 primary tumours in our cohort). In other words, all things being equal, they could find ~ 1.6 tumours mutant for D2HGDH in 49 samples. Importantly, the authors recognize that some mutations that may be functionally relevant would not meet statistical significance based

on their algorithms. In fact, by expanding the scale of their genomic analysis and applying novel statistical definitions, the same group recognized that current genomic studies have not yet achieved saturation for cancer gene discovery[9].

**Supplementary References:**


1 Pasqualucci, L. *et al.* Analysis of the coding genome of diffuse large B-cell lymphoma. *Nat Genet* **43**, 830-837, doi:10.1038/ng.892 (2011).

2 Zhang, J. *et al.* Genetic heterogeneity of diffuse large B-cell lymphoma. *Proc Natl Acad Sci U S A* **110**, 1398-1403, doi:10.1073/pnas.1205299110 (2013).

3 Pasqualucci, L. *et al.* Inactivating mutations of acetyltransferase genes in B-cell lymphoma. *Nature* **471**, 189-195, doi:10.1038/nature09730 (2011).

4 Ngo, V. N. *et al.* Oncogenically active MYD88 mutations in human lymphoma. *Nature* **470**, 115-119 (2011).

5 Morin, R. D. *et al.* Frequent mutation of histone-modifying genes in non-Hodgkin lymphoma. *Nature* **476**, 298-303 (2011).

6 Morin, R. D. *et al.* Somatic mutations altering EZH2 (Tyr641) in follicular and diffuse large B-cell lymphomas of germinal-center origin. *Nat Genet* **42**, 181-185, doi:10.1038/ng.518 (2010).

7 Barretina, J. *et al.* The Cancer Cell Line Encyclopedia enables predictive modelling of anticancer drug sensitivity. *Nature* **483**, 603-607, doi:10.1038/nature11003 (2012).

8 Lohr, J. G. *et al.* Discovery and prioritization of somatic mutations in diffuse large B-cell lymphoma (DLBCL) by whole-exome sequencing. *Proc Natl Acad Sci U S A* **109**, 3879-3884, doi:10.1073/pnas.1121343109 (2012).

9 Lawrence, M. S. *et al.* Discovery and saturation analysis of cancer genes across 21 tumour types. *Nature* **505**, 495-501, doi:10.1038/nature12912 (2014).
